# Tumor-Intrinsic Activity of Chromobox 2 Remodels the Tumor Microenvironment in High-grade Serous Carcinoma

**DOI:** 10.1158/2767-9764.CRC-24-0027

**Published:** 2024-08-05

**Authors:** Ritsuko Iwanaga, Tomomi M. Yamamoto, Karina Gomez, Lily L. Nguyen, Elizabeth R. Woodruff, Miriam D. Post, Railey G. Mikeska, Etienne Danis, Thomas Danhorn, Meher P. Boorgula, Siddhartha S. Mitra, Nicole A. Marjon, Benjamin G. Bitler, Lindsay W. Brubaker

**Affiliations:** 1 Division of Reproductive Sciences, Department of Obstetrics and Gynecology, University of Colorado Denver, Anschutz Medical Campus, Aurora, Colorado.; 2 Division of Gynecologic Oncology, Department of Obstetrics and Gynecology, University of Colorado Denver, Anschutz Medical Campus, Aurora, Colorado.; 3 Department of Pathology, University of Colorado Denver, Anschutz Medical Campus, Aurora, Colorado.; 4 University of Colorado Cancer Center, University of Colorado Anschutz Medical Campus, Aurora, Colorado.; 5 Department of Biomedical Informatics, University of Colorado Anschutz Medical Campus, Aurora, Colorado.; 6 Department of Pediatrics, University of Colorado School of Medicine, Aurora, Colorado.

## Abstract

Chromobox 2 (CBX2), an epigenetic reader and component of polycomb repressor complex 1, is highly expressed in >75% of high-grade serous carcinoma. Increased CBX2 expression is associated with poorer survival, whereas CBX2 knockdown leads to improved chemotherapy sensitivity. In a high-grade serous carcinoma immune-competent murine model, knockdown of CBX2 decreased tumor progression. We sought to explore the impact of modulation of CBX2 on the tumor immune microenvironment (TIME), understanding that the TIME plays a critical role in disease progression and development of therapy resistance. Exploration of existing datasets demonstrated that elevated CBX2 expression significantly correlated with specific immune cell types in the TIME. RNA sequencing and pathway analysis of differentially expressed genes demonstrated immune signature enrichment. Confocal microscopy and co-culture experiments found that modulation of CBX2 leads to increased recruitment and infiltration of macrophages. Flow cytometry of macrophages cultured with CBX2-overexpressing cells showed increased M2-like macrophages and decreased phagocytosis activity. *Cbx2* knockdown in the *Trp53*-null, *Brca2*-null ID8 syngeneic murine model (ID8 *Trp53*^−/−^*Brca2*^−/−^) led to decreased tumor progression compared with the control. NanoString immuno-oncology panel analysis suggested that knockdown in Cbx2 shifts immune cell composition, with an increase in macrophages. Multispectral immunohistochemistry (mIHC) further confirmed an increase in macrophage infiltration. Increased CBX2 expression leads to recruitment and polarization of protumor macrophages, and targeting CBX2 may serve to modulate the TIME to enhance the efficacy of immune therapies.

**Significance:**

CBX2 expression correlates with the TIME. CBX2 modulation shifts the macrophage population, potentially leading to an immunosuppressive microenvironment, highlighting CBX2 as a target to improve efficacy of immunotherapy.

## Introduction

High-grade serous carcinoma (HGSC) is the most common histologic subtype of epithelial ovarian cancer. Understood to arise from the fallopian tube epithelium, ovarian surface, or peritoneum, HGSC is one of the most lethal gynecologic malignancies (1–3). Diagnosed at an advanced stage in most patients, HGSC is treated with a combination of surgery and chemotherapy. Although many patients will respond to primary chemotherapy, the disease has a greater than 80% risk of recurrence and subsequent development of chemoresistance (4, 5). All patients with recurrent HGSC will eventually succumb to their disease. Thus, identifying drivers of disease recurrence, progression, and platinum resistance is a critical unmet need in the care of patients with ovarian cancer.

The epigenetic reader and polycomb repressor complex 1 (PRC1) component chromobox 2 (CBX2) is highly expressed in more than 75% of HGSC tumors (6, 7). It is understood that CBX2 is involved in chromatin regulation and gene transcription and, more broadly, PRC1 specifically regulates cell-fate decisions and is essential for normal organismal development of organisms (8). Clinical studies have demonstrated that polycomb proteins are abnormally expressed many malignancies and that they play a role in cancer invasion, metastases, and progression (8). Data from previous reports suggest that increased CBX2 expression in HGSC conveys poorer disease-free and overall survival and is associated with therapy-resistant disease (6). In anchorage-independent settings, CBX2 protects cells from apoptosis via enhancing stem-like properties (6). However, beyond the concept of stemness, little is understood about how CBX2 drives HGSC disease progression.

The tumor immune microenvironment (TIME) plays a critical role in HGSC therapy response. Tumor-associated macrophages (TAM) account for up to 40% of immune cells in HGSC (9), and elevated TAMs are frequently a poor prognostic indicator and contribute to tumor progression (10). Several studies indicate that limited treatment response, and specifically platinum resistance, may be instigated by cytokines released by TAMs, particularly those traditionally defined as M2-like macrophages (11). In contrast, a subset of TAMs is predicted to have antitumor properties, classically defined as M1 (12). In prostate cancer, the PRC1 was linked to elevated CCL2-mediated tumor-promoting macrophage recruitment (13), which supports a potential role of CBX2 in TIME modulation. Although HGSC tumors are hypothesized to be enriched with tumor-promoting TAMs, there is limited understanding about how the complexity of macrophage polarization contributes to tumor progression and antitumor immunity.

Thus, in HGSC models, we sought to define the impact of modulation of CBX2 on the TIME, specifically the impact of macrophage infiltration, polarization, and activity. In human HGSC tumors, elevated CBX2 significantly correlated with macrophage infiltration. *In vitro* and *in vivo* HGSC models highlight that CBX2 expression leads to differential macrophage infiltration and phagocytosis. Given efforts underway to target CBX2 (14, 15), understanding the impact of CBX2 on the TIME and macrophage activity may allow for therapeutic optimization or novel therapeutic combinations.

## Materials and Methods

### Cell culture

As described in our previous work (15), human HGSC cell lines (PEO1 RRID: CVCL_2686, OVCAR4 RRID: CVCL_1627, ID8 Trp53^−/−^ Brca2^−/−^ RRID: CVCL_IU14) were obtained from the Gynecologic Tissue and Fluid Bank, and a syngeneic (murine) cell line (ID8) was generously provided by Dr. Ian McNeish (Imperial College London, London, United Kingdom). All cell lines were authenticated at The University of Arizona Genomic Shared Resource via short tandem repeat profiling. OVCAR4 and PEO1 cells were cultured in RPMI 1640 supplemented with 10% heat-inactivated FBS and 100 U/mL penicillin/streptomycin. ID8 cells were cultured in DMEM supplemented with 4% heat-inactivated FBS, 100 U/mL penicillin/streptomycin, 5 μg/mL insulin, 5 μg/mL transferrin, and 5 ng/mL sodium selenite. All cells were cultured at 37°C in 5% CO_2_. Cells were routinely tested for *Mycoplasma* using LookOut (Millipore-Sigma, Darmstadt Germany), most recently on March 13, 2024.

### Small hairpin RNA knockdown

As described previously (6), CBX2-specific small hairpin RNAs (shRNA) were obtained from the University of Colorado Functional Genomics Facility (human *CBX2* #1: TRCN0000020327 and human *CBX2* #2: TRCN0000232722, Mouse *Cbx2* #1: TRCN0000334429, #2 TRCN0000096264). An empty pLKO.1-puro was utilized as shControl (shCTRL). Plasmid isolation was performed using Plasmid Midi-Prep Kit (Qiagen Hilden, Germany). HEK293T cells were transfected with lentivirus construct with packaging plasmids with Lipofectamine 2000 (Thermo Fisher Scientific, Waltham, MA) following the manufacturer’s instruction. Cells were incubated overnight and transitioned to DMEM the following morning. The viral supernatant was collected 72 hours posttransfection, applied to OVCAR4, PEO1, or ID8 Trp53^−/−^ Brca1^−/−^ for 24 hours with polybrene. Infected cells were selected using 1.0 μg/mL puromycin.

### Small interfering RNA knockdown

As described in ref. 16, cells were plated the day before transfection in RPMI supplemented with 10% FBS but without antibiotics, to be 60% to 80% confluent at the time of transfection. Cells were transfected using Lipofectamine RNAiMAX Reagent (Thermo Fisher Scientific, Cat#13778-075) according to the manufacturer’s protocol. Briefly, for a six-well plate, each well contained 200 µL small interfering RNA (siRNA)–lipid complex in Opti-MEM, including 50 pmol siRNA and 6 µL RNAiMAX. Six hours after transfection, medium was changed to nonantibiotic RPMI. The siRNAs used were *siCXCL1* (Dharmacon on target plus SMART pool, CXCL1 Cat. # L-003898-00-0005), *siCXCL8* (Dharmacon on target plus SMART pool, CXCL8 Cat. # L-004756-00-0005), or negative control siRNA (Dharmacon on target plus nontargeting pool, Cat. # D-001810-10-05).

### CRISPR knockout of CBX2

*CBX2* CRISPR knocked out OVCAR4 cells were created by the University of Colorado Functional Genomics Facility using the IDT Alt-R RNP system. ALT-R crRNA and ATL-R tracrRNA were suspended at 100 μmol/L in nuclease-free IDTE pH7.5. The same volumes of ALT-R crRNA and ATL-R tracrRNA were mixed to prepare gRNA complex at 50 μmol/L, heated at 95°C for 5 minutes, and then were cooled to room temperature. The RNP complex was prepared with 150 pmol of gRNA and 125 pmol of Alt-R Cas9-NLS in final volume of 5 μL in PBS. Cells were harvested in Necleofector solution SF with supplement (Lonza, Basel, Switzerland) at a concentration of 1 × 10^6^ cells per 100 μL. The transfection mix was made with 100 μL of cell suspension, 2.5 μL each of RNP complex, and 0.6 μL of 100 μL of Alt-R Cas9 Electroporation enhancer. For OVCAR4, program FE-132 was used. After pulsing, prewarmed medium was added into the Nucleocuvette vessel, and cells were plated for further culturing and isolating clonal populations. The following guide RNAs were used: Hs.Cas9.CBX2.1.AC:CCGAGTG​CATCCTGAGCAAG and Hs.Cas9.CBX2.1.AA:GAGTACCTGGTCAAG​TGGCG. PCR primers to screen for deletion included CBX2-F1: AGC​ATG​GAG​GAG​CTG​AGC​A, CBX2-R2: GGT​TAC​AGC​GGG​GAG​AAT​CTG, and CBX2-R3: GGA​GAA​TCT​GGC​CAA​GAG​GAG.

### CBX2 overexpression

Full-length *CBX2* (NM_005189) cDNA was cloned from OVCAR4 cDNA library, made by oligo dT primer and SuperScriptIII (Thermo Fisher). Full-length *CBX2* was amplified using primers Fwd GGT GCT TTG TGT GCT GC and Rev TCA GTA ATG CCT CAG GTT GAA G. Using the PCR product above as a template, a restriction enzyme compatible clone was amplified with nested PCR using primers. For full-length, XbaI Fwd ATT​TCT​AGA​ATG​GAG​GAG​CTG​AGC​AGC​G.

EcoRI Rev ATT​GAA​TTC​TCA​GTA​ATG​CCT​CAG​GTT​GAA​G. PCR products were gel-purified, cut with restriction enzyme XbaI/EcoRI or EcoRI/NotI, and ligated into pCDH-CMV-MCS-EF1-Puro vector that had been cut with XbaI/EcoRI or EcoRI/NotI. Positive clones were confirmed by Sanger sequencing, transformed into Stbl3-competent cells (Thermo Fisher), and amplified with Qiagen MIDI plasmid preparation. Lentiviral particles were generated by transfecting pCDH-CMV-MCS-EF1-Puro-CBX2 into 293FT cells, and viral particles were collected. OVCAR4 cells were transduced with pCDH-CMV-MCS-EF1-Puro or pCDH-CMV-MCS-EF1-Puro-CBX2. Cells were selected with 1 μg/mL puromycin for 48 hours and CBX2 expression was confirmed via qPCR and immunoblot.

### Immunoblotting

As described in ref. 17., protein was extracted with radioimmunoprecipitation assay buffer (150 mmol/L NaCl, 1% Triton X-100, 0.5% sodium deoxycholate, 0.1% SDS, and 50 mmol/L Tris pH 8.0) supplemented with complete EDTA‐free protease inhibitors (Roche, Cat. #4693132001), 5 mmol/L NaF, and 1 mmol/L Na_3_VO_4_. Nuclear extraction was performed by suspending cells in a hypotonic buffer (10 mmol/L HEPES-KOH pH 7.9, 1.5 mmol/L MgCl_2_, 10 mmol/L KCl, 1 mmol/L DTT, and 1× halt protease inhibitor). After dounce homogenization and centrifugation, the resulting nuclear pellets were suspended in a hypertonic buffer (20 mmol/L HEPES-KOH pH 7.9, 25% glycerol, 1.5 mmol/L MgCl_2_, 0.6 mol/L KCl, 0.2 mmol/L EDTA, 1 mmol/L DTT, and 1× halt protease inhibitor). Protein was separated on an SDS-PAGE and transferred to the polyvinylidene fluoride membrane. Incubation with primary antibodies anti-CBX2 (Diagenode, Cat #C15410339, RRID: AB_3099491) and anti–β-actin (Abcam Cat# ab6276, RRID: AB_2223210) was performed overnight at 4°C. Secondary goat anti‐rabbit (IRDye 680RD or IRDye 800CW, LI‐COR, Cat. #92568071; RRID: AB_2721181 or Cat. #926‐32211; RRID: AB_621842; 1:20,000) and goat anti‐mouse (IRDye 680RD or IRDye 800CW, LI‐COR, Cat. # 926‐68070; RRID: AB_10956588 or Cat# 925‐32210; RRID: AB_2687825; 1:20,000) antibodies were applied for 1 hour at room temperature. Blots were visualized using the LI‐COR Odyssey Imaging System and Image Studio software (V4).

### RNA sequencing and analysis

Total RNA was extracted using the Quick-RNA Miniprep Plus Kit (Zymo Research, Cat. #D4209) from cells cultured in attachment settings. Ribosome depletion and library preparation were performed using the Qiagen FastSelect (Cat. #334375) and KAPA BioSystems mRNA HyperPrep Kit (Cat. #KK8581) according to the manufacturer’s protocols. Briefly, 500 ng of RNA was used as the input, and KAPA BioSystems single-index adapters were added at a final concentration of 1.5 mmol/L. A purified, adapter-ligated library was amplified following the manufacturer’s protocol. The final libraries were pooled and sequenced on an Illumina NovaSeq 6000 (University of Colorado Genomics Core; RRID: SCR_021984) as 150-bp paired-end reads.

RNA sequencing (RNA-seq) data analysis was performed by the Biostatistics and Bioinformatics Shared Resource of the University of Colorado Cancer Center (RRID: SCR_021983). FASTQ files generated using bcl2fastq (version 2.20.0.422) were processed using the nf-core/rnaseq pipeline (version 3.9, https://nf-co.re/rnaseq/3.9; ref. 18). Sequencing adapters were removed using Cutadapt (version 3.4; ref. 19) as part of the Trim Galore package (version 0.6.7). Reads were aligned using STAR (version 2.7.10a; ref. 20) to the human transcriptome (GRCh38, gene annotation from Ensembl release 104). Gene quantification was performed using Salmon (version 1.5.2; ref. 21). Tables with transcript and gene counts and TPM were generated using the tximport R package (version 1.18.0; ref. 22) under R version 4.0.3. Normalized data were processed to counts per million (23). Differential expression was calculated using the limma R package (24). Heatmaps were generated by Microsoft Excel based on the 10^th^ and 90^th^ percentile of expression.

Pathway analysis of the differentially expressed genes that overlapped between CBX2 knockout and overexpression was conducted via gene set enrichment analysis and overlap with hallmark pathways.

### *In vivo* model

All mouse experiments were approved by the Institutional Animal Care and Use Committee (IACUC protocol #569). Six- to eight-week-old C57BL/6J mice were purchased from The Jackson Laboratory (strain #000664), and each mouse was intraperitoneally injected with 1 × 10^6^ ID8 *Trp53*^−/−^*Brca2*^−/−^ cells, which either had *Cbx2* knocked down using lentivirus (shCbx2 #1 and shCbx2 #2) or were treated with an empty lentivirus (shCTRL) leaving *Cbx2* intact, as described above. Thirty-six days after cell injection, mice were euthanized via CO_2_ inhalation and cervical dislocation and necropsied. Omental tissue was resected for further processing including multispectral IHC (mIHC) and NanoString.

### Multispectral IHC (mIHC) and image analysis

mIHC analyses were performed using Vectra Automated Quantitative Pathology Systems (Akoya Biosciences, Menlo Park, CA) as described previously (25). Tissues were formalin-fixed, paraffin-embedded, and sectioned at four microns onto slides. Slides were sequentially stained with antibodies specific for F480 (Cell Signaling Technology, Cat. #30325S) and DAPI. All antibody details are provided in Supplementary Table S1. All slides were de-identified and imaged by the Human Immune Monitoring Shared Resource core (RRID: SCR_021985) on Akoya Biosciences Vectra Polaris scanner. Regions of interest were selected, and multispectral images were collected with a 20x objective. A training set of nine representative images was used to train analysis algorithms for tissue and cell segmentation and phenotyping using inForm software (Akoya Biosciences, Menlo Park, CA). Representative autofluorescence was measured on an unstained control slide and subtracted from study slides. Total tumor area, total cell count, and cell densities of positive and negative cells for each phenotype were graphed and compared in GraphPad Prism 9. Statistical analyses were performed using a multiple comparison one-way ANOVA test.

### Quantitative PCR (qPCR)

RNA was isolated using RNeasy Plus Mini Kit (Qiagen, Hilden Germany) according to the manufacturer’s protocol. NanoDrop spectrophotometry was used to measure concentration of extracted RNA. Luna Universal One-step RT qPCR kit (New England BioLabs, Ipswich, MA) was used on Bio-Rad CFX96 (Bio-Rad, Hercules, CA). HPRT or GAPDH were used as internal control as stated in the figure legend. All primer sequences are in Supplementary Table S2.

### NanoString and analysis

As described in ref. 16, formalin-fixed, paraffin-embedded omental tumors from the shCTRL and shCbx2 ID8 study were sectioned, and 10-micron tissue–containing paraffin scrolls were collected. RNA was extracted using the High Pure FFPET RNA Isolation kit (Roche) according to the manufacturer’s instructions and eluted in 40 μL elution buffer. RNA quantity and quality were assessed using an RNA ScreenTape on TapeStation 4150 (Agilent). The concentration of RNA was determined by comparison with the RNA ladder.

Extracted RNA was diluted to 30 ng/μL and 5 μL (150 ng) was combined with hybridization buffer and the Reporter CodeSet for the Murine PanCancer IO 360 Panel (NanoString) and incubated for 20 hours at 65°C. The hybridized reaction was analyzed on an nCounter SPRINT Profiler (NanoString). nSolver calculated normalization factors for each sample using raw gene counts and 14 housekeeping genes. Differential gene expression was calculated from normalized gene counts data and an FDR with a Benjamini–Hochberg multicomparison test.

An nSolver Advanced Analysis tool was used for pathway analysis of the genes expressed in shCTRL (*n* = 3) and shCbx2 (*n* = 7) samples. The expression profile of the genes expressed was used to generate a pathway score for 25 different pathways (e.g., hypoxia). Genetic signature analysis was performed by NanoString and previously described in ref. 26–30. NanoString counts are available in Supplementary Table S3.

### Human primary monocyte isolation

Healthy female donor blood samples (age 21–50 years, in total 25 unique donors) were obtained from the Children’s Hospital Colorado Blood Donor Center at the University of Colorado Anschutz Medical Campus. Collections were conducted according to ethical guidelines (Declaration of Helsinki). Participants were consented with verbal and written consent at the Children’s Hospital Colorado Blood Donor Center. Blood was isolated from leukocyte reduction system (LRS) chambers, which is considered a waste product from blood donor collections. The use of LRS chambers as a discarded material is covered in the consent signed by donors when they give blood. Serum was collected from LRS chambers, and peripheral blood mononuclear cells (PBMC) were isolated using a Leucosep tube (STEMCELL Technologies, Vancouver, Canada) with Ficoll-Paque (Cytiva, Marlborough, MA). The cells were treated with red blood cell lysis buffer (0.832% NH_4_Cl, 0.1% NaHCO_3_, and 0.02% EDTA) for 30 minutes at room temperature. Monocytes were isolated using EasySep Human Monocyte Isolation Kit (STEMCELL Technologies, Vancouver, Canada) following the manufacturer’s protocol.

### Primary monocytes direct co-culture/invasion with cancer cells

OVCAR4 cells were stained with CellTracker Deep Red (#C34565 Invitrogen, Waltham MA) for 30 minutes. A total of 2 × 10^5^ stained cells per well were seeded in six-well ultralow attachment plates (#3471 Corning Inc., Corning NY) in 1 mL CSC media (DMEM/F12, 1x B27, 4% FBS, 100 U/mL penicillin/streptomycin, 20 ng/mL human EGF, and 20 ng/mL human FGF; ref. 31). On day 3, human primary monocytes were stained with CellTracker Green CMFDA (#C7025, Invitrogen, Waltham MA) for 30 minutes, and 50,000 cells per plate were added to the spheres in 1 mL CSC media and cultured for an additional 7 days (CSC media were added every 3 days). For flow cytometry analysis, after 7 days of co-culture, the cells were collected using 40-micron filter, washed with PBS, and then digested with TrypLE (Gibco, Billings, MT) to make single cells. The cells were analyzed using Penteon (NovoCyte Agilent, Santa Clara, CA), and cytometry data were analyzed using FlowJo software (Tree Star, Ashland, OR). The picture images were taken using a Zeiss Axio Observer Z1 inverted microscope (Zeiss software Rel. 4.8) and quantified using CellProfiler 2.2.0 software.

### Indirect polarization assay

Primary monocytes were cultured for 1 day with monocyte medium (RPMI 1640, 10% heat-inactivated FBS, 100 U/mL penicillin/streptomycin, 2.5 mmol/L HEPES buffer, 2 mmol/L GlutaMAX) at 37°C in 5% CO_2_. The assay was performed using a transwell chamber (0.4 μm pore size PET membrane) in 12-well plates (Corning, Corning NY). A total of 4 × 10^5^ primary monocytes were plated in the bottom of the plate in 1 mL of 1% FBS monocyte medium with human M-CSF 50 ng/mL (PeproTech, Cranbury, NJ). A total of 1 × 10^5^ monolayer OVCAR4 cells were plated in the transwell chamber in 500 μL of 1% FBS monocyte medium. The monocytes were cultured for 8 to 10 days; OVCAR4 cells were replaced every 3 to 4 days. As controls, INFγ (20 ng/mL) and LPS (100 ng/mL) were used for polarizing M1 macrophages; 50 ng/mL IL4, IL10, and IL13 (PeproTech, Cranbury, NJ) were used for polarizing M2 macrophages.

### Phagocytosis assay

As similar to that described above, 2 × 10^5^ primary monocytes were cultured in 200 μL 1% heat-inactivated FBS monocyte medium (RPMI 1640, 100 U/mL penicillin/streptomycin, 2.5 mmol/L HEPES buffer, and 2 mmol/L GlutaMAX) with 50 ng/ml M-CSF with cancer cells in the transwell chamber (0.4 μm pore size PET membrane) in 24 wells for 10 days. A total of 4 × 10^4^ monolayer OVCAR4 cells were plated in the transwell chamber in 500 μL of 1% FBS monocyte medium. The cancer cells in transwell chamber were replaced every 3 to 4 days. A total of 30 μL FITC-conjugated microbeads were added directly in the TC plate and incubated at 37°C in 5% CO2 for 90 minutes. After the incubation, the supernatant was removed and 200 μL fixation buffer (BD Cytofix Fixation buffer) added and then incubated on ice for 20 minutes. The cells were gently scraped and filtered with 40-μm filter, analyzed by Penteon (NovoCyte Agilent, Santa Clara, CA).

### Flow cytometry

As previously described (32, 33), prior to the staining, cells were treated with Fc blocker (#163404 BioLegend, San Diego, CA) for 20 minutes on ice to block nonspecific binding of antibodies. The cells were incubated with the antibodies for 30 minutes (Supplementary Table S1). Subsequently, cells were washed twice with buffer (2% FBS HBSS). Stained cells were analyzed using Penteon (Agilent, Santa Clara, CA); cytometry data were analyzed using FlowJo software (Tree Star, Ashland, OR).

### Publicly available datasets

The Cancer Genome Atlas (TCGA) Ovarian Serous Cystadenocarcinoma (PanCancer Atlas) was examined for CBX2 mRNA expression. Various imputation analyses were performed, including XCELL, CIBERSORT, CIBERSORT-ABS, EPIC, QUANTISEQ, and TIDE, using the TIMERv2 platform (34). Carcinoma EcoTyper analysis was performed to describe cell types (35). Raw data for TIMERv2 analysis using different imputation models are available in Supplementary Table S4.

### Statistical analysis

All statistical analyses were conducted in Prism GraphPad v9. Survival comparison used Kaplan–Meier with log-rank, pairwise comparison used *t* test, multicomparison used ANOVA with multiple test correction, and pairwise comparison over time was analyzed using a mixed model effect. A *P* < 0.05 was considered significant and when required FDR multicomparison correction (*q* < 0.05) was made. Error bars are shown as standard error of the mean (SEM). All *in vitro* experiments were performed in triplicate, and the *in vivo* experiment was run in duplicate.

### Data availability

All data are available within the article and its supplementary materials. RNA-seq data sets are available from the Gene Expression Omnibus (https://www.ncbi.nlm.nih.gov/geo/) under the accession number GSE251947.

## Results

### CBX2 regulates immune transcriptional profiles

**Figure 1:**
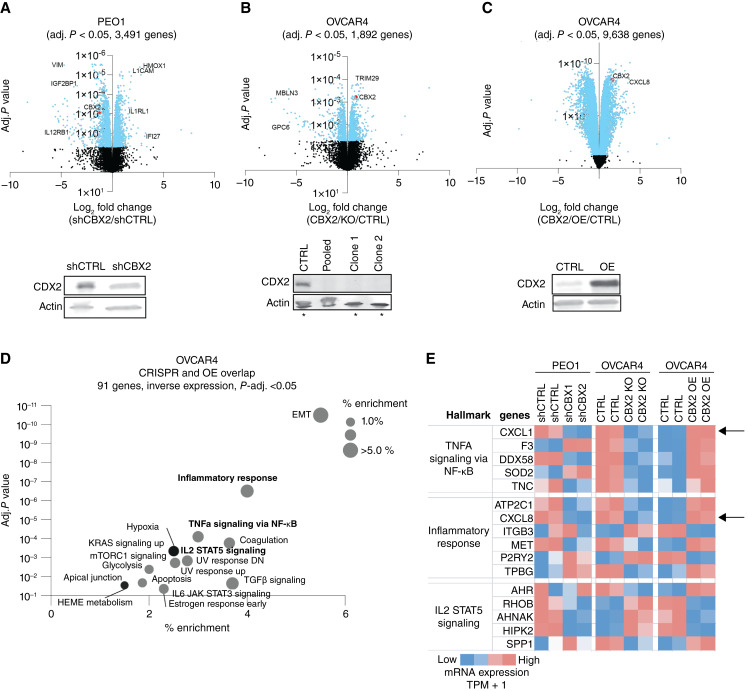
CBX2 regulates immune transcriptional profiles. **A,** Volcano plot of differential gene expression based on shCBX2 vs. shCTRL in the PEO1 cell line. Immunoblot against CBX2 in PEO1 shCTRL and shCBX2 cells. Loading control, β-actin. **B,** Volcano plot of differential gene expression based on CBX2 KO vs. CTRL in the OVCAR4 cell line. Immunoblot against CBX2 in OVCAR4 CTRL and *CBX2* KO. Loading control, β-actin. Asterisks indicate cell lines used for RNA-seq. **C,** Volcano plot of differential gene expression based on *CBX2* OE vs. CTRL in the OVCAR cell line. Immunoblot against CBX2 in OVCAR4 control (CTRL) and *CBX2* OE. Loading control, β-actin. **D,** Overlap of OVCAR4 *CBX2* CRISPR knockout vs. OVCAR4 *CBX2* OE. Inverse expression top hits: EMT and inflammatory response. **E,** Evaluation of hallmark pathways identified TNFα signaling via NF-κB inflammatory responses, and IL2 STAT5 signaling pathways. Direct relationship between *CBX2* status and *CXCL1* and *CXCL8* (IL8) noted (black arrows).

To understand the regulatory role of CBX2, RNA-seq was performed on multiple *CBX2*-modulated HGSC cell lines. RNA-seq was first performed in *CBX2* knockdown (shCBX2) versus *CBX2* intact (shCTRL) in the PEO1 cell line. CBX2 knockdown was confirmed via immunoblotting (Fig. 1A). Differential expression analysis identifies decreased expression of immune regulatory genes *VIM, IGF2BP1*, and *IL12RB1* and increased expression of *L1CAM, IL1RL1*, and *IFI27* in shCBX2 cells compared with shCTRL cells (Fig. 1A). RNA-seq was similarly performed on OVCAR4 cell lines, comparing *CBX2* CRISPR knockout (CBX2 KO) with *CBX2* intact. *CBX2* KO was confirmed via immunoblotting (Fig. 1B). In *CBX2* KO OVCAR4 cells compared with *CBX2* intact (CTRL), there was differential expression of *MBLN3*, *GPC6*, and *TRIM29* (Fig. 1B). When the OVCAR4 cell line with *CBX2* overexpression (CBX2 OE) was compared with CTRL, there was differential expression of *PRMT6*, *IGFL1*, and *CXCL8* (Fig. 1C). *CBX2* overexpression was confirmed via immunoblotting (Fig. 1C). A hypergeometric overlap analysis was performed on OVCAR4 *CBX2* KO and *CBX2* overexpression (CBX2 OE) overlap (adj. *P* < 0.05, opposite direction), identifying 91 genes with statistically significant inverse expression. Pathway analysis demonstrated that epithelial–mesenchymal transition (EMT), inflammatory response, TNFα signaling, and IL2/STAT5 signaling are among the top hits enriched in CBX2-overexpressed cells compared with knockout (Fig. 1D; Supplementary Fig. S1A). Additionally, this analysis demonstrated that EMT is one of the most enriched pathways in CBX2 overexpressed cells, consistent with existing literature, thus serving as confirmation of our findings (36). Specific genes from three highlighted hallmark pathways highlight consistent CBX2-mediated regulation across three independent models, including PEO1 *CBX2* knockdown versus control, OVCAR4 *CBX2* CRISPR knockout versus control, and OVCAR4 *CBX2* overexpression versus control. Specifically, we found that CXCL1, CXCL5, and CXCL8 were directly associated with CBX2 expression (Fig. 1E; Supplementary Fig S1B).

We subsequently analyzed an existing murine-derived CBX2-directed CUT&RUN (cleavage under targets and release using nuclease) dataset (GSE210367). Compared with IgG and HA protein-tagged controls, we observed distinct CBX2-binding sites proximal to numerous genes, including *CXCL1* and *CXCL5* (Supplementary Fig. S1C). Of note, there is no murine *CXCL8* for comparison, as no homologous murine CXCL8 equivalent exists in mice. Overall, interrogating the 91 overlapping genes identified from the transcriptomic analysis against the CBX2 chromatin profiling, we identified 62.4% of genes (53 of 85, six genes were not found in the mouse) with distinct CBX2-binding sites within 10 kilobases of the promoter region. Taken together, the transcriptional evaluation of CBX2-modulated cell lines identified multiple immune-related signatures, and both the transcriptional evaluation and CUT&RUN analysis specifically found a direct relationship between *CBX2* expression and *CXCL1*, *CXCL5*, and *CXCL8* expression, highlighting the potential regulatory role of CBX2.

### CBX2 regulates immune-related pathways in a tumor cell–intrinsic fashion

**Figure 2:**
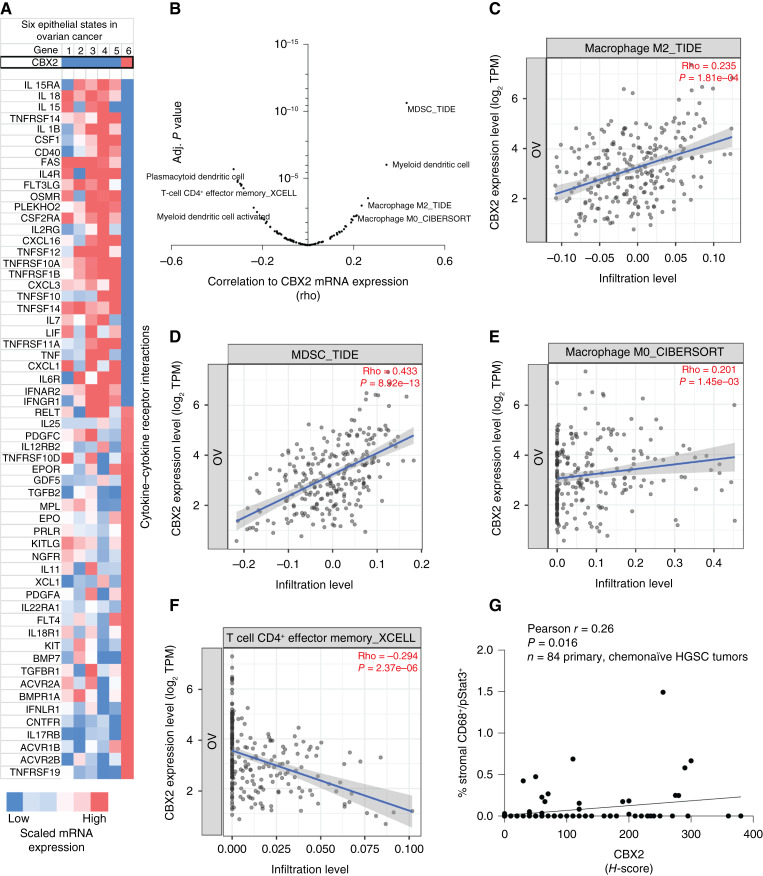
CBX2 regulates immune-related pathways in a tumor cell–intrinsic fashion. **A,** Carcinoma EcoTyper epithelial states (S1–6) compared with CBX2 expression (top panel) and cytokine–cytokine receptor interaction pathway. **B,** Correlation (*x*-axis, rho) of CBX2 mRNA expression in 303 HGSC tumors with imputed cell type. Adjusted *P* value shown on *y*-axis. Imputation pipeline (e.g., CIBERSORT and TIDE) utilized follows underscore in title of plot (details included in Supplementary Table S4). **C,** Scatter plot of CBX2 mRNA expression and macrophage M2_TIDE. **D,** Scatter plot of CBX2 mRNA expression and MDSC_TIDE. **E,** Scatter plot of CBX2 mRNA expression and Macrophage M0_CIBERSORT. **F,** Scatter plot of CBX2 mRNA expression and T cell CD4^+^ effector memory_XCELL. **G,** An annotated tissue microarray was used to perform mIHC evaluating CBX2 expression and immune cell types. Plot demonstrating the relationship between % stromal CD68^+^/pStat3^+^ and CBX2 *H*-score. Statistical tests, unpaired *t* test (**C**) and Pearson correlation (**D–G**).

To understand the impact of CBX2 on the TIME of human HGSC tumors, we next examined the data derived from TCGA and the Carcinoma EcoTyper. Upon initial analysis, we discovered that CBX2 expression identifies a unique tumor cell type and its expression is closely associated with “Epithelial State 6” (Fig. 2A). State 6 is defined by increased expression of CBX2 and accounts for 30% of all tumor cells in HGSC tumors, thus contributing to one of the largest proportions of cells (Fig. 2A; Supplementary Fig. S2A). Like the RNA-seq data presented in Fig. 1, Epithelial State 6 also strongly correlates with hallmark pathway cytokine–cytokine interaction. Note, unlike the *in vitro* data, State 6 is associated with lower CXCL1 expression, suggesting a context-dependent regulation of CXCL1. Taken together, tumors with elevated CBX2 expression are significantly correlated to a differential TIME.

Considering our tumor cell–intrinsic data, TCGA data, and that modulation of CBX2 is directly involved in immune transcriptional signatures, we evaluated CBX2 expression using immune cell imputation analyses (e.g., CIBERSORT, XCELL, and TIDE) to define the correlation between CBX2 expression and multiple immune cell types in 303 ovarian cancer tumors within TCGA database (37). We identified several immune cell types that correlated with high CBX2 expression, including M0 macrophages, M2 macrophages, myeloid-derived stem cells (MDSC), as well as negative correlation to T cells (Fig. 2B–F; Supplementary Table S4). In addition, CIBERSORT analysis of M0 macrophages identified a positive correlation with CXCL5 and CXCL8 (Supplementary Fig. S2B).

Given that the imputation data depend on mRNA expression from TCGA, we used a well-annotated tissue microarray of HGSC tumors to further explore the density and distribution of immune cells (38). Vectra Polaris mIHC allowed for identification and quantification of CD8 T cells (CD3^+^/CD8^+^), T regulatory (Treg) cells (CD3^+^/FOXP3^+^), and total macrophages (CD68^+^) within the HGSC microenvironment, and there was no correlation with CBX2 expression (Supplementary Fig. S2C). Importantly, however, high CBX2 expression in chemonaïve HGSC tumors was positively correlated with *active* macrophages (CD68^+^/pSTAT3^+^; *r* = 0.26; *P* = 0.016; Fig. 2G). Altogether, we found direct links between CBX2 and the TIME using both transcriptional and protein analyses, and we specifically observed correlations between CBX2 and M0, M2, and active macrophages, as well as MDSCs. We next sought to understand CBX2-dependent macrophage recruitment and polarization.

### Modulation of CBX2 leads to a shift in macrophage recruitment

**Figure 3:**
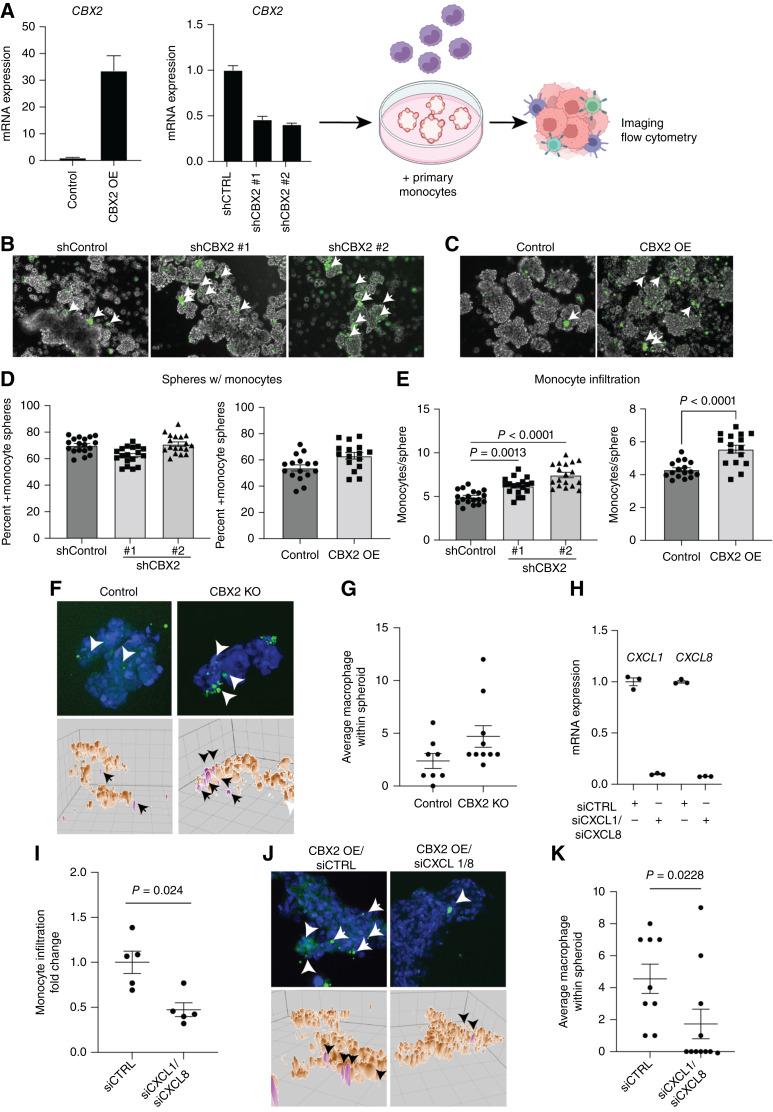
Modulation of CBX2 leads to shift in macrophage recruitment. **A,** OVCAR4 *CBX2* OE and knockdown confirmation by qPCR. Diagram of protocol with direct co-culture of spheroids with primary monocytes. **B,** Confocal microscopy with infiltration of monocytes (green) increased with knockdown of *CBX2*. **C,** Confocal microscopy with infiltration of monocytes increased with overexpression of *CBX2*. **D,** Plots demonstrating the percentage of spheres with monocytes in shCBX2 or overexpression constructs. **E,** Plots demonstrating the number of spheres with CellTracker-positive monocytes in shCBX2 and overexpression. **F,** OVCAR4 (control) and CBX2 KO cells co-cultured with fluorescence-tagged primary monocytes (green, white arrowheads). Blue = nuclei. Images (top) are maximum projections of confocal z-stack and form-filling rendering [brown, cancer cells; pink, monocytes (black arrowheads)]. 50-micron grid. **G,** Quantification of **F**. **H,** qPCR of CXCL1 and CXCL8 in siControl (siCTRL) and siCXCL1/siCXCL8. Internal control, HPRT. **I,** Flow cytometric analysis of digested co-culture spheroids to measure monocyte infiltration. **J,** Same as F, but with siControl and siCXCL1/8 in cells overexpressing CBX2. **K,** Quantification of J. Error bars, SEM. Statistical test, unpaired *t* test and multicomparison ANOVA.

In an *in vitro* system, we wanted to determine if there is differential macrophage recruitment with changes in *CBX2* expression. OVCAR4 cells with *CBX2* overexpression (OE) construct or with *CBX2* knockdown (shCBX2#1 and shCBX2#2; Fig. 3A) were cultured in low attachment setting to form spheroids over 2 days. In parallel, monocytes (CD14^+^CD16^−^) were isolated using negative selection from peripheral blood from healthy donors and were stained with CellTracker. Stained monocytes were then added to the spheroid cultures to monitor infiltration (Fig. 3A). After 5 days in co-culture, monocyte infiltration into the spheroids was observed with confocal microscopy (Fig. 3B and C). Although we noted that there was no difference in the number of spheroids with CellTracker-positive monocytes (Fig. 3D), we did observe that modulating *CBX2* expression led to a significant increase in the number of CellTracker-positive monocytes in spheroids (Fig. 3E; Supplementary Fig. S3A–S3C). Using the *CBX2* KO OVCAR4 cells, we examined spheroid infiltration via confocal microscopy and observed that compared with control, *CBX2* knockout led to a modest increase in macrophage infiltration (Fig. 3F–G). These data suggest that *CBX2* regulates macrophage infiltration.

Based on transcriptomic findings that *CBX2* potentially regulates *CXCL1* and *CXCL8* expression, we performed a combination siRNA-mediated knockdown of *CXCL1* and *CXCL8* in *CBX2* OE cells (Fig. 3H). Using co-culture spheroid as noted above, we examined macrophage infiltration via flow cytometry and confocal imaging. Compared with siControl, siCXCL1/8 spheroids showed a significant reduction in macrophage infiltration (Fig. 3I–K), suggesting cancer cell CBX2-mediated regulation of CXCL1/8 expression contributes to differential macrophage infiltration. Overall, we observed that *CBX2* modulation led to differential macrophage infiltration, which led us to question the CBX2-dependent polarization status of the macrophages.

### CBX2 overexpression increases tumor-promoting macrophages and phagocytic activity

**Figure 4:**
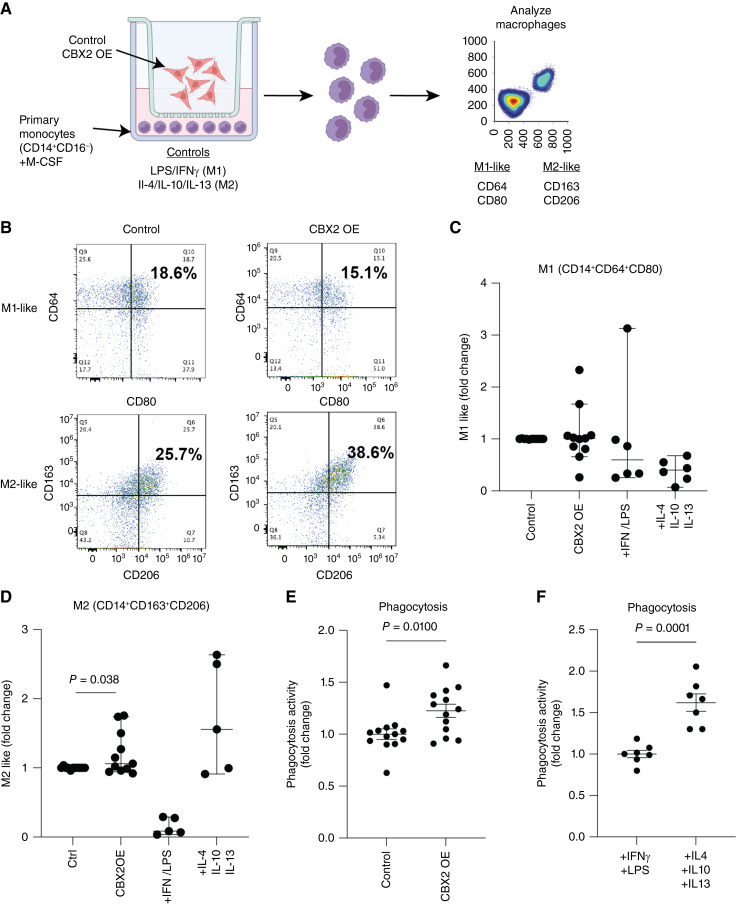
Overexpression of *CBX2* leads to an increase in M2-like macrophage activity. **A,** Diagram of co-culture experimental setup with OVCAR4 *CBX2* OE exposed to primary monocytes (from eight unique donors), then analyzed by flow utilizing M1- and M2-like markers. **B,** Flow cytometry analysis of *CBX2* OE compared with control, increase in M2-like macrophages (CD163 and CD206). **C** and **D,** Plots of the fold change of M1- and M2-like receptors in control, *CBX2* OE, +LPS/INFγ, and IL4/IL10/IL13, in which IL4/IL10/IL13 serves as the control for M2-like stimulation. **E,** Functional phagocytosis assay of monocytes co-cultured with control OVCAR4 compared with *CBX2* OE. **F,** Functional phagocytosis assay, +LPS (M1 stimulatory) compared with +IL4, +IL10, and +IL13 (M2 stimulatory). Error bars, SEM. Statistical test, unpaired *t* test. Note: Each dot represents a unique PBMC donor.

Macrophages exist classically on a spectrum of differentiation states from M1 to M2. Within the tumor microenvironment (TME), M1 macrophages are proposed to be tumor-suppressive and M2 macrophages tumor-promoting; however, the expression of M1 and M2 protein markers is not mutually exclusive for each type and the complexity of this spectrum remains a significant focus in the field (39–41). In HGSC tumors, the differentiation status of macrophages is important, as elevated M1-like macrophages and depleted M2-like macrophages in the TIME correlate to improved overall survival (Supplementary Fig. S4A). These findings highlight the complex relationship between macrophages and tumor progression. Thus, we sought to assess CBX2-dependent M1/M2-mediated polarization and phagocytosis activity. Using an indirect co-culture system, OVCAR4 control and *CBX2* OE cells were cultured with primary human M-CSF-stimulated monocytes. The indirect system only allows for the passage of metabolites and cytokines between the cancer cells and monocytes (Fig. 4A). LPS/IFNγ and IL4/IL10/IL13 were used as controls to differentiate the monocytes into M1- or M2-like macrophages, respectively. After 8 to 10 days of co-culture, macrophage polarization was assayed via flow cytometry. Although *CBX2* OE in the cancer cells did not alter the density of M1-like macrophages (18.6% vs. 15.1%), it did promote the expression of M2-like macrophage markers (Control: 25.7% vs. CBX2 OE: 38.6%, Fig. 4B–D; gating strategy; Supplementary Fig. S4B). In co-culture conditions with monocytes and CBX2-modulated cancer cells, compared with the control cells, we observed that *CBX2* overexpressing cells increased the number of CD68-positive cells, and conversely, *CBX2* knockdown and knockout attenuated the number of CD68 positive cells (Supplementary Fig. S4C), suggesting that *CBX2* expression in the cancer cells is promoting monocyte differentiation into macrophages. Moreover, these data highlight the impact of increased CBX2 expression in HGSC cells in driving a more M2-like macrophage polarization.

Phagocytosis, or the engulfment cells and debris within the microenvironment, is a critical activity of macrophages. Phagocytosis can enhance the presentation of antigens to drive further engagement of the immune system; however, in malignancy increased phagocytosis of tumor cells has also been linked to driving immune suppression and tolerance (42). Using macrophages generated via the indirect co-culture system described above, we observed that compared with macrophages co-cultured with OVCAR4 cells in a Boyden chamber, macrophages co-cultured with OVCAR4 *CBX2* OE cells demonstrated elevated phagocytosic activity (Fig. 4E and F; Supplementary Fig. S4D). Taken together, elevated CBX2 expression leads to the recruitment of macrophages and increased macrophage phagocytic activity.

### Knockdown of Cbx2 in a syngeneic mouse model of ovarian cancer shifts the composition of the TIME

**Figure 5:**
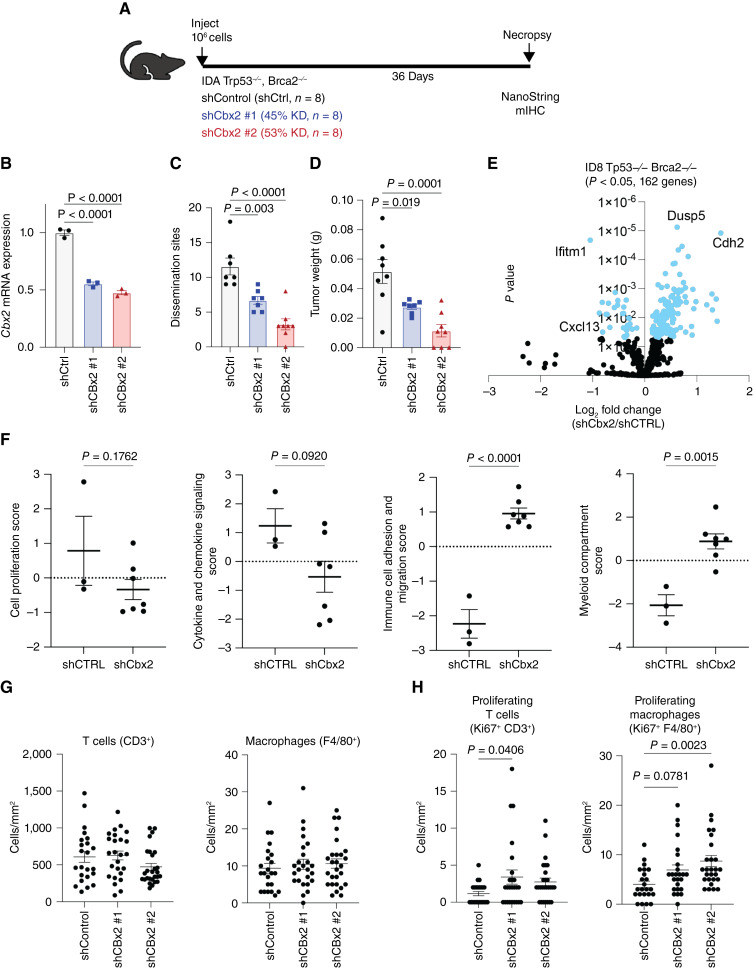
Knockdown of *Cbx2* in a syngeneic mouse model of HGSC leads to shift in composition of the TIME. **A,** Diagram of ID8 syngeneic mouse model. **B,** RT-PCR analysis confirming *Cbx2* knockdown in ID8 *Trp53*^−/−^*Brca2*^−/−^ cells (internal control, 18S), **C,** Tumor dissemination sites and (**D**) total tumor weight of knockdown vs. control tumors. **E,** NanoString transcriptomic differential expression of shCbx2 compared with control, derived from solid tumors. **F,** NanoString pathway scores for cell proliferation, cytokine signaling score, immune cell and migration score, and myeloid comparison score between control and shCbx2. **G,** Comparison of CD3^+^ (T cells) and F480 (macrophages) without Ki67 positivity and (**H**) with Ki67 positivity in control vs. shCbx2. Error bars, SEM. Statistical test, unpaired *t* test and multicomparison ANOVA.

Utilizing a *Trp53*- and *Brca2*-null ID8 syngeneic mouse model (ID8 *Trp53*^−/−^*Brca2*^−/−^; ref. 43), we next examined the effect of *Cbx2* knockdown on tumor progression (Fig. 5A) with three experimental arms: *Cbx2* intact (shCTRL), *Cbx2* knockdown #1 (shCbx2#1), and *Cbx2* knockdown #2 (shCbx2#2). *Cbx2* mRNA expression was evaluated with a statistically significant decrease in *Cbx2* expression in shCbx2 #1 (45% knockdown) and shCbx2 #2 (53% knockdown) confirming appropriate knockdown (Fig. 5B). All mice were injected with 1 × 10^6^ tumor cells, which were allowed to grow for 36 days at which time euthanasia and necropsy were performed. Relative to shCTRL, the number of dissemination sites and total tumor weight were significantly reduced in the *Cbx2* knockdown tumor-bearing mice, and tumor weight was measured with a similar significant decrease in both knockdown lines (Fig. 5C and D). These effects were reproduced in a biological replicate (Supplementary Fig. S5A–S5F). These data confirm that loss of *Cbx2* is sufficient to significantly decrease tumor growth in an immune-competent syngeneic murine model of HGSC.

NanoString transcriptomic analysis was performed on the tumor specimens, comparing shCbx2 with intact Cbx2 and 162 differentially expressed genes were found (Fig. 5E). All NanoString raw count data can be found in Supplementary Table S3. Notably, *Ifitm1* and *Cxcl13* were downregulated in the setting of *Cbx2* knockdown, whereas *Dusp5* and *Cdh2* were associated with intact *Cbx2* (Fig. 5E). NanoString transcriptional profiles were scored with a significant increase in immune cell adhesion and migration score, as well as myeloid compartment score in the setting of knockdown of *Cbx2* (Fig. 5F; Supplementary Fig. S5G). Of note, there were trends toward a decrease in cell proliferation score and cytokine and chemokine signaling score in the setting of shCbx2. These data demonstrate that modulation of *Cbx2* impacts transcriptional profiling in various models and that the resulting transcriptional changes aid in remodeling the TIME.

To further confirm CBX2-dependent TIME remodeling, tumors derived from the mice were evaluated using Vectra Polaris mIHC. All immune cells were quantified, and there was no difference in total T cells (CD3^+^) or macrophages (F480^+^) between *Cbx2* knockdown and control (Fig. 5G). However, there was an increase in macrophages when proliferation (Ki67^+^) was considered, highlighting a proliferative or active state (Fig. 5H; ref. 44). In shCbx2 #1, there was an increase in proliferating T cells (CD3^+^/Ki67^+^), a marker of T-cell activation (Fig. 5H). In shCbx2 tumors compared with shCtrl, there was a trend (shCbx2 #1, *P* = 0.0781) and significant (shCbx2#2 *P* = 0.0023) increase in proliferating macrophages F480^+^/Ki67^+^ (Fig. 5H). These data confirm the impact of *Cbx2* modulation on the TIME in an immune-competent HGSC murine model and suggest that loss of *Cbx2* expression leads to an increased T-cell activation and infiltration of macrophages.

## Discussion

The HGSC TME is heterogeneous, including multiple nonmalignant cell types that support tumor progression, such as fibroblasts and immune cells. For instance, some TAMs support tumor progression by promoting chemoresistance and reinforcing immune suppression. In this series of investigations, we observed that an understudied epigenetic reader protein, CBX2, promotes the transcriptional reprogramming of tumor cells to support the differentiation of TAMs. We observed that CBX2 expression delineates a unique HGSC epithelial cell type and modulating CBX2 in tumor cells leads to a differential immune microenvironment. Furthermore, modulation of CBX2 leads to differential macrophage infiltration and polarization in both spheroid cultures and *in vivo* syngeneic murine tumors. Using primary human-derived macrophages co-cultured with CBX2 overexpressing HGSC cells, we observed macrophages exhibiting increased M2-like differentiation and elevated phagocytosis.

Targeting the macrophage compartment to inhibit tumor progression and support immune surveillance of the tumor is an attractive approach to promote durable antitumor responses. Rodriguez-Garcia and colleagues (45) demonstrated the utility of a chimeric antigen receptor T-cell targeting TAMs enhances the antitumor response of traditional chimeric antigen receptor T-cell, highlighting that TAM-mediated immune suppression is a critical target in overcoming immune evasion. Another example is macrophage activity and differentiation are dependent on colony-stimulating factor (CSF) stimulation. Thus, several CSF receptor (CSF1Ri) antagonists have been developed to date (46). In HGSC models, the use of CSF1Ri promoted the maintenance of vascular integrity, thereby reducing malignant ascites accumulation and alleviating chemotherapy resistance (47, 48). Taken in the context of our current and prior findings showing CBX2 is critical for HGSC progression (6), targeting CBX2 in tumor cells provides an opportunity to limit tumor progression and potentially alleviate an immune-suppressed microenvironment.

The biology and function of TAMs remain incompletely understood. When initially described, TAMs were defined as a subset of macrophages that contributed to immunosuppression and promoted tumor growth and progression (49). However, further research has demonstrated that TAMs have different states, ranging on the spectrum from M2-like to M1-like, both of which can promote or inhibit cancer progression, respectively (50). Therefore, finding treatments that tip the balance of TAM polarization to a more M1-like and less M2-like phenotype is important moving forward. In the work described, we observed that indirect co-culture of CBX2 expression in cancer cells led to the increase of CD163- and CD206-expressing TAM, highlighting the contribution of secreted factors from the cancer cells regulating the macrophage phenotype toward tumor promotion. Through RNA-seq and qPCR analysis, modulation of CBX2 led to the differential expression of immune regulatory cytokines, including CXCL1. Although multiple cell types express CXCL1 in the TME, several studies demonstrate that elevated CXCL1-mediated signaling supports tumor-promoting macrophage differentiation and cancer progression in a macrophage-dependent manner (51–53). Although we predict that CBX2-mediated regulation of TAM differentiation and activity is highly complex, our findings suggest a unique CBX2/CXCL1 or CBX2/CXCL8 axis in tumor cells that aid in remodeling the HGSC TIME.

Interestingly, in our *in vivo* murine model of HGSC, we observed no difference in the total number of macrophages, contrary to our prediction that CXCL1 signaling in shCTRL (CBX2 intact) tumor cells would increase the number of TAMs present. However, in the CBX2 knockdown tumors, we observed an increase in proliferating macrophages, marked by Ki67 expression. Recent reports have indicated that both tissue resident as well as recruited macrophages can undergo proliferation and self-renewal. Taken together, our data would suggest that inhibition of CBX2 increases the proliferation of macrophages and polarizes them away from an M2-like phenotype. Given the limited understanding of proliferating macrophages in the field, understanding the phenotype of these cells and their plasticity in the context of HGSC is an exciting new direction for our research, particularly given their potential role in therapy resistance (54–56).

In this report, various HGSC models and primary macrophages were used to dissect CBX2-dependent TIME remodeling, however there are limitations. We opted to use human-derived monocytes, rather than THP1 cells; however, this leads to the additional limitation of heterogeneity between samples. Additionally, the use of macrophage receptor status is an imperfect approach to determining macrophage activity as tumor promoting or tumor inhibiting. Thus, we attempted to utilize functional assays to further support our findings. We acknowledge that an additional limitation of this work is that the mechanism is not fully elucidated, and further work should assess chromatin profiling of CBX2 genomic occupancy and exploring the necessity of the adaptive immune response when targeting the CBX2–macrophage interplay.

Overall, the work reported is one of only a few studies to interrogate tumor cell–dependent epigenetic mechanisms that directly contribute to the remodeling of the TIME. CBX2 is a new potential target in HGSC and other malignancies, with the hope of limiting tumor progression and resensitizing to therapy. Thus, understanding the impact of CBX2 on the TIME will allow for optimization of CBX2 inhibition and therapeutic approaches.

## Supplementary Material

Supplementary Data Overview of supplementary data

Figure S1 CBX2 binds to promoter regions of cytokine genes and CBX2 is significantly associated with immune signatures.

Figure S2 CBX2 expression associates with epithelial state 6. CBX2 protein expression does not correlate with T cell infiltration. CXCL1, 5, and CXCL8 expression correlation with Macrophage M0_CIBERSORT infiltration.

Figure S3 Modulation of CBX2 enhances monocyte infiltration

Figure S4 M1/M2 macrophages convey differential survival outcomes. M1/M2 gating strategy. CD68 gating of monocytes in culture system.

Figure S5 In vivo modeling with loss of CBX2 expression and Nanostring pathway analysis

Table S1 Antibodies

Table S2 Primers

Table S3 Nanostring

Table S4 TIMERv2
