# Supplementary material for: Tumor-Intrinsic Activity of Chromobox 2 Remodels the Tumor Microenvironment in High-grade Serous Carcinoma
Source: Cancer Res Commun. 2024 Aug 5;4(8):1919–32. doi: 10.1158/2767-9764.CRC-24-0027 (PMC11298703; doi:10.1158/2767-9764.CRC-24-0027)
Supplement: Supplementary Data — Overview of supplementary data [file crc-24-0027_supplementary_data_suppsd.docx]

**SUPPLEMENTAL METHODS**

**Published CUT&RUN data analysis**

The Cbx2 CUT&RUN data generated by Kim et al [1] and available on GEO (Accession number: GSE210367) was downloaded using fasterq-dump from the SRA toolkit (<https://github.com/ncbi/sra-tools/wiki>). The quality of the fastq files was accessed using FastQC (v.0.11.8) [2], FastQ Screen (v.0.13.0) [3] and MultiQC (v.1.8) [4]. Illumina adapters and low-quality reads were filtered out using BBDuk (v.38.87, <http://jgi.doe.gov/data-and-tools/bb-tools>). Bowtie2 (v.2.3.4.3) [5] was used to align the sequencing reads to the mm10 reference murine genome. Samtools (v.1.11) [6] was used to select the mapped reads (samtools view -b - q 30) and sort the bam files. PCR duplicates were removed using Picard MarkDuplicates tool (v.2.21.1) (<http://broadinstitute.github.io/picard/>). Bigwig files were created using deepTools bamCoverage (v.3.2.1) [7] and visualized using IGV (v.2.8.2) [8]. Peaks were called using MACS2 (v2.1.2) [9].

**SUPPLEMENTAL TABLES**

TABLE S1: Antibody List

TABLE S2: qPCR Primers

TABLE S3: Nanostring Counts Table

TABLE S4: TIMERv2 Analysis of 303 HGSC tumors.

**SUPPLEMENTAL FIGURES**

FIGURE S1. CBX2 binds to promoter regions of cytokine genes and CBX2 is significantly associated with immune signatures.

FIGURE S2. CBX2 expression associates with epithelial state 6. CBX2 protein expression does not correlate with T cell infiltration. CXCL1, 5, and CXCL8 expression correlation with Macrophage M0_CIBERSORT infiltration.

Figure S3. Modulation of CBX2 enhances monocyte infiltration.

Figure S4. M1/M2 macrophages convey differential survival outcomes. M1/M2 gating strategy. CD68 gating of monocytes in culture system.

Figure S5. *In vivo* modeling with loss of CBX2 expression and Nanostring pathway analysis.

**REFERENCES**

1. Kim, J.J., et al., *Cell type-specific role of CBX2 and its disordered region in spermatogenesis.* Genes Dev, 2023. **37**(13-14): p. 640-660.

2. Andrews, S.

*Fastq*. 2010; Available from: <https://www.bioinformatics.babraham.ac.uk/projects/fastqc/>.

3. Wingett, S.W. and S. Andrews, *FastQ Screen: A tool for multi-genome mapping and quality control.* F1000Res, 2018. **7**: p. 1338.

4. Ewels PA, P.A., Fillinger S, Patel H, Alneberg J, Wilm A, Ulysse Garcia M, Di Tommaso P, Nahnsen S, *The nf-core framework for community-curated bioinformatics pipelines.* Nat Biotechnol, 2020. **38**(3): p. 276-278.

5. Langmead, B. and S.L. Salzberg, *Fast gapped-read alignment with Bowtie 2.* Nat Methods, 2012. **9**(4): p. 357-9.

6. Li, H., et al., *The Sequence Alignment/Map format and SAMtools.* Bioinformatics, 2009. **25**(16): p. 2078-9.

7. Ramirez, F., et al., *deepTools2: a next generation web server for deep-sequencing data analysis.* Nucleic Acids Res, 2016. **44**(W1): p. W160-5.

8. Robinson, J.T., et al., *Integrative genomics viewer.* Nat Biotechnol, 2011. **29**(1): p. 24-6.

9. Zhang, Y., et al., *Model-based analysis of ChIP-Seq (MACS).* Genome Biol, 2008. **9**(9): p. R137.
