# Supplementary material for: Tumor-Intrinsic Activity of Chromobox 2 Remodels the Tumor Microenvironment in High-grade Serous Carcinoma
Source: Cancer Res Commun. 2024 Aug 5;4(8):1919–32. doi: 10.1158/2767-9764.CRC-24-0027 (PMC11298703; doi:10.1158/2767-9764.CRC-24-0027)
Supplement: Figure S1 — CBX2 binds to promoter regions of cytokine genes and CBX2 is significantly associated with immune signatures. [file crc-24-0027_figure_s1_supps1.docx]

Supplemental Figure 1, Iwanaga and Yamamoto, 2024


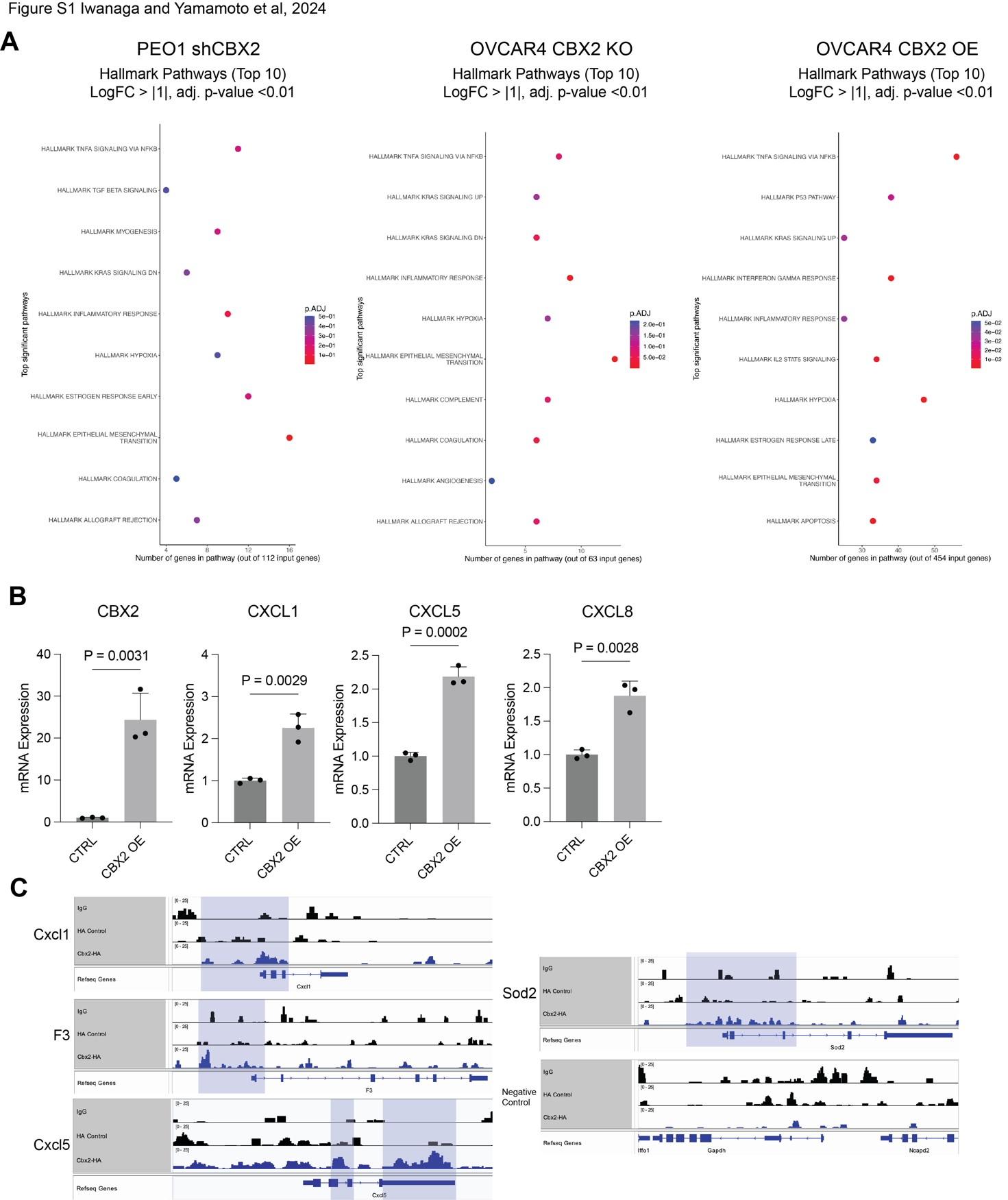


**FIGURE S1. CBX2 binds to promoter regions of cytokine genes and CBX2 is significantly associated with immune signatures. A)** Hallmark pathway analysis of indicated cell line condition with or without CBX2. KO=knockout and OE=overexpression. **B)** OVCAR4 cells transduced with lentivirus control or CBX2 overexpression (OE). qPCR of indicated genes. Internal control, HPRT1. **C)** CUT&RUN of isotype control (IgG), HA control, and Cbx2-HA (data from GSE210367). Blue highlighted portions indicated Cbx2 interaction sites. Error bars, SEM. Statistical test, unpaired t-test.
