# Supplementary material for: Tumor-Intrinsic Activity of Chromobox 2 Remodels the Tumor Microenvironment in High-grade Serous Carcinoma
Source: Cancer Res Commun. 2024 Aug 5;4(8):1919–32. doi: 10.1158/2767-9764.CRC-24-0027 (PMC11298703; doi:10.1158/2767-9764.CRC-24-0027)
Supplement: Figure S2 — CBX2 expression associates with epithelial state 6. CBX2 protein expression does not correlate with T cell infiltration. CXCL1, 5, and CXCL8 expression correlation with Macrophage M0_CIBERSORT infiltration. [file crc-24-0027_figure_s2_supps2.docx]

Supplemental Figure 2, Iwanaga and Yamamoto, 2024


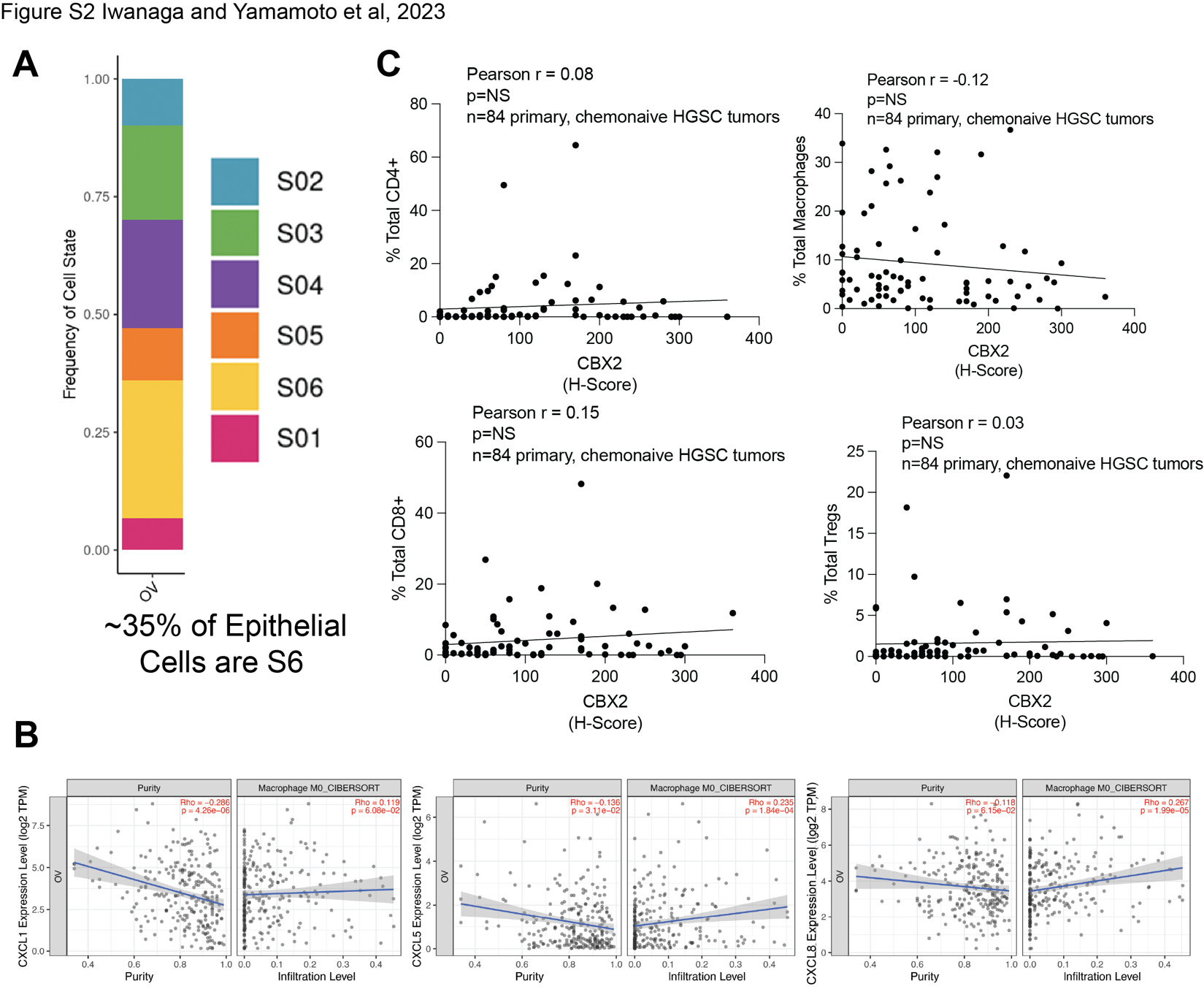


**FIGURE S2. CBX2 expression associates with epithelial state 6. CBX2 protein expression does not correlate with T cell infiltration. CXCL1, 5, and CXCL8 expression correlation with Macrophage M0_CIBERSORT infiltration**. **A)** Carcinoma Ecotyper of epithelial cell states. **B)** TIMERv2 analysis of CXCL1,5, and 8 expression (y-axis) and indicated Macrophage M0_CIBERSORT tumor infiltration. **C)** Tissue microarray of HGSC tumors. analysis of chemo naïve, HGSC tumor expression of CBX2 correlated to CD68+, CD4+, CD8+, and CD4+FOXP3+ (Tregs) cells. Error bars, SEM.
