# Supplementary material for: Tumor-Intrinsic Activity of Chromobox 2 Remodels the Tumor Microenvironment in High-grade Serous Carcinoma
Source: Cancer Res Commun. 2024 Aug 5;4(8):1919–32. doi: 10.1158/2767-9764.CRC-24-0027 (PMC11298703; doi:10.1158/2767-9764.CRC-24-0027)
Supplement: Figure S3 — Modulation of CBX2 enhances monocyte infiltration [file crc-24-0027_figure_s3_supps3.docx]

Supplemental Figure 3, Iwanaga and Yamamoto, 2024


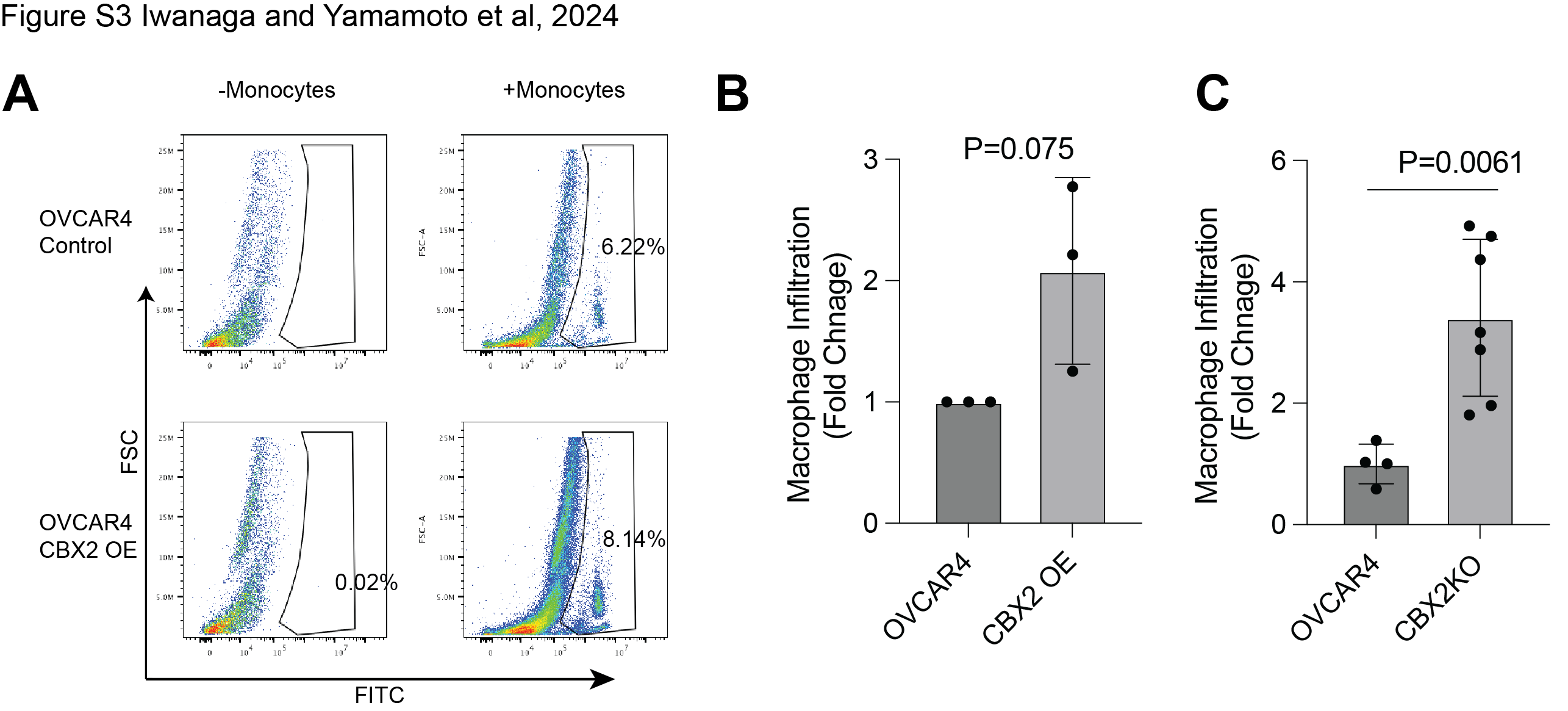


**FIGURE S3. Modulation of CBX2 enhances monocyte infiltration. A)** FITC-tagged monocytes were co-cultured with OVCAR4 cells without (Control) or with CBX2 overexpression (OE). The spheroids were digested and used for flow cytometry against Forward Scatter (FSC) and FITC (monocytes). **B)** Quantification of A. **C)** Same as B, but with CBX2 knockout cells. Statistical test, unpaired t-test. Error bars, SEM.
