# Supplementary material for: Tumor-Intrinsic Activity of Chromobox 2 Remodels the Tumor Microenvironment in High-grade Serous Carcinoma
Source: Cancer Res Commun. 2024 Aug 5;4(8):1919–32. doi: 10.1158/2767-9764.CRC-24-0027 (PMC11298703; doi:10.1158/2767-9764.CRC-24-0027)
Supplement: Figure S4 — M1/M2 macrophages convey differential survival outcomes. M1/M2 gating strategy. CD68 gating of monocytes in culture system. [file crc-24-0027_figure_s4_supps4.docx]

Supplemental Figure 4, Iwanaga and Yamamoto, 2024


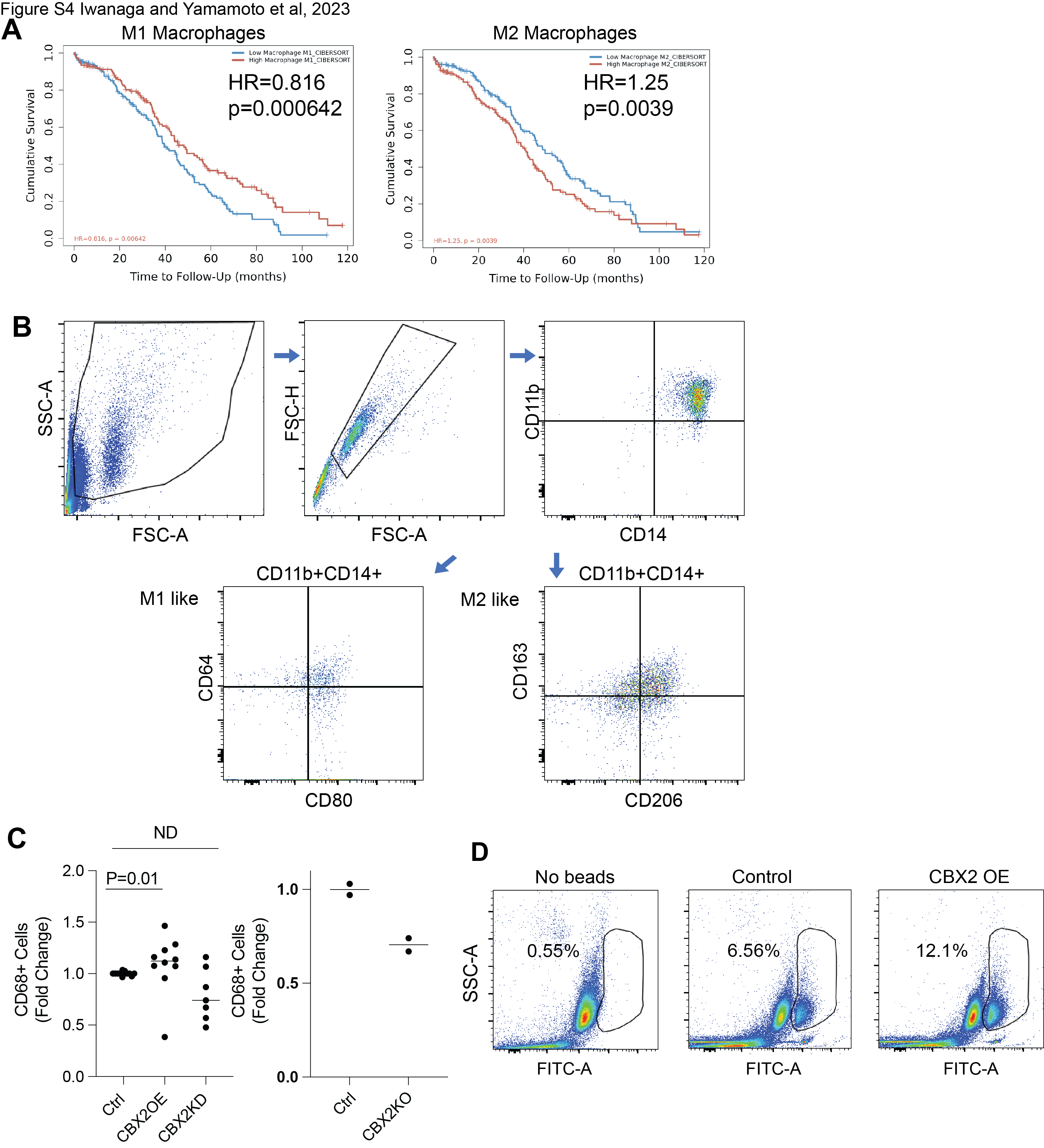


**Figure S4. M1/M2 macrophages convey differential survival outcomes. M1/M2 gating strategy. CD68 gating of monocytes in culture system. A)** Kaplan-Meier analysis of survival outcomes from HGSC tumors with high or low M1_CIBERSORT or high and low M2_CIBERSORT signatures. **B)** Gating strategy of M1/M2 macrophages. **C)** CD68+ positive monocytes in co-culture spheroids with OVCAR4 (Ctrl), OVCAR4 CBX2 overexpression (CBX2OE), OVCAR4 CBX2 knockdown (CBX2KD), and OVCAR4 CBX2 knockout (CBX2KO). **D)** Flow cytometry plots showing percentage of OVCAR4 cells without (No beads) and with FITC beads incubated with OVCAR4 (Control) or OVCAR4 CBX2 OE cell. Statistical test, A – LogRank; C – multicomparison ANOVA.
