# Supplementary material for: Tumor-Intrinsic Activity of Chromobox 2 Remodels the Tumor Microenvironment in High-grade Serous Carcinoma
Source: Cancer Res Commun. 2024 Aug 5;4(8):1919–32. doi: 10.1158/2767-9764.CRC-24-0027 (PMC11298703; doi:10.1158/2767-9764.CRC-24-0027)
Supplement: Figure S5 — In vivo modeling with loss of CBX2 expression and Nanostring pathway analysis [file crc-24-0027_figure_s5_supps5.docx]

Supplemental Figure 5, Iwanaga and Yamamoto, 2024

**
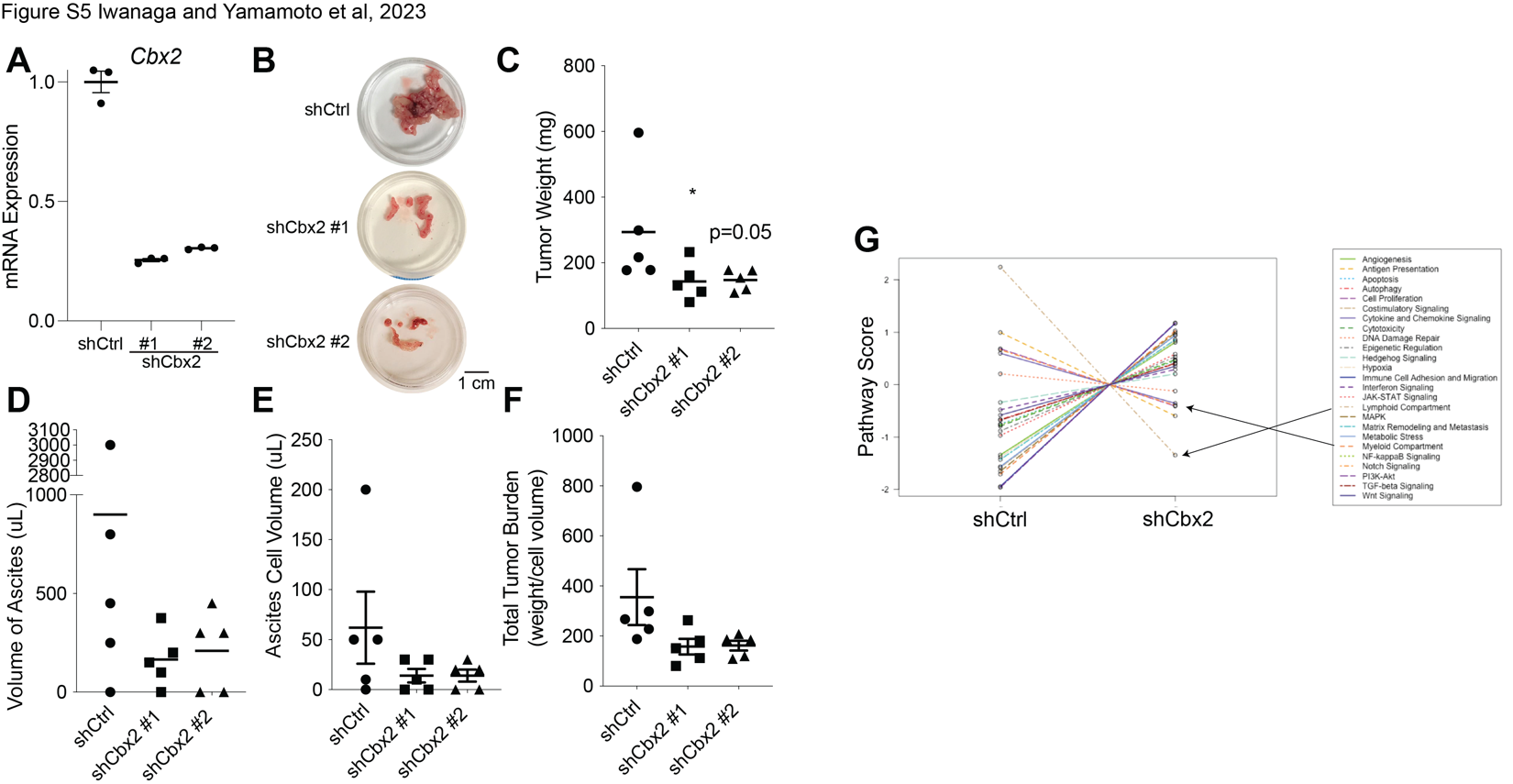
**

**Figure S5. *In vivo* modeling with loss of CBX2 expression and Nanostring pathway analysis. A)** ID8 p53-/- cells were transduced with shRNA against control or Cbx2. qPCR was completed against Cbx2. Internal control, HRPT. B) Tumor burden after 35 days post-tumor cell implantation from a representative mice. **C)** Graph of tumor burden. **D)** Graph of ascites volume. **E)** Graph of ascites cell volume. **F)** Total tumor burden tumor weight + cell volume. **G)** Nanostring pathway analysis summary. Error bars, SEM. Statistical test, multicomparison ANOVA.
