# Supplementary material for: Tumor-Intrinsic Activity of Chromobox 2 Remodels the Tumor Microenvironment in High-grade Serous Carcinoma
Source: Cancer Res Commun. 2024 Aug 5;4(8):1919–32. doi: 10.1158/2767-9764.CRC-24-0027 (PMC11298703; doi:10.1158/2767-9764.CRC-24-0027)
Supplement: Table S1 — Antibodies [file crc-24-0027_table_s1_suppst1.docx]

**TABLE S1: Antibody List**

| ***Human Macrophage Flow antibody lists*** | |  |  |
| --- | --- | --- | --- |
|  | **manufacture** | **catalog#** | **RRID** |
| FC blocker | Biolegend | 422302 | AB_2818986 |
| CD14-FITC | Biolegend | 325603 | AB_830676 |
| CD163-PE | Biolegend | 333606 | AB_1134002 |
| HLADR-PerCP-Cy5.5 | Biolegend | 307630 | AB_893567 |
| CD80-PE-Cy7 | Biolegend | 305218 | AB_2076148 |
| CD11b-APC | Biolegend | 101212 | AB_312795 |
| CD64-Alexa700 | Biolegend | 305040 | AB_2800776 |
| CD206-APC-Cy7 | Biolegend | 321120 | AB_2144930 |
| CD68-BV421 | Biolegend | 333828 | AB_2800882 |

| ***Mouse Multiplexed IHC antibody lists*** | |  |  |
| --- | --- | --- | --- |
|  | **manufacture** | **catalog#** | **RRID** |
| Foxp3 | Invitrogen | 14-5773-82 | AB_467576 |
| ClCasp3 | Cell Signaling Technology | 9661S | AB_2341188 |
| WT1 | Novus | NB110-60011B | AB_962464 |
| CD11c | Cell Signaling Technology | 97585S | [AB_2800282](https://www.antibodyregistry.org/AB_2800282) |
| B220 (CD45R) | BD Pharm | 557390 | AB_396673 |
| CD3 | Cell Signaling Technology | 999440S | AB_2755035 |
| Ki67 | EPREDIA (Thermofisher) | RM-9106-S | AB_2341197 |
| F480 | Cell Signaling Technology | 30325S | AB_2798990 |
|  |  |  |  |
