# Supplementary material for: Tumor-Intrinsic Activity of Chromobox 2 Remodels the Tumor Microenvironment in High-grade Serous Carcinoma
Source: Cancer Res Commun. 2024 Aug 5;4(8):1919–32. doi: 10.1158/2767-9764.CRC-24-0027 (PMC11298703; doi:10.1158/2767-9764.CRC-24-0027)
Supplement: Table S2 — Primers [file crc-24-0027_table_s2_suppst2.docx]

| Iwanaga, Yamamoto, et al 2024, Sup. Table 2 | | |
| --- | --- | --- |
| **Primers** |  |  |
| **Gene** | **Direction** | **Sequence (5’-3’)** |
| human CBX2 | Forward | CGGCTGGTCCTCCAAACATAA |
|  | Reverse | CAGAACCGGAAGAGAGGCAA |
|  |  |  |
| human HPRT1 | Forward | TGACACTGGCAAAACAATGCA |
|  | Reverse | GGTCCTTTTCACCAGCAAGCT |
|  |  |  |
| human GAPDH | Forward | GTCTCCTCTGACTTCAACAGCG |
|  | Reverse | ACCACCCTGTTGCTGTAGCCAA |
|  |  |  |
| human CXCL1 | Forward | AGCTTGCCTCAATCCTGCATCC |
|  | Reverse | TCCTTCAGGAACAGCCACCAGT |
|  |  |  |
| human CXCL8 | Forward | GAGAGTGATTGAGAGTGGACCAC |
|  | Reverse | CACAACCCTCTGCACCCAGTTT |
|  |  |  |
| Mouse CBX2 | Forward | AAGCTGGAGTACCTGGTCAAG |
|  | Reverse | ACCTCCTTCTCATGTTCCTTCTTC |
|  |  |  |
| Mouse GAPDH | Forward | TGCACCACCAACTGCTTAG |
|  | Reverse | GGATGCAGGGATGATGTTC |

Table S2. qPCR Primer Sequences.
