# Supplementary material for: Tumor-Intrinsic Activity of Chromobox 2 Remodels the Tumor Microenvironment in High-grade Serous Carcinoma
Source: Cancer Res Commun. 2024 Aug 5;4(8):1919–32. doi: 10.1158/2767-9764.CRC-24-0027 (PMC11298703; doi:10.1158/2767-9764.CRC-24-0027)
Supplement: Table S3 — Nanostring [file crc-24-0027_table_s3_suppst3.docx]

| **Table S3, Brubaker et al , 2024** | |  |  |  |  |  |  |  |  |  |  |
| --- | --- | --- | --- | --- | --- | --- | --- | --- | --- | --- | --- |
|  |  | 20210520_30102598530422-01_Sample06_06.RCC | 20210520_30102598530422-01_Sample07_07.RCC | 20210520_30102598530422-01_Sample09_09.RCC | 20210520_30102598530422-01_Sample01_01.RCC | 20210520_30102598530422-01_Sample04_04.RCC | 20210520_30102598530422-01_Sample05_05.RCC | 20210520_30102598530422-01_Sample03_03.RCC | 20210520_30102598530422-01_Sample10_10.RCC | 20210520_30102598530422-01_Sample11_11.RCC | 20210520_30102598530422-01_Sample12_12.RCC |
| Probe Name | Accession # | Control/1051 | Control/1052 | Control/1055 | shCBX2#1/1044 | shCBX2#1/1048 | shCBX2#1/1049 | shCBX2#2/1046 | shCBX2#2/1056 | shCBX2#2/1057 | shCBX2#2/1058 |
| Dab2 | NM_023118.5 | 1960.5 | 1966.35 | 2103.82 | 3726.85 | 3758.89 | 3200.9 | 3126.42 | 3338.39 | 2979.08 | 2980.78 |
| Nfkbia | NM_010907.2 | 1849.37 | 2059.8 | 2061.72 | 2847.1 | 2887.47 | 2444.32 | 2421.56 | 2554.36 | 2276.92 | 2469.45 |
| Ifitm1 | NM_001112715.1 | 402.25 | 469.21 | 491.97 | 201.19 | 228.52 | 261.3 | 179 | 209.15 | 270.14 | 237.83 |
| Ms4a1 | NM_007641.5 | 237.97 | 224.28 | 70.16 | 37.96 | 37.58 | 35.63 | 42.66 | 59.43 | 27.87 | 28.74 |
| Cd19 | NM_009844.2 | 230.72 | 276.41 | 92.08 | 19.93 | 45.7 | 48.7 | 32.46 | 72 | 35.38 | 22.79 |
| Mtor | NM_020009.2 | 366.01 | 399.37 | 426.2 | 524.81 | 644.93 | 618.8 | 518.44 | 665.16 | 508.13 | 506.38 |
| Eno1 | NM_023119.2 | 9344.69 | 11309.21 | 10152.52 | 14404.4 | 14613.05 | 14936.74 | 14351.32 | 14048.43 | 10980.47 | 15373.63 |
| Col4a5 | NM_001163155.1 | 535.12 | 632.5 | 739.27 | 839.89 | 980.09 | 865.85 | 854.18 | 876.6 | 752.54 | 798.71 |
| Cdh2 | NM_007664.5 | 186.02 | 144.6 | 167.5 | 573.22 | 415.4 | 420.45 | 337.59 | 469.73 | 410.57 | 397.37 |
| Tnfrsf1b | NM_011610.3 | 369.63 | 502.65 | 513.9 | 544.74 | 619.54 | 655.62 | 598.2 | 576.02 | 543.5 | 610.43 |
| Chuk | NM_001162410.1 | 739.26 | 903.99 | 875.2 | 920.56 | 1027.83 | 964.43 | 961.76 | 1009.17 | 944.43 | 985.01 |
| Dtx4 | NM_172442.3 | 527.87 | 605.94 | 747.17 | 415.68 | 437.74 | 509.53 | 499.89 | 432.01 | 517.77 | 453.86 |
| Esr1 | NM_007956.5 | 300.78 | 311.82 | 354.29 | 415.68 | 562.67 | 554.66 | 385.82 | 558.87 | 529.57 | 447.91 |
| Cd79b | NM_008339.2 | 223.47 | 311.82 | 89.45 | 52.2 | 66.02 | 71.26 | 48.23 | 61.72 | 47.17 | 42.61 |
| Prkca | NM_011101.3 | 407.08 | 569.54 | 516.53 | 726.01 | 855.17 | 629.49 | 870.87 | 686.88 | 603.53 | 796.73 |
| Il10 | NM_010548.1 | 15 | 17.71 | 15 | 27.52 | 47.74 | 46.32 | 25.97 | 32 | 45.02 | 48.56 |
| Tlr2 | NM_011905.2 | 152.2 | 226.24 | 193.81 | 242 | 303.68 | 275.55 | 257.83 | 288.01 | 279.79 | 269.54 |
| Lif | NM_008501.2 | 102.68 | 123.94 | 139.44 | 145.2 | 168.6 | 188.85 | 156.74 | 201.15 | 160.8 | 150.62 |
| Map3k12 | NM_001163643.1 | 237.97 | 299.03 | 253.44 | 300.84 | 290.47 | 348 | 339.45 | 361.15 | 358.05 | 342.87 |
| Rela | NM_009045.4 | 1326.33 | 1502.06 | 1561.86 | 1669.35 | 1868.78 | 1792.27 | 1650.86 | 1564.62 | 1628.36 | 1703.45 |
| Olfml2b | NM_177068.4 | 1017.09 | 1080.07 | 1183.89 | 1879.08 | 1873.86 | 1696.06 | 1693.52 | 1635.48 | 1623 | 1495.35 |
| Blk | NM_007549.2 | 36.24 | 42.3 | 15 | 15 | 15 | 15 | 15 | 15 | 15 | 15 |
| Cd5 | NM_007650.3 | 62.81 | 57.05 | 56.13 | 37.96 | 47.74 | 38.01 | 38.03 | 49.14 | 36.45 | 29.73 |
| H2-Ob | NM_010389.3 | 100.26 | 145.58 | 77.17 | 54.09 | 63.99 | 45.13 | 49.15 | 58.29 | 43.95 | 47.57 |
| Tgfb3 | NM_009368.2 | 1188.62 | 1507.96 | 1679.37 | 1671.24 | 1833.23 | 1934.79 | 1803.88 | 1985.2 | 1617.64 | 1829.3 |
| Aldoc | NM_009657.3 | 150.99 | 140.66 | 121.9 | 270.47 | 261.02 | 206.66 | 193.84 | 206.86 | 175.81 | 175.4 |
| Sfrp4 | NM_016687.3 | 426.41 | 708.24 | 542.84 | 733.6 | 774.93 | 890.79 | 1078.62 | 941.74 | 770.77 | 863.12 |
| Vegfb | NM_011697.3 | 554.45 | 519.38 | 543.71 | 627.31 | 739.39 | 706.69 | 637.16 | 642.31 | 561.73 | 625.29 |
| Nf1 | NM_010897.2 | 434.86 | 579.38 | 604.22 | 619.72 | 688.61 | 644.93 | 681.67 | 674.31 | 662.49 | 694.66 |
| Dusp5 | NM_001085390.1 | 312.86 | 340.35 | 312.2 | 496.34 | 535.24 | 466.77 | 498.97 | 480.01 | 442.73 | 491.51 |
| Flt1 | NM_010228.3 | 774.3 | 1054.49 | 932.2 | 2082.18 | 1526.51 | 1338.56 | 1458.87 | 1587.48 | 1247.8 | 1740.11 |
| Hdac5 | NM_010412.3 | 574.98 | 615.77 | 554.24 | 683.3 | 824.7 | 619.99 | 719.7 | 696.02 | 654.99 | 761.05 |
| Trp53 | NM_011640.1 | 253.67 | 218.37 | 257.83 | 206.89 | 184.85 | 228.04 | 200.33 | 226.29 | 191.89 | 217.02 |
| Met | NM_008591.2 | 439.69 | 470.19 | 523.54 | 827.56 | 933.37 | 757.76 | 688.17 | 761.17 | 615.33 | 599.53 |
| Rictor | NM_030168.3 | 572.57 | 590.2 | 630.53 | 823.76 | 795.25 | 766.08 | 720.63 | 781.74 | 726.81 | 871.05 |
| Itgav | NM_008402.3 | 1360.15 | 1765.68 | 1727.6 | 1988.22 | 2283.16 | 2063.07 | 2005.14 | 2022.92 | 1736.64 | 1930.37 |
| Polr2a | NM_009089.2 | 2645.41 | 2756.23 | 3225.44 | 3779.04 | 4260.62 | 3832.77 | 3594.78 | 3544.11 | 3113.08 | 4026.24 |
| Fzd9 | NM_010246.1 | 24.16 | 18.69 | 15 | 15 | 15 | 15 | 15 | 15 | 15 | 15 |
| Tnfaip6 | NM_009398.2 | 62.81 | 136.73 | 63.14 | 243.9 | 226.49 | 153.22 | 194.76 | 172.58 | 180.1 | 180.35 |
| Pgpep1 | NM_023217.4 | 430.03 | 454.45 | 509.51 | 543.8 | 502.74 | 520.22 | 474.85 | 513.16 | 516.7 | 517.28 |
| Mxi1 | NM_001008542.2 | 1165.67 | 1080.07 | 921.68 | 1862 | 1890.11 | 1408.63 | 1403.23 | 1578.33 | 1107.37 | 1529.04 |
| Sell | XM_006496716.1 | 223.47 | 383.63 | 70.16 | 78.77 | 53.83 | 73.64 | 49.15 | 84.57 | 54.67 | 30.72 |
| Ndufa4l2 | NM_001098789.1 | 269.37 | 265.59 | 183.28 | 412.83 | 302.66 | 412.14 | 321.82 | 342.87 | 248.7 | 344.85 |
| Ikzf1 | NM_009578.2 | 382.92 | 262.64 | 170.13 | 161.34 | 193.99 | 181.72 | 134.48 | 174.86 | 168.3 | 163.51 |
| Fgf9 | NM_013518.4 | 25.37 | 18.69 | 15 | 15 | 15 | 15 | 15 | 15 | 15 | 15 |
| Ltb | NM_008518.2 | 258.5 | 364.94 | 235.02 | 154.69 | 166.57 | 193.6 | 128.92 | 203.43 | 206.9 | 126.84 |
| Ikzf3 | NM_011771.1 | 149.79 | 178.04 | 100.85 | 78.77 | 93.44 | 84.33 | 51.01 | 100.57 | 68.61 | 57.48 |
| Plod2 | NM_001142916.1 | 806.91 | 1175.48 | 885.73 | 1596.27 | 1698.15 | 1601.04 | 1471.86 | 1377.19 | 1335.71 | 1515.17 |
| Pik3r2 | NM_008841.2 | 957.9 | 1065.31 | 1132.15 | 1380.84 | 1389.4 | 1279.17 | 1179.71 | 1298.33 | 1163.12 | 1156.44 |
| Stat3 | NM_011486.4 | 1921.85 | 2462.12 | 2563.34 | 2644 | 2674.18 | 2962.17 | 2866.74 | 2906.38 | 2866.52 | 3019.43 |
| Ngfr | NM_033217.3 | 36.24 | 63.94 | 50.86 | 21.83 | 34.53 | 22.57 | 40.81 | 15 | 26.8 | 28.74 |
| Ccl19 | NM_011888.2 | 147.37 | 283.3 | 130.67 | 111.99 | 85.31 | 146.09 | 80.69 | 82.29 | 113.63 | 92.16 |
| G6pdx | NM_008062.2 | 956.7 | 975.8 | 1011.13 | 1041.09 | 1179.16 | 1191.28 | 1306.77 | 1059.46 | 1069.85 | 1041.49 |
| Lyz1 | NM_013590.3 | 9505.35 | 10089.46 | 11064.56 | 13533.19 | 11920.59 | 14966.43 | 11030.13 | 14918.17 | 12421.23 | 14299.43 |
| Icam1 | NM_010493.2 | 469.89 | 544.95 | 640.18 | 710.82 | 811.5 | 839.72 | 782.77 | 920.03 | 830.8 | 724.39 |
| Gli1 | NM_010296.2 | 18.12 | 38.36 | 29.82 | 15 | 15 | 29.69 | 17.62 | 15 | 15 | 15 |
| Ier3 | NM_133662.2 | 571.36 | 731.85 | 749.8 | 1692.12 | 1698.15 | 1142.58 | 1152.82 | 1186.32 | 965.87 | 1153.47 |
| Got1 | NM_010324.2 | 1131.85 | 1150.89 | 1293.51 | 1454.87 | 1552.92 | 1446.64 | 1522.87 | 1554.33 | 1327.13 | 1526.07 |
| Irf5 | NM_001252382.1 | 989.31 | 914.81 | 974.3 | 1076.2 | 1480.81 | 1179.4 | 1088.82 | 1306.33 | 1259.6 | 1140.59 |
| Cxcr5 | NM_007551.2 | 53.15 | 63.94 | 21.92 | 24.67 | 29.45 | 23.75 | 20.4 | 21.71 | 24.66 | 16.85 |
| Cdk2 | NM_016756.4 | 153.41 | 135.75 | 123.65 | 104.39 | 100.55 | 119.96 | 115.93 | 109.72 | 120.06 | 106.03 |
| Pkm | NM_001253883.1 | 560.49 | 589.22 | 643.69 | 798.14 | 710.95 | 734.01 | 677.04 | 782.88 | 676.43 | 774.92 |
| Bad | NM_007522.3 | 361.18 | 460.36 | 504.25 | 428.96 | 568.76 | 552.29 | 530.5 | 573.73 | 489.9 | 556.91 |
| Tnfaip3 | NM_001166402.1 | 312.86 | 368.87 | 349.03 | 516.27 | 544.38 | 463.21 | 396.95 | 440.01 | 374.13 | 456.83 |
| Pfkfb3 | NM_133232.2 | 433.65 | 522.33 | 478.82 | 576.06 | 598.21 | 562.98 | 554.61 | 554.3 | 507.05 | 556.91 |
| Tnfsf4 | NM_009452.2 | 25.37 | 34.43 | 28.06 | 34.17 | 35.55 | 38.01 | 36.17 | 53.72 | 41.81 | 40.63 |
| Csf1 | NM_001113529.1 | 757.38 | 833.16 | 938.34 | 952.83 | 902.91 | 1095.08 | 1009.06 | 1040.03 | 1067.71 | 1010.77 |
| Icosl | NM_015790.3 | 199.31 | 250.83 | 194.68 | 164.18 | 182.82 | 140.15 | 178.07 | 163.43 | 210.11 | 167.47 |
| Dpp4 | NM_001159543.1 | 179.98 | 255.75 | 205.21 | 158.49 | 161.49 | 169.84 | 172.51 | 189.72 | 168.3 | 176.39 |
| Il11ra1 | NM_010549.3 | 103.88 | 88.53 | 106.11 | 74.97 | 59.92 | 74.83 | 83.47 | 64 | 63.25 | 53.51 |
| Slc2a1 | NM_011400.3 | 648.67 | 696.44 | 454.26 | 1738.63 | 1291.9 | 1274.42 | 848.61 | 1067.46 | 801.85 | 1170.31 |
| Syk | NM_001198977.1 | 410.7 | 464.29 | 327.1 | 323.62 | 317.9 | 375.32 | 270.81 | 336.01 | 336.61 | 290.35 |
| Cxcl16 | NM_023158.6 | 1627.11 | 1922.08 | 1915.27 | 2129.63 | 2647.78 | 2139.08 | 2273.17 | 2401.22 | 2480.6 | 2591.34 |
| Gusb | NM_010368.1 | 739.26 | 872.51 | 879.59 | 811.42 | 998.38 | 984.62 | 1032.25 | 1125.75 | 958.37 | 1107.88 |
| Nras | NM_010937.2 | 841.94 | 926.61 | 998.85 | 1011.67 | 1006.5 | 1016.69 | 1049.87 | 1144.04 | 1022.69 | 983.02 |
| Itgb3 | NM_016780.2 | 190.86 | 237.06 | 208.72 | 379.61 | 276.25 | 302.87 | 356.14 | 321.15 | 270.14 | 375.57 |
| Nfil3 | NM_017373.3 | 431.24 | 511.51 | 414.8 | 676.66 | 696.73 | 562.98 | 498.04 | 562.3 | 523.13 | 549.98 |
| Jak2 | NM_008413.3 | 460.23 | 517.41 | 491.1 | 553.29 | 674.39 | 553.48 | 546.27 | 546.3 | 607.82 | 588.63 |
| Havcr2 | NM_134250.2 | 109.92 | 140.66 | 142.94 | 239.16 | 219.38 | 180.53 | 181.78 | 195.43 | 137.22 | 219 |
| Nfkb1 | NM_008689.2 | 481.97 | 516.42 | 511.27 | 569.42 | 600.24 | 548.73 | 497.11 | 542.87 | 560.65 | 577.72 |
| Itgb2 | NM_008404.4 | 524.25 | 570.53 | 649.82 | 727.91 | 640.87 | 853.97 | 676.11 | 820.6 | 716.09 | 828.44 |
| Nrde2 | NM_183155.3 | 66.44 | 64.92 | 85.94 | 98.7 | 102.58 | 92.64 | 85.33 | 80 | 77.18 | 85.22 |
| P4ha1 | NM_011030.1 | 647.46 | 809.56 | 523.54 | 1009.77 | 1003.45 | 1095.08 | 792.04 | 859.45 | 755.76 | 1217.88 |
| F2rl1 | NM_007974.4 | 50.73 | 75.74 | 49.99 | 36.06 | 36.56 | 48.7 | 48.23 | 32 | 25.73 | 23.78 |
| Mgmt | NM_008598.2 | 353.93 | 445.6 | 506 | 583.65 | 484.46 | 624.74 | 516.59 | 532.59 | 493.12 | 511.33 |
| Ifnar1 | NM_010508.1 | 1134.26 | 1352.54 | 1425.05 | 1433.04 | 1450.34 | 1513.15 | 1558.11 | 1578.33 | 1392.52 | 1447.78 |
| Btla | NM_001037719.2 | 128.04 | 177.06 | 75.42 | 66.43 | 71.09 | 71.26 | 48.23 | 86.86 | 77.18 | 46.57 |
| Tgfb2 | NM_009367.4 | 484.39 | 506.59 | 592.82 | 604.53 | 679.46 | 662.75 | 652.92 | 794.31 | 596.03 | 618.35 |
| H2-DMb2 | NM_010388.4 | 293.53 | 334.45 | 223.62 | 194.55 | 184.85 | 192.41 | 115 | 205.72 | 218.69 | 132.79 |
| Flnb | NM_134080.1 | 2586.22 | 3454.63 | 3400.84 | 4055.21 | 4001.63 | 4202.15 | 4815.3 | 4229.84 | 3566.53 | 4645.58 |
| Tlr8 | NM_133212.2 | 142.54 | 187.88 | 172.76 | 352.09 | 287.43 | 247.05 | 273.6 | 301.72 | 184.38 | 321.07 |
| Ppl | NM_008909.2 | 294.74 | 303.95 | 392 | 313.18 | 537.27 | 433.52 | 508.24 | 558.87 | 332.32 | 387.46 |
| Pik3cd | XM_003945690.1 | 349.1 | 458.39 | 417.43 | 272.37 | 233.6 | 339.69 | 284.73 | 318.87 | 319.46 | 257.65 |
| Palmd | NM_023245.3 | 122 | 112.14 | 130.67 | 109.14 | 80.24 | 98.58 | 93.67 | 84.57 | 86.83 | 94.14 |
| Ppargc1b | NM_133249.2 | 111.13 | 103.28 | 116.64 | 82.57 | 96.49 | 103.33 | 67.7 | 100.57 | 107.2 | 71.35 |
| Cd40lg | NM_011616.2 | 28.99 | 27.54 | 21.05 | 15 | 15 | 23.75 | 15 | 15 | 16.08 | 15 |
| Cd2 | NM_013486.2 | 224.68 | 242.97 | 163.11 | 121.48 | 170.63 | 130.65 | 71.41 | 139.43 | 118.99 | 97.11 |
| Mre11a | NM_018736.2 | 445.73 | 432.81 | 424.45 | 489.7 | 530.16 | 508.34 | 511.02 | 541.73 | 422.37 | 445.93 |
| Ccl21a | NM_011124.4 | 194.48 | 904.97 | 60.51 | 19.93 | 20.31 | 179.35 | 139.12 | 36.57 | 220.83 | 33.69 |
| Tnfrsf11b | NM_008764.3 | 42.28 | 35.41 | 78.93 | 26.57 | 33.52 | 39.19 | 28.75 | 19.43 | 17.15 | 21.8 |
| Cd244 | NM_018729.2 | 135.29 | 168.21 | 171.88 | 199.3 | 186.88 | 224.48 | 223.51 | 216.01 | 177.95 | 218.01 |
| Zeb2 | NM_015753.4 | 358.76 | 393.47 | 463.03 | 660.53 | 677.43 | 545.16 | 550.9 | 633.16 | 432.01 | 530.16 |
| Cd36 | NM_007643.3 | 660.75 | 525.28 | 344.64 | 326.47 | 212.27 | 561.79 | 219.8 | 332.58 | 249.78 | 157.56 |
| Gm5150 | NM_001081687.1 | 30.2 | 26.56 | 21.05 | 19.93 | 15 | 27.32 | 15.77 | 27.43 | 17.15 | 17.84 |
| Il1rn | NM_001039701.3 | 754.97 | 757.42 | 1236.51 | 594.09 | 716.03 | 699.57 | 714.13 | 748.59 | 710.73 | 666.91 |
| Gimap6 | NM_153175.3 | 237.97 | 321.66 | 217.49 | 213.53 | 255.94 | 195.97 | 161.38 | 224.01 | 184.38 | 162.52 |
| C1qb | NM_009777.2 | 4601.08 | 5654.11 | 5965.06 | 6253.17 | 6619.95 | 7241.52 | 6616.41 | 7807.1 | 6210.08 | 7605.56 |
| 4930578C19Rik | NM_175228.4 | 15 | 28.53 | 21.05 | 15 | 21.33 | 17.82 | 15 | 15 | 20.37 | 15 |
| Gpc4 | NM_008150.2 | 1449.54 | 1759.78 | 1842.48 | 1693.07 | 1938.86 | 1801.77 | 1842.84 | 1890.34 | 1749.5 | 1805.51 |
| Tfrc | NM_011638.4 | 3123.76 | 1089.9 | 1022.53 | 1299.22 | 999.39 | 859.91 | 1015.55 | 925.74 | 879.04 | 834.38 |
| Hk2 | NM_013820.3 | 572.57 | 655.12 | 548.97 | 803.83 | 679.46 | 757.76 | 565.74 | 626.3 | 656.06 | 758.08 |
| Cxcr2 | NM_009909.3 | 20.54 | 20.66 | 15 | 16.13 | 15 | 15 | 15 | 15 | 15 | 15 |
| Cd79a | NM_007655.3 | 26.57 | 28.53 | 22.8 | 24.67 | 18.28 | 19 | 21.33 | 25.14 | 16.08 | 19.82 |
| Ctla4 | NM_009843.3 | 48.32 | 64.92 | 67.53 | 121.48 | 105.63 | 73.64 | 58.43 | 109.72 | 83.62 | 72.34 |
| Aldoa | NM_001177307.1 | 8697.23 | 9570.08 | 7980.3 | 12903.04 | 10983.15 | 11539.87 | 9840.21 | 8914.56 | 9070.17 | 10098.79 |
| Slc1a5 | NM_009201.2 | 491.64 | 435.76 | 346.4 | 339.75 | 272.19 | 323.06 | 345.01 | 316.58 | 329.1 | 326.02 |
| Hmga1 | NM_001166545.1 | 108.72 | 129.84 | 130.67 | 233.46 | 174.69 | 188.85 | 197.55 | 178.29 | 165.09 | 266.57 |
| Col6a3 | XM_897036.2 | 757.38 | 1216.79 | 1036.56 | 1269.8 | 1311.19 | 1559.47 | 1389.32 | 1408.04 | 1282.11 | 1350.67 |
| Bcat1 | NM_001024468.3 | 82.14 | 119.02 | 133.3 | 112.93 | 60.94 | 77.2 | 99.24 | 98.29 | 76.11 | 85.22 |
| Ldha | NM_010699.2 | 8983.51 | 10520.3 | 9041.42 | 16156.32 | 11661.6 | 14264.49 | 12095.76 | 12696.39 | 9972.79 | 13125.16 |
| Mmrn2 | NM_153127.3 | 91.8 | 127.88 | 110.5 | 77.82 | 91.41 | 92.64 | 71.41 | 113.15 | 96.48 | 73.33 |
| Il16 | NM_010551.3 | 680.08 | 662.99 | 842.75 | 532.41 | 565.71 | 623.55 | 589.86 | 566.87 | 566.01 | 537.1 |
| Ccr9 | NM_009913.6 | 15 | 15 | 18.42 | 15 | 15 | 15 | 15 | 15 | 15 | 15 |
| Fosl1 | NM_010235.2 | 15 | 31.48 | 19.29 | 63.59 | 22.34 | 40.38 | 33.39 | 32 | 38.59 | 36.67 |
| Bcl2l1 | NM_009743.5 | 1135.47 | 951.2 | 1024.28 | 919.61 | 958.77 | 908.6 | 903.33 | 944.03 | 991.6 | 888.88 |
| Twf1 | NM_008971.4 | 944.62 | 1073.18 | 1062.87 | 994.59 | 1129.39 | 1158.02 | 1253.91 | 1235.47 | 1158.83 | 1087.07 |
| Psmb8 | NM_010724.2 | 1613.82 | 1739.12 | 1625.88 | 2435.22 | 1838.31 | 2073.76 | 1656.42 | 1917.77 | 2004.63 | 2059.2 |
| Egfr | NM_207655.2 | 338.23 | 370.84 | 356.04 | 462.18 | 496.65 | 592.67 | 540.7 | 534.87 | 479.18 | 484.58 |
| Ctss | NM_021281.3 | 5937.07 | 7453.24 | 7647.06 | 8346.73 | 9007.73 | 8837.81 | 8297.87 | 9193.43 | 8262.95 | 10276.17 |
| Reln | NM_011261.2 | 39.86 | 35.41 | 16.66 | 18.98 | 18.28 | 33.26 | 17.62 | 15 | 19.3 | 16.85 |
| Vegfa | NM_001025250.3 | 2806.06 | 2628.35 | 1637.28 | 5083.01 | 4149.91 | 3404 | 3001.22 | 3088.09 | 2488.11 | 3755.71 |
| Cebpb | NM_009883.4 | 648.67 | 895.14 | 672.63 | 1434.94 | 1084.71 | 1152.09 | 1077.69 | 930.31 | 911.2 | 1247.61 |
| Ly6g | XM_909927.2 | 19.33 | 15 | 15 | 15 | 15 | 15 | 15 | 15 | 15 | 15 |
| Casp9 | NM_015733.4 | 161.87 | 193.78 | 209.59 | 181.27 | 242.74 | 236.36 | 207.75 | 221.72 | 210.11 | 229.9 |
| Pdcd1 | NM_008798.1 | 79.72 | 55.09 | 64.02 | 127.17 | 104.61 | 108.08 | 54.72 | 106.29 | 110.42 | 96.12 |
| H2-M3 | NM_013819.2 | 300.78 | 300.02 | 315.7 | 391.95 | 376.8 | 415.7 | 294.93 | 460.59 | 422.37 | 396.38 |
| Tnfrsf9 | NM_001077508.1 | 15 | 15.74 | 15 | 16.13 | 21.33 | 33.26 | 17.62 | 27.43 | 25.73 | 23.78 |
| Prdm1 | NM_007548.3 | 45.9 | 48.2 | 51.74 | 72.13 | 80.24 | 70.08 | 43.59 | 57.14 | 62.18 | 81.26 |
| Snca | NM_009221.2 | 940.99 | 48.2 | 26.31 | 28.47 | 45.7 | 27.32 | 15 | 20.57 | 27.87 | 30.72 |
| Arnt2 | NM_007488.2 | 32.61 | 15 | 21.05 | 15.18 | 15 | 19 | 16.69 | 15 | 19.3 | 16.85 |
| Loxl2 | NM_033325.2 | 1032.8 | 1472.55 | 946.24 | 2030.93 | 1717.45 | 1634.3 | 1934.65 | 1574.91 | 1230.65 | 1506.25 |
| Pdzk1ip1 | NM_026018.3 | 271.79 | 39.35 | 15 | 15 | 15 | 27.32 | 22.26 | 17.14 | 19.3 | 15 |
| Cep55 | NM_028760.1 | 148.58 | 249.85 | 217.49 | 413.78 | 261.02 | 271.99 | 305.13 | 325.72 | 221.9 | 250.71 |
| Itgb8 | NM_177290.3 | 173.94 | 177.06 | 209.59 | 185.06 | 254.93 | 205.48 | 217.02 | 210.29 | 245.49 | 242.78 |
| Rpl23 | NM_022891.3 | 606.39 | 647.25 | 719.1 | 753.53 | 703.84 | 870.6 | 742.89 | 782.88 | 652.85 | 831.41 |
| Cd14 | NM_009841.3 | 155.83 | 164.27 | 182.41 | 567.52 | 555.56 | 262.49 | 341.3 | 264.01 | 203.68 | 534.12 |
| Ptpn11 | NM_011202.3 | 889.05 | 935.47 | 1049.72 | 912.97 | 1207.6 | 1237.6 | 1133.34 | 1104.03 | 992.67 | 1072.21 |
| Pparg | NM_011146.3 | 15 | 34.43 | 25.43 | 15 | 15 | 23.75 | 15.77 | 15 | 21.44 | 15.86 |
| Ccne1 | NM_007633.2 | 287.49 | 60.99 | 62.26 | 85.41 | 59.92 | 60.57 | 67.7 | 73.15 | 64.32 | 57.48 |
| Itga4 | NM_010576.3 | 614.85 | 355.1 | 309.57 | 358.73 | 378.83 | 313.56 | 265.25 | 346.3 | 345.18 | 304.22 |
| Pvr | NM_027514.2 | 363.59 | 476.09 | 434.09 | 489.7 | 478.37 | 496.47 | 559.25 | 438.87 | 440.59 | 508.36 |
| Wnt5b | NM_009525.3 | 86.97 | 80.66 | 114.88 | 103.44 | 137.11 | 116.4 | 109.44 | 121.15 | 140.43 | 103.06 |
| Stk11ip | NM_027886.3 | 140.12 | 131.81 | 145.57 | 125.27 | 114.77 | 148.46 | 123.35 | 129.15 | 138.29 | 128.82 |
| Klrg1 | NM_016970.1 | 15 | 24.59 | 15 | 15 | 15 | 15 | 15 | 15 | 18.22 | 15 |
| Il2ra | NM_008367.2 | 70.06 | 101.32 | 68.4 | 88.26 | 142.19 | 95.02 | 135.41 | 131.43 | 147.94 | 152.61 |
| Aqp9 | NM_001271843.1 | 65.23 | 30.49 | 48.23 | 19.93 | 19.3 | 52.26 | 43.59 | 42.29 | 27.87 | 39.64 |
| C1qa | NM_007572.2 | 4016.43 | 5002.92 | 5234.55 | 4993.81 | 5844 | 6201.07 | 5669.48 | 7068.79 | 6270.11 | 6620.55 |
| Aplnr | NM_011784.3 | 32.61 | 60.99 | 45.6 | 20.88 | 28.44 | 34.44 | 27.82 | 41.14 | 39.66 | 19.82 |
| Ccr4 | NM_009916.2 | 15 | 15.74 | 16.66 | 15.18 | 15 | 15 | 15 | 15 | 15 | 15 |
| Nfatc2 | NM_001136073.2 | 93.01 | 143.62 | 109.62 | 98.7 | 84.3 | 90.27 | 89.03 | 98.29 | 114.7 | 88.19 |
| H2-Q1 | NM_010390.3 | 25.37 | 18.69 | 15 | 15 | 15 | 15 | 15 | 15 | 15 | 15 |
| Arid1a | NM_001080819.1 | 431.24 | 394.45 | 396.38 | 370.12 | 363.6 | 368.19 | 339.45 | 377.15 | 423.44 | 344.85 |
| Pdgfb | NM_011057.3 | 740.47 | 939.4 | 923.43 | 884.5 | 1342.68 | 1150.9 | 1278.95 | 1045.75 | 1134.17 | 1258.51 |
| Cd38 | NM_007646.4 | 189.65 | 217.39 | 155.22 | 149 | 125.94 | 165.09 | 139.12 | 185.15 | 139.36 | 146.66 |
| Chd9 | NM_177224.2 | 386.54 | 471.18 | 482.33 | 540.95 | 525.09 | 467.96 | 472.07 | 507.44 | 460.96 | 495.48 |
| Pdk1 | NM_172665.5 | 1970.16 | 1806.01 | 1354.02 | 3333 | 3315.05 | 2463.33 | 2141.48 | 2026.35 | 1879.21 | 1901.64 |
| Mfge8 | NM_008594.2 | 485.6 | 601.02 | 641.93 | 529.56 | 504.77 | 590.3 | 520.3 | 499.44 | 451.31 | 589.62 |
| Mki67 | NM_001081117.2 | 6740.35 | 3297.25 | 3322.79 | 4199.47 | 2779.81 | 2975.23 | 3511.31 | 3654.97 | 2443.08 | 2339.64 |
| Brca2 | NM_009765.3 | 289.91 | 197.72 | 223.62 | 241.05 | 216.33 | 168.66 | 179 | 179.43 | 173.66 | 202.15 |
| Rrm2 | NM_009104.2 | 1412.09 | 389.53 | 398.14 | 588.4 | 358.52 | 353.94 | 443.32 | 462.87 | 344.11 | 324.04 |
| Fgfr1 | NM_010206.2 | 137.71 | 136.73 | 167.5 | 244.85 | 203.13 | 161.53 | 148.39 | 171.43 | 154.37 | 179.36 |
| Tlr1 | NM_030682.1 | 177.57 | 200.67 | 192.93 | 117.68 | 126.96 | 190.03 | 128.92 | 180.58 | 197.25 | 141.71 |
| Cxcl2 | NM_009140.2 | 15 | 32.46 | 35.08 | 66.43 | 36.56 | 46.32 | 40.81 | 59.43 | 40.74 | 26.76 |
| Thbs1 | NM_011580.4 | 839.52 | 1094.82 | 1065.5 | 1860.1 | 1515.34 | 1833.84 | 1734.33 | 1542.9 | 1760.22 | 2130.55 |
| Mmp12 | NM_008605.3 | 218.64 | 165.26 | 203.45 | 474.52 | 242.74 | 834.97 | 333.88 | 525.73 | 478.11 | 486.56 |
| P4ha2 | NM_001136076.2 | 362.38 | 380.68 | 171.88 | 933.85 | 537.27 | 532.1 | 485.98 | 449.16 | 355.9 | 599.53 |
| Rptor | NM_028898.2 | 217.43 | 224.28 | 274.49 | 270.47 | 274.22 | 222.1 | 314.4 | 323.44 | 257.28 | 267.56 |
| Srebf1 | NM_011480.4 | 495.26 | 524.29 | 520.91 | 585.55 | 553.52 | 595.05 | 599.13 | 523.44 | 569.23 | 566.82 |
| Epcam | NM_008532.2 | 281.45 | 90.5 | 51.74 | 45.55 | 32.5 | 48.7 | 51.01 | 30.86 | 60.03 | 30.72 |
| Oasl1 | NM_145209.3 | 119.59 | 207.55 | 128.91 | 155.64 | 170.63 | 223.29 | 265.25 | 225.15 | 196.18 | 211.07 |
| Prf1 | NM_011073.2 | 20.54 | 17.71 | 15 | 15.18 | 15.23 | 15 | 15 | 15 | 16.08 | 15 |
| Tap2 | NM_011530.3 | 674.04 | 757.42 | 846.26 | 847.49 | 848.06 | 837.34 | 866.24 | 875.46 | 1034.48 | 1022.66 |
| Tlk2 | NM_011903.3 | 53.15 | 60 | 59.63 | 85.41 | 95.47 | 80.76 | 65.85 | 77.72 | 61.1 | 68.38 |
| Rnls | NM_001146342.2 | 20.54 | 15 | 17.54 | 16.13 | 15.23 | 15 | 15 | 15 | 15 | 15 |
| Itpk1 | NM_172584.3 | 173.94 | 182.96 | 126.28 | 298 | 290.47 | 247.05 | 179 | 219.44 | 187.6 | 164.5 |
| Dll1 | NM_007865.3 | 192.06 | 175.09 | 241.16 | 79.72 | 138.13 | 161.53 | 195.69 | 145.15 | 154.37 | 169.45 |
| Spry4 | NM_011898.2 | 62.81 | 71.81 | 77.17 | 97.75 | 101.56 | 91.45 | 106.66 | 122.29 | 130.78 | 104.05 |
| Mlh1 | NM_026810.2 | 138.91 | 160.34 | 159.61 | 190.76 | 160.47 | 182.91 | 153.03 | 190.86 | 183.31 | 187.29 |
| Stat1 | NM_009283.4 | 222.26 | 192.8 | 183.28 | 264.78 | 229.54 | 237.54 | 186.42 | 235.44 | 292.66 | 213.05 |
| Ccnd2 | NM_009829.3 | 939.78 | 963.99 | 612.99 | 1305.87 | 893.76 | 1073.7 | 894.06 | 1162.32 | 1221 | 1229.77 |
| Ccr5 | NM_009917.5 | 200.52 | 234.11 | 231.52 | 226.82 | 270.16 | 390.76 | 289.36 | 352.01 | 358.05 | 318.1 |
| Tmem140 | NM_197986.2 | 15 | 15 | 24.55 | 30.37 | 33.52 | 21.38 | 18.55 | 21.71 | 24.66 | 24.77 |
| Il18bp | NM_010531.1 | 242.8 | 249.85 | 234.15 | 259.09 | 220.39 | 254.17 | 201.26 | 230.86 | 229.41 | 202.15 |
| Il18 | NM_008360.1 | 233.13 | 165.26 | 189.42 | 175.57 | 243.75 | 236.36 | 234.64 | 244.58 | 200.46 | 215.04 |
| Nectin1 | NM_021424.2 | 224.68 | 341.33 | 361.31 | 410.93 | 480.4 | 458.46 | 485.98 | 529.16 | 414.86 | 433.05 |
| Hmgb1 | NM_010439.3 | 1384.31 | 1155.81 | 1240.89 | 1175.85 | 1215.72 | 1166.34 | 1168.58 | 1269.75 | 1134.17 | 1036.54 |
| Jag1 | NM_013822.5 | 503.71 | 663.97 | 632.29 | 485.9 | 493.6 | 621.18 | 603.77 | 576.02 | 490.97 | 628.26 |
| Aph1b | NM_177583.4 | 55.57 | 83.61 | 65.77 | 134.76 | 116.8 | 90.27 | 102.02 | 89.15 | 83.62 | 87.2 |
| Lag3 | NM_008479.2 | 149.79 | 148.53 | 142.94 | 204.99 | 156.41 | 175.78 | 140.04 | 172.58 | 196.18 | 215.04 |
| Rock1 | NM_009071.2 | 549.62 | 511.51 | 474.43 | 432.76 | 461.1 | 498.84 | 420.13 | 449.16 | 538.14 | 488.54 |
| Hsd11b1 | NM_001044751.1 | 206.56 | 182.96 | 172.76 | 76.87 | 108.67 | 225.67 | 115.93 | 104 | 127.57 | 83.24 |
| Bid | NM_007544.3 | 258.5 | 227.23 | 293.78 | 308.44 | 336.18 | 334.94 | 284.73 | 370.3 | 332.32 | 310.17 |
| C7 | XM_356827.7 | 15 | 23.61 | 15 | 16.13 | 15 | 15 | 15 | 15 | 15 | 15 |
| Marco | NM_010766.2 | 181.19 | 183.95 | 113.13 | 223.02 | 97.5 | 90.27 | 147.46 | 86.86 | 63.25 | 153.6 |
| Cd86 | NM_019388.3 | 282.66 | 301 | 258.7 | 356.84 | 427.59 | 359.88 | 347.79 | 379.44 | 311.95 | 378.54 |
| Ptger4 | NM_001136079.1 | 344.27 | 381.66 | 327.98 | 465.97 | 343.29 | 441.83 | 387.67 | 393.15 | 384.85 | 361.7 |
| Olr1 | NM_001301094.1 | 43.49 | 61.97 | 44.72 | 72.13 | 74.14 | 99.77 | 52.86 | 93.72 | 68.61 | 105.04 |
| Twist2 | NM_007855.2 | 25.37 | 49.18 | 27.19 | 66.43 | 53.83 | 59.39 | 56.57 | 50.29 | 32.16 | 58.47 |
| Gbp3 | NM_018734.3 | 324.94 | 449.54 | 365.69 | 476.41 | 437.74 | 435.89 | 435.9 | 406.87 | 497.41 | 410.25 |
| Bbs1 | NM_001033128.3 | 15 | 18.69 | 15 | 15 | 15 | 15 | 15 | 15 | 15 | 17.84 |
| Tigit | NM_001146325.1 | 41.07 | 35.41 | 37.71 | 63.59 | 71.09 | 42.76 | 27.82 | 40 | 61.1 | 57.48 |
| Parp12 | NM_172893.3 | 1007.43 | 1151.87 | 1133.03 | 1136.94 | 1303.07 | 1203.16 | 1254.84 | 1277.75 | 1312.12 | 1139.59 |
| Ubb | NM_011664.4 | 27.78 | 41.31 | 29.82 | 28.47 | 16.25 | 22.57 | 15.77 | 26.29 | 38.59 | 28.74 |
| Clec14a | NM_025809.5 | 48.32 | 79.68 | 64.02 | 44.6 | 49.77 | 43.95 | 45.44 | 62.86 | 46.1 | 26.76 |
| Trem1 | NM_021406.5 | 25.37 | 18.69 | 15 | 49.35 | 38.59 | 29.69 | 48.23 | 36.57 | 22.51 | 55.49 |
| Wnt4 | NM_009523.2 | 1662.14 | 1169.58 | 1594.31 | 1255.57 | 2057.69 | 1837.4 | 1553.47 | 1726.91 | 1656.24 | 1764.89 |
| Ccnb1 | NM_172301.3 | 18.12 | 17.71 | 20.17 | 26.57 | 27.42 | 19 | 24.11 | 38.86 | 18.22 | 33.69 |
| Id4 | NM_031166.2 | 76.1 | 46.23 | 55.25 | 48.4 | 55.86 | 66.51 | 48.23 | 43.43 | 48.24 | 45.58 |
| Cd27 | NM_001042564.1 | 43.49 | 57.05 | 37.71 | 37.01 | 33.52 | 38.01 | 28.75 | 33.14 | 32.16 | 17.84 |
| Lamc2 | NM_008485.3 | 166.7 | 133.78 | 198.19 | 151.85 | 130 | 138.96 | 164.16 | 118.86 | 152.22 | 180.35 |
| Tnfsf13b | NM_033622.1 | 129.25 | 154.44 | 126.28 | 162.28 | 190.94 | 140.15 | 155.81 | 160 | 174.74 | 193.24 |
| Kit | NM_001122733.1 | 184.82 | 162.3 | 146.45 | 136.66 | 110.7 | 123.52 | 140.04 | 138.29 | 112.56 | 239.81 |
| Vtcn1 | NM_178594.3 | 150.99 | 163.29 | 279.75 | 85.41 | 98.52 | 118.77 | 204.97 | 117.72 | 98.62 | 109 |
| Parp4 | NM_001145978.2 | 617.26 | 659.06 | 668.24 | 687.1 | 813.53 | 700.75 | 700.22 | 717.74 | 680.72 | 731.32 |
| Cdkn2b | NM_007670.4 | 187.23 | 140.66 | 212.22 | 111.04 | 163.52 | 199.54 | 166.01 | 169.15 | 155.44 | 170.44 |
| Slc7a5 | NM_011404.3 | 648.67 | 579.38 | 592.82 | 588.4 | 508.84 | 451.33 | 552.76 | 456.01 | 478.11 | 481.6 |
| Relb | NM_009046.2 | 742.89 | 791.85 | 857.66 | 745.94 | 797.28 | 792.21 | 715.99 | 702.88 | 750.4 | 661.96 |
| Cd1d1 | NM_007639.3 | 74.89 | 104.27 | 59.63 | 70.23 | 47.74 | 86.7 | 51.94 | 65.14 | 66.46 | 52.52 |
| Cxcr4 | NM_009911.3 | 594.31 | 523.31 | 448.12 | 488.75 | 525.09 | 437.08 | 410.86 | 490.3 | 481.33 | 468.72 |
| S100a8 | NM_013650.2 | 351.51 | 57.05 | 20.17 | 304.64 | 32.5 | 43.95 | 23.19 | 105.15 | 41.81 | 47.57 |
| Ero1l | NM_015774.3 | 1273.18 | 1618.13 | 729.63 | 3972.65 | 2695.51 | 2660.49 | 1858.6 | 1508.62 | 1061.28 | 2035.42 |
| Wdr76 | NM_030234.2 | 184.82 | 132.79 | 127.16 | 134.76 | 99.53 | 136.59 | 143.75 | 150.86 | 124.35 | 120.9 |
| Tnfrsf4 | NM_011659.2 | 44.69 | 74.76 | 68.4 | 85.41 | 99.53 | 85.52 | 50.08 | 91.43 | 68.61 | 63.42 |
| Kir3dl1 | NM_177749.3 | 15 | 15.74 | 15 | 15 | 15 | 15 | 15 | 15 | 15 | 15 |
| Fgf13 | NM_010200.2 | 15 | 18.69 | 15 | 15 | 15 | 15 | 15 | 15 | 15 | 15 |
| Tnfsf9 | NM_009404.3 | 15 | 30.49 | 27.19 | 44.6 | 26.41 | 40.38 | 38.03 | 54.86 | 38.59 | 36.67 |
| Itgam | NM_001082960.1 | 258.5 | 357.07 | 309.57 | 471.67 | 370.71 | 508.34 | 490.62 | 437.73 | 279.79 | 433.05 |
| Ifi35 | NM_027320.4 | 695.78 | 1011.21 | 1002.36 | 932.9 | 1044.08 | 979.87 | 1154.67 | 1194.32 | 1019.47 | 1061.31 |
| Ifngr2 | NM_008338.3 | 858.85 | 1072.2 | 1190.03 | 1029.7 | 1130.41 | 1305.3 | 1137.05 | 1275.47 | 1130.96 | 1281.3 |
| Il18r1 | NM_001161842.1 | 669.2 | 619.71 | 1102.33 | 476.41 | 424.54 | 466.77 | 520.3 | 579.45 | 534.93 | 475.66 |
| Api5 | NM_007466.2 | 1358.94 | 1500.09 | 1621.49 | 1550.72 | 1538.7 | 1383.69 | 1518.23 | 1296.04 | 1236.01 | 1301.12 |
| C5ar1 | NM_007577.3 | 175.15 | 256.74 | 165.74 | 276.17 | 285.4 | 340.88 | 303.28 | 347.44 | 260.5 | 305.21 |
| Clec5a | NM_001038604.1 | 147.37 | 187.88 | 192.93 | 189.81 | 209.22 | 251.8 | 202.18 | 264.01 | 221.9 | 221.97 |
| S100a9 | NM_009114.2 | 344.27 | 60 | 26.31 | 352.09 | 39.61 | 48.7 | 24.11 | 108.57 | 49.31 | 59.46 |
| Itgax | NM_021334.2 | 144.95 | 180.01 | 175.39 | 184.11 | 208.21 | 239.92 | 194.76 | 212.58 | 209.04 | 350.8 |
| Ticam1 | NM_174989.4 | 122 | 113.12 | 107.87 | 121.48 | 123.91 | 169.84 | 123.35 | 110.86 | 115.78 | 148.64 |
| Psmb5 | NM_011186.1 | 909.59 | 995.47 | 941.85 | 1076.2 | 1013.61 | 921.67 | 1199.19 | 1146.32 | 1081.65 | 1019.69 |
| Msh2 | NM_008628.2 | 515.79 | 496.75 | 569.14 | 468.82 | 499.7 | 448.96 | 557.4 | 442.3 | 490.97 | 491.51 |
| Gbp2 | NM_010260.1 | 2059.55 | 1712.56 | 2032.78 | 2267.24 | 1856.59 | 2618.92 | 1781.63 | 2372.64 | 2603.88 | 2295.04 |
| Cd68 | NM_009853.1 | 769.46 | 910.87 | 883.09 | 1003.13 | 939.47 | 1185.34 | 988.66 | 1153.18 | 935.85 | 1077.16 |
| Selp | NM_011347.1 | 15 | 50.17 | 35.96 | 36.06 | 15 | 24.94 | 25.04 | 37.72 | 28.94 | 17.84 |
| Pold1 | NM_011131.3 | 184.82 | 181.98 | 204.33 | 213.53 | 163.52 | 161.53 | 172.51 | 178.29 | 151.15 | 124.86 |
| Ulbp1 | NM_029975.2 | 96.64 | 157.39 | 159.61 | 173.67 | 142.19 | 157.97 | 179 | 203.43 | 143.65 | 153.6 |
| Jag2 | NM_010588.2 | 53.15 | 60 | 56.13 | 72.13 | 98.52 | 84.33 | 76.98 | 102.86 | 72.9 | 68.38 |
| Vcam1 | NM_011693.3 | 515.79 | 437.73 | 405.15 | 466.92 | 437.74 | 478.65 | 401.58 | 436.58 | 384.85 | 487.55 |
| Gls | NM_001081081.2 | 250.05 | 238.05 | 284.13 | 319.82 | 196.02 | 245.86 | 245.77 | 221.72 | 181.17 | 287.38 |
| Tpm1 | NM_024427.4 | 5701.52 | 6014.13 | 5796.68 | 9203.71 | 7988.02 | 7026.54 | 8101.25 | 8261.97 | 7586.52 | 7065.49 |
| Rb1 | NM_009029.2 | 543.58 | 444.62 | 386.74 | 409.03 | 457.04 | 389.57 | 521.23 | 398.87 | 404.14 | 412.24 |
| Parp9 | NM_030253.2 | 489.22 | 542.98 | 532.31 | 540.95 | 517.98 | 551.1 | 616.75 | 668.59 | 701.09 | 553.94 |
| NEG_D | ERCC_00076.1 | 13 | 16 | 19 | 13 | 16 | 16 | 9 | 12 | 15 | 11 |
| Angpt2 | NM_007426.3 | 181.19 | 143.62 | 123.65 | 229.67 | 263.05 | 237.54 | 203.11 | 189.72 | 275.5 | 249.72 |
| Ptgs2 | NM_011198.4 | 128.04 | 201.65 | 107.87 | 244.85 | 155.39 | 262.49 | 209.6 | 188.58 | 190.82 | 221.97 |
| Ccl1 | NM_011329.2 | 56.77 | 40.33 | 60.51 | 64.53 | 50.78 | 86.7 | 49.15 | 98.29 | 98.62 | 55.49 |
| Tnfrsf14 | NM_178931.2 | 781.54 | 371.83 | 402.52 | 368.22 | 392.04 | 452.52 | 341.3 | 469.73 | 414.86 | 408.27 |
| Il4 | NM_021283.1 | 15 | 15 | 15 | 16.13 | 15 | 19 | 15 | 15 | 19.3 | 18.83 |
| Gimap4 | NM_001243199.1 | 466.27 | 576.43 | 432.34 | 441.3 | 416.41 | 375.32 | 251.34 | 453.73 | 376.27 | 345.84 |
| Pcx | NM_008797.3 | 160.66 | 235.1 | 146.45 | 125.27 | 168.6 | 203.1 | 157.67 | 157.72 | 151.15 | 146.66 |
| Klrd1 | NM_010654.2 | 83.35 | 81.64 | 79.8 | 100.6 | 84.3 | 91.45 | 91.82 | 109.72 | 85.76 | 70.36 |
| Mmp7 | NM_010810.4 | 15 | 41.31 | 15 | 15 | 15 | 15 | 18.55 | 18.29 | 21.44 | 15 |
| Il33 | NM_133775.1 | 198.1 | 297.07 | 315.7 | 223.02 | 267.11 | 458.46 | 313.48 | 334.87 | 236.91 | 375.57 |
| Ercc3 | NM_133658.1 | 213.81 | 225.26 | 186.79 | 177.47 | 206.18 | 167.47 | 201.26 | 202.29 | 200.46 | 160.53 |
| Kdr | NM_010612.2 | 260.92 | 293.13 | 251.69 | 210.69 | 293.52 | 249.42 | 199.4 | 267.44 | 235.84 | 231.88 |
| Nlrp3 | NM_145827.3 | 68.85 | 80.66 | 74.54 | 75.92 | 78.2 | 123.52 | 84.4 | 91.43 | 93.26 | 76.3 |
| Rsad2 | NM_021384.2 | 1636.77 | 938.42 | 456.02 | 586.5 | 522.04 | 599.8 | 958.98 | 883.46 | 730.03 | 817.54 |
| Cxcl1 | NM_008176.1 | 47.11 | 66.89 | 51.74 | 76.87 | 72.11 | 99.77 | 77.91 | 67.43 | 66.46 | 66.39 |
| Nfkb2 | NM_019408.3 | 392.58 | 473.14 | 512.14 | 448.89 | 463.13 | 490.53 | 498.97 | 520.02 | 549.93 | 520.25 |
| Mrc1 | NM_008625.2 | 674.04 | 709.22 | 704.2 | 603.58 | 539.31 | 837.34 | 648.29 | 748.59 | 558.51 | 511.33 |
| Dusp1 | NM_013642.3 | 547.2 | 626.6 | 569.14 | 1625.69 | 1495.02 | 928.8 | 1085.11 | 981.74 | 656.06 | 934.47 |
| Pfkm | NM_001163487.1 | 277.83 | 293.13 | 278 | 307.49 | 306.72 | 260.11 | 285.65 | 298.29 | 290.51 | 285.39 |
| Ripk2 | NM_138952.3 | 182.4 | 217.39 | 202.58 | 171.77 | 227.5 | 226.85 | 226.3 | 238.86 | 257.28 | 204.14 |
| Blm | NM_001042527.2 | 186.02 | 100.33 | 110.5 | 145.2 | 88.36 | 99.77 | 109.44 | 132.58 | 115.78 | 88.19 |
| Fcgr3 | NM_010188.5 | 259.71 | 304.94 | 299.92 | 399.54 | 402.19 | 402.64 | 399.73 | 491.44 | 425.58 | 441.96 |
| Magea3 | NM_020017.2 | 15 | 15 | 15 | 18.98 | 18.28 | 15 | 15 | 20.57 | 16.08 | 15 |
| Hk1 | NM_001146100.1 | 536.33 | 420.03 | 415.68 | 473.57 | 498.68 | 501.22 | 461.87 | 451.44 | 502.77 | 420.16 |
| Zeb1 | NM_011546.3 | 333.39 | 368.87 | 404.28 | 367.28 | 329.07 | 306.43 | 346.87 | 357.73 | 367.7 | 375.57 |
| Siglec1 | NM_011426.3 | 94.22 | 199.68 | 129.79 | 114.83 | 99.53 | 140.15 | 123.35 | 126.86 | 113.63 | 123.87 |
| Notch2 | NM_010928.1 | 536.33 | 674.79 | 606.85 | 701.33 | 766.81 | 671.06 | 665.91 | 625.16 | 701.09 | 659.97 |
| Prlr | NM_011169.5 | 18.12 | 15 | 15 | 15 | 15 | 17.82 | 15 | 15 | 15 | 15 |
| Tnfsf18 | NM_183391.3 | 18.12 | 40.33 | 40.34 | 15 | 19.3 | 32.07 | 27.82 | 15 | 26.8 | 15 |
| Ldhb | NM_008492.2 | 3823.16 | 3789.08 | 4776.78 | 5133.31 | 4568.36 | 4192.64 | 4425.78 | 4549.85 | 4680.34 | 4840.8 |
| Fbp1 | NM_019395.3 | 31.41 | 17.71 | 17.54 | 15 | 17.27 | 21.38 | 15 | 25.14 | 15 | 18.83 |
| Tdo2 | NM_019911.2 | 19.33 | 26.56 | 15 | 24.67 | 15 | 20.19 | 19.48 | 22.86 | 23.58 | 15.86 |
| H2-Q10 | NM_010391.4 | 28.99 | 42.3 | 29.82 | 42.71 | 23.36 | 33.26 | 28.75 | 33.14 | 28.94 | 24.77 |
| Psmc4 | NM_011874.2 | 841.94 | 930.55 | 903.26 | 933.85 | 818.61 | 931.17 | 985.88 | 780.6 | 852.24 | 889.87 |
| Acvr1c | NM_001033369.3 | 15 | 15 | 15 | 15 | 15 | 15 | 15 | 15 | 15 | 15 |
| Ccl28 | NM_020279.3 | 15 | 15 | 15 | 15 | 15 | 15 | 15 | 15 | 15 | 15 |
| Dsc3 | NM_007882.3 | 15 | 38.36 | 15 | 15 | 15 | 15 | 15 | 15 | 15 | 15 |
| Hc | NM_010406.2 | 15 | 15 | 15 | 15 | 15 | 15 | 15 | 15 | 15 | 15 |
| Ces3a | NM_001164681.1 | 15 | 15 | 15 | 15.18 | 15 | 15 | 15 | 15 | 15 | 15 |
| Serpina1a | NM_009243.4 | 15 | 15 | 15 | 15 | 16.25 | 15 | 15 | 15 | 15 | 15 |
| Ccl9 | NM_011338.2 | 498.88 | 723.98 | 598.08 | 1161.61 | 916.11 | 1148.52 | 943.21 | 1134.89 | 841.52 | 894.83 |
| Csf1r | NM_001037859.1 | 1089.57 | 1084.98 | 1210.2 | 1182.49 | 1262.44 | 1308.86 | 1251.13 | 1339.47 | 1174.91 | 1342.74 |
| Irf2 | NM_008391.4 | 372.05 | 431.83 | 391.12 | 466.92 | 394.07 | 520.22 | 409.93 | 410.3 | 434.16 | 487.55 |
| Cd163 | NM_053094.2 | 67.65 | 107.22 | 37.71 | 45.55 | 43.67 | 73.64 | 79.76 | 43.43 | 52.53 | 34.68 |
| Cd247 | NM_001113391.2 | 111.13 | 93.45 | 86.82 | 66.43 | 86.33 | 62.95 | 38.95 | 88 | 61.1 | 53.51 |
| Clec7a | NM_020008.2 | 285.08 | 293.13 | 338.51 | 335.01 | 354.46 | 395.51 | 316.26 | 349.73 | 412.72 | 333.95 |
| Cd44 | NM_009851.2 | 51.94 | 45.25 | 38.59 | 70.23 | 61.95 | 54.64 | 43.59 | 57.14 | 49.31 | 43.6 |
| Ccl3 | NM_011337.1 | 36.24 | 49.18 | 18.42 | 26.57 | 21.33 | 34.44 | 21.33 | 15 | 36.45 | 21.8 |
| H2-Eb1 | NM_010382.2 | 7294.8 | 6488.26 | 10094.64 | 9053.76 | 8974.21 | 9960.2 | 6546.85 | 9036.85 | 9294.21 | 12223.39 |
| Dtx3l | NM_001013371.2 | 742.89 | 901.04 | 898 | 824.71 | 808.45 | 934.73 | 896.84 | 1068.6 | 1069.85 | 971.13 |
| Sdha | NM_023281.1 | 2725.13 | 2547.69 | 2838.71 | 2585.16 | 2795.05 | 2684.24 | 2786.05 | 2626.37 | 2353.03 | 2552.69 |
| Trem2 | NM_031254.2 | 166.7 | 180.01 | 181.53 | 236.31 | 215.32 | 266.05 | 249.48 | 216.01 | 234.77 | 297.29 |
| Pla1a | NM_134102.4 | 112.34 | 135.75 | 132.42 | 162.28 | 181.8 | 192.41 | 231.86 | 208.01 | 225.12 | 171.43 |
| Cd300a | NM_170758.3 | 164.28 | 170.17 | 145.57 | 148.05 | 118.83 | 205.48 | 145.61 | 187.43 | 144.72 | 140.72 |
| Tnks | NM_175091.3 | 890.26 | 941.37 | 954.13 | 1145.48 | 983.14 | 1033.31 | 960.84 | 1024.03 | 935.85 | 1073.2 |
| Csf3r | NM_001252651.1 | 89.39 | 98.37 | 87.7 | 102.5 | 74.14 | 108.08 | 57.5 | 100.57 | 87.9 | 72.34 |
| Wnt7b | NM_009528.2 | 15 | 15 | 15 | 16.13 | 15.23 | 15 | 23.19 | 15 | 17.15 | 17.84 |
| Psmb10 | NM_013640.3 | 399.83 | 371.83 | 523.54 | 469.77 | 413.37 | 481.03 | 389.53 | 505.16 | 519.92 | 528.18 |
| Uba7 | NM_023738.4 | 545.99 | 451.5 | 471.8 | 458.38 | 533.21 | 490.53 | 496.18 | 563.45 | 572.45 | 495.48 |
| Hnf1a | NM_009327.1 | 21.74 | 18.69 | 19.29 | 16.13 | 15 | 15 | 15 | 15 | 15 | 15 |
| NEG_H | ERCC_00154.1 | 13 | 21 | 21 | 25 | 22 | 24 | 17 | 26 | 14 | 21 |
| Hes1 | NM_008235.2 | 393.79 | 451.5 | 543.71 | 521.02 | 603.29 | 562.98 | 545.34 | 590.88 | 575.66 | 567.82 |
| Runx3 | NM_019732.2 | 184.82 | 191.81 | 221.87 | 279.02 | 255.94 | 191.22 | 233.72 | 214.86 | 168.3 | 290.35 |
| Cdkn1c | NM_009876.3 | 32.61 | 32.46 | 33.32 | 22.78 | 30.47 | 26.13 | 28.75 | 25.14 | 28.94 | 20.81 |
| Cxcl9 | NM_008599.2 | 761.01 | 514.46 | 550.73 | 805.73 | 640.87 | 975.12 | 374.69 | 749.74 | 1011.97 | 821.5 |
| Tymp | NM_138302.1 | 25.37 | 21.64 | 15 | 15 | 15 | 15 | 17.62 | 15 | 17.15 | 15 |
| Spp1 | NM_009263.3 | 3739.81 | 6311.2 | 6269.36 | 4097.92 | 4949.22 | 5615.53 | 5505.33 | 3882.41 | 4458.44 | 6380.74 |
| Oaz1 | NM_008753.4 | 3512.71 | 3061.17 | 3034.27 | 2954.34 | 2696.53 | 3103.51 | 2907.55 | 3006.95 | 2735.74 | 3069.97 |
| Fap | NM_007986.2 | 85.76 | 124.93 | 105.23 | 74.02 | 75.16 | 127.09 | 120.57 | 116.58 | 116.85 | 93.15 |
| Cd274 | NM_021893.2 | 411.91 | 351.17 | 293.78 | 409.98 | 412.35 | 435.89 | 328.32 | 418.3 | 549.93 | 369.63 |
| Il15 | NM_008357.2 | 60.4 | 57.05 | 77.17 | 75.92 | 59.92 | 80.76 | 56.57 | 77.72 | 66.46 | 56.48 |
| H2-Ab1 | NM_207105.3 | 20794.84 | 19114.59 | 24567.05 | 23246.54 | 24321.57 | 23671.21 | 18560.07 | 23740.16 | 23575.36 | 25749.88 |
| Cd28 | NM_007642.4 | 80.93 | 95.42 | 64.02 | 72.13 | 80.24 | 49.88 | 49.15 | 61.72 | 76.11 | 44.59 |
| Prkaa2 | NM_178143.1 | 38.65 | 38.36 | 15 | 15 | 15 | 15 | 15 | 15 | 16.08 | 15 |
| Abcf1 | NM_013854.1 | 567.74 | 675.78 | 668.24 | 661.48 | 665.25 | 624.74 | 733.61 | 725.74 | 685.01 | 657.99 |
| E2f3 | NM_010093.3 | 97.84 | 80.66 | 85.06 | 104.39 | 100.55 | 98.58 | 97.38 | 98.29 | 75.04 | 94.14 |
| Zc3h12a | NM_153159.2 | 321.31 | 370.84 | 397.26 | 324.57 | 436.73 | 426.39 | 421.99 | 429.73 | 354.83 | 385.48 |
| Hif1a | NM_010431.2 | 229.51 | 246.9 | 223.62 | 235.36 | 261.02 | 235.17 | 246.7 | 256.01 | 251.92 | 216.03 |
| Ccl22 | NM_009137.2 | 198.1 | 242.97 | 354.29 | 321.72 | 325.01 | 315.93 | 159.52 | 404.58 | 397.71 | 248.73 |
| Casp1 | NM_009807.2 | 549.62 | 449.54 | 546.34 | 506.78 | 455.01 | 560.6 | 474.85 | 554.3 | 472.75 | 571.78 |
| Csf2rb | NM_007780.4 | 158.24 | 204.6 | 218.36 | 209.74 | 255.94 | 218.54 | 181.78 | 228.58 | 243.34 | 282.42 |
| Col17a1 | NM_007732.2 | 577.4 | 676.76 | 671.75 | 819.96 | 689.62 | 581.98 | 692.8 | 724.59 | 493.12 | 742.22 |
| Tnfrsf1a | NM_011609.2 | 1168.09 | 1349.59 | 1420.67 | 1460.56 | 1387.37 | 1761.39 | 1440.33 | 1491.47 | 1461.13 | 1691.55 |
| Cx3cl1 | NM_009142.3 | 494.05 | 491.83 | 637.55 | 463.13 | 540.32 | 495.28 | 434.97 | 384.01 | 466.32 | 513.31 |
| Comp | NM_016685.2 | 24.16 | 42.3 | 24.55 | 46.5 | 25.39 | 34.44 | 62.14 | 35.43 | 45.02 | 31.71 |
| Ihh | NM_010544.2 | 15 | 15 | 15 | 15.18 | 15 | 19 | 15 | 19.43 | 18.22 | 15 |
| Adgre1 | NM_010130.1 | 703.03 | 727.91 | 756.81 | 689 | 830.79 | 802.9 | 779.98 | 904.03 | 765.41 | 848.25 |
| Cmtm6 | NM_026036.3 | 968.77 | 1097.77 | 1209.32 | 1098.98 | 1287.83 | 1375.38 | 1278.02 | 1390.9 | 1370.01 | 1152.48 |
| Ccnd1 | NM_007631.1 | 2032.98 | 2350.96 | 3605.17 | 2483.62 | 2893.56 | 2921.79 | 2989.16 | 3675.54 | 3194.55 | 2373.33 |
| Myd88 | NM_010851.2 | 384.13 | 542 | 500.74 | 457.43 | 501.73 | 577.23 | 571.31 | 582.88 | 453.45 | 598.53 |
| Pcdhb11 | NM_053136.3 | 19.33 | 33.44 | 23.68 | 17.08 | 32.5 | 28.51 | 21.33 | 21.71 | 15 | 22.79 |
| Ube2c | NM_026785.2 | 2443.68 | 1518.78 | 1347 | 2123.93 | 1158.85 | 1342.12 | 1559.04 | 1805.77 | 1324.99 | 1388.32 |
| Tap1 | NM_001161730.1 | 1323.91 | 1300.41 | 1375.07 | 1787.03 | 1420.88 | 1473.96 | 1201.97 | 1488.05 | 1679.82 | 1377.42 |
| Tcf3 | NM_001164147.1 | 35.03 | 29.51 | 28.94 | 36.06 | 42.66 | 42.76 | 30.61 | 53.72 | 24.66 | 38.65 |
| Pecam1 | NM_008816.2 | 491.64 | 697.42 | 563.01 | 511.53 | 586.03 | 617.61 | 513.81 | 677.74 | 633.55 | 525.2 |
| Ikbkb | NM_010546.2 | 310.44 | 354.12 | 320.97 | 316.03 | 366.65 | 336.12 | 289.36 | 370.3 | 347.33 | 386.47 |
| Fyn | NM_008054.2 | 309.23 | 305.92 | 356.04 | 379.61 | 432.66 | 395.51 | 376.54 | 473.16 | 301.23 | 336.92 |
| Prom1 | NM_001163577.1 | 193.27 | 93.45 | 57.88 | 42.71 | 15 | 47.51 | 46.37 | 30.86 | 57.89 | 15 |
| Tlr3 | NM_126166.2 | 99.05 | 137.71 | 136.81 | 149 | 124.92 | 150.84 | 125.21 | 189.72 | 141.5 | 174.41 |
| Foxp3 | NM_054039.2 | 51.94 | 51.15 | 64.02 | 50.3 | 59.92 | 58.2 | 51.94 | 60.57 | 56.82 | 51.53 |
| Il2 | NM_008366.3 | 21.74 | 22.62 | 15 | 16.13 | 15 | 17.82 | 15 | 21.71 | 15 | 15 |
| Smad5 | NM_008541.2 | 1529.26 | 1501.07 | 1788.99 | 1184.39 | 1551.9 | 1490.59 | 1472.79 | 1530.33 | 1553.32 | 1347.69 |
| Elob | NM_026305.2 | 2513.74 | 2966.74 | 3232.46 | 3192.54 | 2945.36 | 3149.83 | 3357.36 | 3124.67 | 3163.46 | 3401.94 |
| Cd74 | NM_001042605.1 | 42087.34 | 38736.76 | 50901.17 | 44730.71 | 48478.61 | 49858.02 | 37320.47 | 49049.5 | 53742.43 | 54818.46 |
| Anln | NM_028390.3 | 498.88 | 588.23 | 562.13 | 844.64 | 659.15 | 546.35 | 708.57 | 764.59 | 543.5 | 509.35 |
| Adora2a | NM_009630.2 | 41.07 | 36.4 | 38.59 | 29.42 | 29.45 | 34.44 | 29.68 | 45.72 | 47.17 | 35.67 |
| Fut4 | NM_010242.3 | 90.6 | 73.77 | 80.68 | 60.74 | 80.24 | 89.08 | 61.21 | 77.72 | 97.55 | 78.29 |
| POS_D | ERCC_00092.1 | 404 | 374 | 243 | 310 | 284 | 212 | 298 | 191 | 321 | 266 |
| Cxcl13 | NM_018866.2 | 345.47 | 354.12 | 335.87 | 196.45 | 241.72 | 268.42 | 191.05 | 310.87 | 231.55 | 117.92 |
| Axin1 | NM_001159598.1 | 416.74 | 535.11 | 498.99 | 535.25 | 552.51 | 555.85 | 536.99 | 542.87 | 467.39 | 523.22 |
| POS_B | ERCC_00112.1 | 5788 | 5059 | 3672 | 4122 | 3684 | 2825 | 4159 | 2124 | 4436 | 2941 |
| Wnt10a | NM_009518.1 | 178.78 | 153.45 | 247.3 | 174.62 | 261.02 | 228.04 | 207.75 | 193.15 | 214.4 | 247.74 |
| Cd7 | NM_009854.1 | 42.28 | 40.33 | 30.69 | 33.22 | 25.39 | 33.26 | 27.82 | 44.57 | 31.09 | 42.61 |
| Gng4 | NM_010317.2 | 15 | 15 | 15 | 15 | 15 | 15 | 15 | 18.29 | 15 | 16.85 |
| Lck | NM_001162433.1 | 111.13 | 124.93 | 103.48 | 98.7 | 102.58 | 91.45 | 71.41 | 134.86 | 108.27 | 90.18 |
| Il11 | NM_008350.2 | 15 | 21.64 | 15 | 15.18 | 18.28 | 17.82 | 26.9 | 19.43 | 18.22 | 15 |
| Tapbpl | NM_145391.2 | 88.18 | 87.55 | 88.57 | 93.95 | 81.25 | 90.27 | 79.76 | 106.29 | 110.42 | 88.19 |
| Areg | NM_009704.3 | 15 | 15.74 | 15 | 15 | 15 | 15 | 15 | 15 | 19.3 | 15 |
| Oas1a | NM_145211.2 | 678.87 | 1164.66 | 963.77 | 848.43 | 914.08 | 1065.38 | 1140.76 | 1241.18 | 1040.91 | 1181.21 |
| Itga1 | NM_001033228.3 | 143.75 | 123.94 | 119.27 | 108.19 | 128.99 | 116.4 | 123.35 | 146.29 | 153.3 | 134.77 |
| Erbb2 | NM_001003817.1 | 48.32 | 46.23 | 49.99 | 45.55 | 35.55 | 49.88 | 33.39 | 40 | 30.02 | 51.53 |
| Il12rb2 | NM_008354.3 | 18.12 | 18.69 | 18.42 | 18.98 | 20.31 | 29.69 | 19.48 | 36.57 | 18.22 | 16.85 |
| Gpsm3 | NM_134116.5 | 387.75 | 437.73 | 406.03 | 476.41 | 461.1 | 452.52 | 475.78 | 515.44 | 525.28 | 447.91 |
| Lilrb4a | NM_013532.3 | 695.78 | 867.59 | 818.2 | 762.07 | 867.36 | 1305.3 | 900.55 | 1064.03 | 1038.77 | 859.16 |
| POS_C | ERCC_00002.1 | 1709 | 1568 | 1178 | 1352 | 1171 | 879 | 1282 | 668 | 1361 | 1022 |
| Cd4 | NM_013488.2 | 70.06 | 95.42 | 80.68 | 46.5 | 79.22 | 83.14 | 53.79 | 52.57 | 73.97 | 74.32 |
| Icam5 | NM_008319.2 | 15 | 15 | 15 | 21.83 | 15 | 15 | 18.55 | 15 | 16.08 | 15 |
| Lamb3 | NM_008484.2 | 27.78 | 24.59 | 15 | 15 | 15 | 15 | 15 | 15 | 17.15 | 15 |
| POS_A | ERCC_00117.1 | 15303 | 13967 | 10457 | 12321 | 10422 | 8665 | 11675 | 6249 | 11852 | 9915 |
| Ncam1 | NM_001113204.1 | 64.02 | 85.58 | 64.02 | 52.2 | 67.03 | 68.89 | 76.05 | 60.57 | 68.61 | 113.96 |
| Jak1 | NM_146145.2 | 1131.85 | 1285.65 | 1138.29 | 1078.1 | 1224.86 | 1044 | 1222.38 | 1124.61 | 1119.16 | 1102.93 |
| Inhba | NM_008380.2 | 86.97 | 169.19 | 93.83 | 112.93 | 153.36 | 232.79 | 240.21 | 153.15 | 224.05 | 219.99 |
| Map3k8 | NM_007746.2 | 107.51 | 151.48 | 127.16 | 129.07 | 127.97 | 147.28 | 120.57 | 136 | 125.42 | 139.72 |
| Snai1 | NM_011427.2 | 25.37 | 36.4 | 41.22 | 27.52 | 37.58 | 58.2 | 41.74 | 43.43 | 36.45 | 47.57 |
| Pdcd1lg2 | NM_021396.2 | 54.36 | 44.26 | 51.74 | 57.89 | 65 | 65.32 | 38.03 | 69.72 | 68.61 | 45.58 |
| Nfam1 | NM_028728.2 | 132.87 | 125.91 | 124.53 | 111.04 | 103.6 | 141.34 | 130.77 | 142.86 | 101.84 | 124.86 |
| Psmb9 | NM_013585.2 | 1435.04 | 1299.42 | 1304.91 | 1726.29 | 1293.93 | 1411.01 | 1079.55 | 1522.33 | 1560.83 | 1398.23 |
| Cdh1 | NM_009864.2 | 62.81 | 64.92 | 41.22 | 20.88 | 16.25 | 35.63 | 18.55 | 21.71 | 25.73 | 15 |
| Tlr5 | NM_016928.2 | 26.57 | 21.64 | 15 | 29.42 | 31.48 | 17.82 | 36.17 | 29.72 | 17.15 | 21.8 |
| Cd48 | NM_007649.4 | 399.83 | 394.45 | 366.57 | 326.47 | 332.11 | 382.45 | 323.68 | 420.58 | 399.85 | 366.65 |
| Thbd | NM_009378.3 | 420.37 | 411.17 | 535.82 | 294.2 | 402.19 | 494.09 | 421.06 | 422.87 | 378.41 | 543.04 |
| Edn1 | NM_010104.3 | 280.24 | 277.39 | 199.07 | 582.71 | 328.05 | 378.88 | 337.59 | 357.73 | 314.1 | 244.77 |
| Pik3cg | NM_001146200.1 | 275.41 | 339.36 | 353.41 | 372.97 | 394.07 | 393.13 | 351.5 | 435.44 | 306.59 | 374.58 |
| Crabp2 | NM_007759.2 | 15 | 15 | 15 | 15 | 15 | 17.82 | 15 | 15 | 15 | 17.84 |
| Nbn | NM_013752.3 | 512.17 | 495.77 | 561.25 | 480.21 | 490.55 | 532.1 | 542.56 | 461.73 | 423.44 | 410.25 |
| Gbp2b | NM_010259.2 | 84.56 | 54.1 | 55.25 | 78.77 | 57.89 | 96.21 | 29.68 | 70.86 | 118.99 | 74.32 |
| Atf3 | NM_007498.3 | 130.46 | 134.76 | 110.5 | 152.79 | 146.25 | 160.34 | 160.45 | 123.43 | 99.7 | 144.68 |
| H2-DMa | NM_010386.4 | 432.45 | 322.64 | 527.05 | 443.2 | 404.23 | 535.66 | 323.68 | 438.87 | 480.25 | 560.88 |
| Ifngr1 | NM_010511.2 | 639 | 686.6 | 671.75 | 860.77 | 768.84 | 717.38 | 766.07 | 707.45 | 632.48 | 743.21 |
| Hells | NM_008234.3 | 208.98 | 201.65 | 206.08 | 263.83 | 205.16 | 188.85 | 255.98 | 274.29 | 212.26 | 186.3 |
| Tie1 | NM_011587.2 | 74.89 | 89.51 | 94.71 | 89.21 | 65 | 95.02 | 76.98 | 97.15 | 105.06 | 67.38 |
| Il24 | NM_053095.2 | 15 | 15 | 15 | 15 | 15 | 15 | 15 | 21.71 | 15 | 25.76 |
| Bax | NM_007527.3 | 286.28 | 329.53 | 283.26 | 307.49 | 287.43 | 302.87 | 305.13 | 260.58 | 316.24 | 223.96 |
| Prkx | NM_016979.1 | 428.82 | 553.8 | 459.53 | 448.89 | 572.82 | 627.11 | 614.9 | 521.16 | 581.02 | 556.91 |
| Clec4e | NM_019948.2 | 27.78 | 37.38 | 23.68 | 81.62 | 37.58 | 57.01 | 31.53 | 38.86 | 26.8 | 38.65 |
| Birc5 | NM_001012273.1 | 1676.63 | 1031.87 | 988.33 | 1218.56 | 954.7 | 914.54 | 1362.42 | 1274.32 | 975.52 | 887.89 |
| Ifit1 | NM_008331.2 | 276.62 | 514.46 | 328.86 | 335.01 | 358.52 | 369.38 | 495.26 | 454.87 | 616.4 | 524.21 |
| Mfng | NM_008595.2 | 43.49 | 77.71 | 74.54 | 47.45 | 62.97 | 65.32 | 52.86 | 59.43 | 75.04 | 65.4 |
| Nlrc5 | NM_001033207.3 | 520.63 | 524.29 | 480.57 | 591.25 | 526.1 | 600.99 | 393.24 | 669.73 | 773.98 | 587.63 |
| Apoe | NM_001305844.1 | 18543.22 | 21020.94 | 22309.77 | 18107.53 | 22116.62 | 21709.11 | 22902.38 | 23400.72 | 23634.32 | 23871.04 |
| Dll4 | NM_019454.3 | 109.92 | 120.01 | 122.77 | 92.06 | 105.63 | 127.09 | 89.96 | 149.72 | 134 | 106.03 |
| Herc6 | NM_025992.2 | 782.75 | 919.73 | 757.69 | 731.7 | 938.45 | 798.15 | 877.36 | 996.6 | 956.22 | 891.86 |
| Pik3r1 | NM_001077495.1 | 930.12 | 861.69 | 827.85 | 1063.86 | 1109.08 | 1001.25 | 1002.57 | 1017.17 | 1008.75 | 904.74 |
| Hdc | NM_008230.6 | 118.38 | 161.32 | 171.01 | 150.9 | 114.77 | 163.91 | 116.86 | 217.15 | 167.23 | 135.76 |
| Bnip3l | NM_009761.3 | 1775.68 | 1433.2 | 1204.06 | 1782.28 | 1780.42 | 1688.93 | 1661.06 | 1602.33 | 1321.77 | 1654.89 |
| Ripk3 | NM_019955.1 | 71.27 | 99.35 | 85.94 | 117.68 | 111.72 | 104.52 | 101.09 | 122.29 | 88.98 | 105.04 |
| Ccl5 | NM_013653.3 | 618.47 | 746.6 | 782.24 | 745.94 | 760.72 | 749.45 | 606.55 | 845.74 | 772.91 | 765.01 |
| Tnfsf12 | NM_011614.3 | 242.8 | 326.58 | 358.68 | 279.96 | 302.66 | 391.95 | 357.99 | 348.58 | 270.14 | 399.35 |
| Ctsw | NM_009985.5 | 72.48 | 89.51 | 90.33 | 93.95 | 91.41 | 98.58 | 58.43 | 114.29 | 88.98 | 77.29 |
| Ccl8 | NM_021443.2 | 421.57 | 660.04 | 525.3 | 355.89 | 501.73 | 821.9 | 375.62 | 794.31 | 609.97 | 271.52 |
| Sgk1 | NM_001161849.2 | 1249.02 | 1418.45 | 1320.7 | 2052.76 | 1332.52 | 1666.37 | 1826.14 | 1665.19 | 1423.61 | 1765.88 |
| Sf3a1 | NM_026175.5 | 123.21 | 130.83 | 156.1 | 161.34 | 125.94 | 118.77 | 159.52 | 136 | 152.22 | 139.72 |
| Irf8 | NM_008320.3 | 430.03 | 406.25 | 377.09 | 284.71 | 341.26 | 407.39 | 294 | 409.16 | 426.65 | 316.11 |
| Batf3 | NM_030060.2 | 26.57 | 28.53 | 33.32 | 39.86 | 23.36 | 40.38 | 30.61 | 19.43 | 43.95 | 34.68 |
| Tnfsf13 | NM_023517.2 | 351.51 | 422.98 | 619.13 | 413.78 | 530.16 | 515.47 | 412.71 | 507.44 | 467.39 | 610.43 |
| Nod2 | NM_145857.2 | 25.37 | 30.49 | 15.79 | 37.96 | 28.44 | 21.38 | 19.48 | 25.14 | 22.51 | 25.76 |
| H2-DMb1 | NM_010387.2 | 548.41 | 469.21 | 648.07 | 566.57 | 542.35 | 600.99 | 456.3 | 634.31 | 652.85 | 583.67 |
| Bcl2 | NM_009741.3 | 123.21 | 169.19 | 151.71 | 113.88 | 153.36 | 159.15 | 131.7 | 178.29 | 164.02 | 150.62 |
| Robo4 | NM_028783.2 | 237.97 | 195.75 | 235.02 | 246.75 | 286.41 | 236.36 | 249.48 | 258.29 | 271.22 | 279.45 |
| Ccl26 | NM_001013412.2 | 15 | 15 | 18.42 | 15 | 20.31 | 19 | 15 | 22.86 | 16.08 | 15.86 |
| Tnfrsf25 | NM_033042.3 | 143.75 | 172.14 | 163.99 | 73.08 | 92.42 | 171.03 | 126.13 | 120 | 128.64 | 118.91 |
| C2 | NM_013484.2 | 875.76 | 780.05 | 1177.75 | 698.49 | 548.45 | 761.33 | 587.07 | 673.16 | 691.44 | 661.96 |
| Irf4 | NM_013674.1 | 97.84 | 71.81 | 64.02 | 62.64 | 101.56 | 59.39 | 31.53 | 42.29 | 53.6 | 84.23 |
| Fam124b | NM_173425.3 | 15 | 19.67 | 15 | 15.18 | 15 | 15 | 23.19 | 25.14 | 26.8 | 15 |
| H2-D1 | NM_010380.3 | 8703.27 | 10132.74 | 9449.2 | 13135.55 | 9408.91 | 11145.54 | 9260.56 | 10206.03 | 9234.18 | 11252.26 |
| Hdac4 | XM_006529302.1 | 163.07 | 233.13 | 255.19 | 202.14 | 225.47 | 285.05 | 224.44 | 272.01 | 221.9 | 242.78 |
| Cd80 | NM_009855.2 | 78.52 | 106.24 | 107.87 | 138.56 | 117.81 | 110.46 | 80.69 | 128 | 121.14 | 110.99 |
| Cdk6 | NM_009873.2 | 728.39 | 758.41 | 806.8 | 763.02 | 966.89 | 785.08 | 818.01 | 797.74 | 721.45 | 741.23 |
| Tapbp | NM_009318.2 | 1584.83 | 1816.83 | 2160.82 | 1795.57 | 1979.49 | 1956.17 | 2040.38 | 1893.77 | 2072.17 | 1712.36 |
| Cd70 | NM_011617.1 | 15 | 15 | 15 | 15 | 19.3 | 15 | 15 | 15 | 27.87 | 15 |
| Fcgrt | NM_010189.3 | 492.84 | 553.8 | 555.99 | 610.23 | 540.32 | 631.87 | 561.11 | 550.87 | 577.81 | 464.76 |
| Dkk1 | NM_010051.3 | 15 | 15 | 15 | 15 | 15 | 15 | 15 | 18.29 | 16.08 | 15 |
| Wnt11 | NM_001285792.1 | 696.99 | 903.99 | 1248.79 | 917.71 | 1126.35 | 880.1 | 1126.85 | 1214.89 | 1030.19 | 714.48 |
| Gzmb | NM_013542.2 | 15 | 15 | 15 | 15 | 16.25 | 19 | 15 | 15 | 15 | 15 |
| Gata3 | NM_008091.3 | 217.43 | 171.16 | 199.07 | 188.86 | 196.02 | 207.85 | 187.34 | 166.86 | 157.58 | 178.37 |
| Lama1 | NM_008480.2 | 15 | 17.71 | 26.31 | 15 | 24.38 | 32.07 | 23.19 | 30.86 | 46.1 | 27.75 |
| Tnfrsf11a | NM_009399.3 | 132.87 | 142.63 | 150.84 | 123.37 | 123.91 | 172.22 | 127.99 | 154.29 | 161.87 | 118.91 |
| Col11a1 | NM_007729.2 | 27.78 | 26.56 | 15 | 25.62 | 27.42 | 24.94 | 26.9 | 22.86 | 20.37 | 24.77 |
| H2afx | NM_010436.2 | 855.23 | 799.72 | 739.27 | 1054.37 | 883.61 | 805.27 | 937.65 | 986.32 | 839.37 | 744.2 |
| Ccl11 | NM_011330.3 | 70.06 | 136.73 | 87.7 | 44.6 | 24.38 | 97.39 | 76.98 | 74.29 | 88.98 | 47.57 |
| Glud1 | NM_008133.4 | 2054.72 | 2323.42 | 2636.13 | 2077.43 | 2175.5 | 2515.59 | 2636.73 | 2410.36 | 2404.49 | 2194.96 |
| B2m | NM_009735.3 | 36651.58 | 43534.1 | 40040.07 | 55836.29 | 43041.88 | 44485.97 | 40443.18 | 44125.93 | 44748.37 | 43545.39 |
| Tnfrsf8 | NM_009401.2 | 15 | 15 | 15 | 15 | 19.3 | 15 | 15 | 15 | 15 | 15 |
| Cdh5 | NM_009868.4 | 190.86 | 244.93 | 183.28 | 232.51 | 240.71 | 261.3 | 181.78 | 226.29 | 290.51 | 200.17 |
| Tnfrsf17 | NM_011608.1 | 15 | 16.72 | 15 | 18.98 | 15 | 15 | 15 | 15 | 20.37 | 16.85 |
| Rasal1 | NM_013832.4 | 72.48 | 76.73 | 78.05 | 69.28 | 65 | 79.58 | 86.25 | 93.72 | 71.82 | 112.97 |
| Ripk1 | NM_009068.3 | 230.72 | 314.77 | 236.78 | 287.56 | 298.6 | 275.55 | 311.62 | 249.15 | 277.65 | 259.63 |
| Fas | NM_007987.2 | 122 | 125.91 | 127.16 | 98.7 | 92.42 | 121.15 | 114.08 | 133.72 | 142.58 | 173.42 |
| Col11a2 | NM_009926.2 | 26.57 | 16.72 | 15 | 15.18 | 15 | 15 | 15 | 15 | 20.37 | 20.81 |
| Ccl24 | NM_019577.4 | 25.37 | 28.53 | 39.46 | 37.01 | 20.31 | 40.38 | 27.82 | 32 | 22.51 | 27.75 |
| Apc | NM_007462.3 | 73.68 | 93.45 | 60.51 | 72.13 | 57.89 | 74.83 | 76.05 | 68.57 | 66.46 | 61.44 |
| Sfxn1 | NM_027324.5 | 434.86 | 470.19 | 379.72 | 440.35 | 408.29 | 410.95 | 411.79 | 350.87 | 397.71 | 333.95 |
| Deptor | NM_001037937.3 | 660.75 | 692.5 | 677.01 | 520.07 | 533.21 | 652.06 | 554.61 | 537.16 | 614.25 | 481.6 |
| Stc1 | NM_009285.3 | 25.37 | 27.54 | 21.92 | 25.62 | 34.53 | 24.94 | 29.68 | 25.14 | 26.8 | 21.8 |
| A2m | NM_175628.3 | 18.12 | 25.58 | 22.8 | 27.52 | 29.45 | 22.57 | 17.62 | 20.57 | 15 | 25.76 |
| Ccl6 | NM_009139.2 | 77.31 | 71.81 | 82.43 | 134.76 | 100.55 | 138.96 | 108.51 | 117.72 | 103.98 | 82.25 |
| Pdgfa | XM_006504659.3 | 15 | 17.71 | 15 | 15 | 15 | 15 | 22.26 | 15 | 18.22 | 19.82 |
| Ly96 | NM_016923.2 | 417.95 | 461.34 | 418.31 | 482.11 | 503.76 | 541.6 | 518.44 | 522.3 | 490.97 | 531.15 |
| Tbp | NM_013684.3 | 252.46 | 349.2 | 306.06 | 211.63 | 236.64 | 268.42 | 286.58 | 270.87 | 285.15 | 292.33 |
| Ifitm2 | NM_030694.1 | 2993.3 | 3574.64 | 3348.22 | 3198.24 | 3196.22 | 3553.65 | 3349.94 | 3104.1 | 3524.73 | 3185.91 |
| Kat2b | NM_020005.3 | 897.51 | 976.78 | 1046.21 | 923.41 | 1089.78 | 1011.94 | 1046.16 | 988.6 | 909.05 | 955.28 |
| Mill2 | NM_153760.2 | 158.24 | 135.75 | 192.93 | 145.2 | 144.22 | 168.66 | 107.58 | 147.43 | 131.86 | 114.95 |
| Angptl4 | NM_020581.2 | 764.63 | 1174.5 | 866.43 | 1237.54 | 933.37 | 1250.67 | 1214.03 | 1302.9 | 912.27 | 1035.54 |
| Il7r | NM_008372.3 | 131.67 | 186.9 | 114 | 167.03 | 192.97 | 144.9 | 149.32 | 158.86 | 131.86 | 164.5 |
| H2-K1 | NM_001001892.2 | 8857.89 | 10167.17 | 8970.38 | 12753.09 | 9103.2 | 11045.78 | 9070.43 | 10779.76 | 10733.91 | 10304.91 |
| Ren1 | NM_031192.3 | 21.74 | 27.54 | 30.69 | 19.93 | 26.41 | 39.19 | 29.68 | 27.43 | 30.02 | 29.73 |
| Dnmt1 | NM_010066.3 | 265.75 | 223.29 | 233.27 | 235.36 | 261.02 | 207.85 | 247.63 | 264.01 | 227.26 | 172.43 |
| Tslp | NM_021367.1 | 15 | 18.69 | 21.92 | 18.98 | 15.23 | 26.13 | 16.69 | 19.43 | 15 | 17.84 |
| Trat1 | NM_198297.3 | 33.82 | 38.36 | 32.45 | 40.81 | 31.48 | 41.57 | 22.26 | 36.57 | 32.16 | 23.78 |
| Arg1 | NM_007482.3 | 93.01 | 87.55 | 47.36 | 319.82 | 145.24 | 205.48 | 102.02 | 206.86 | 77.18 | 170.44 |
| Ifit2 | NM_008332.2 | 694.57 | 1203.02 | 943.6 | 840.84 | 840.95 | 924.04 | 1315.12 | 1229.75 | 1412.89 | 1080.14 |
| POS_E | ERCC_00035.1 | 68 | 61 | 50 | 66 | 58 | 46 | 54 | 30 | 62 | 47 |
| P2ry13 | NM_028808.3 | 37.45 | 32.46 | 42.09 | 26.57 | 28.44 | 42.76 | 29.68 | 29.72 | 48.24 | 42.61 |
| Tmub2 | NM_028076.2 | 356.34 | 322.64 | 346.4 | 334.06 | 328.05 | 372.94 | 355.21 | 358.87 | 358.05 | 389.44 |
| Ccr2 | NM_009915.2 | 515.79 | 586.26 | 819.08 | 474.52 | 596.18 | 710.26 | 599.13 | 705.16 | 719.31 | 477.64 |
| Cd47 | NM_010581.3 | 2214.17 | 2258.5 | 2173.97 | 2331.77 | 2213.08 | 2446.7 | 2430.84 | 2222.93 | 2018.57 | 2291.08 |
| Tnfsf10 | NM_009425.2 | 131.67 | 176.08 | 109.62 | 122.43 | 123.91 | 154.4 | 128.92 | 162.29 | 150.08 | 156.57 |
| Ncr1 | NM_010746.3 | 33.82 | 30.49 | 30.69 | 26.57 | 25.39 | 55.82 | 25.97 | 34.29 | 36.45 | 22.79 |
| Gzma | NM_010370.2 | 71.27 | 50.17 | 56.13 | 46.5 | 39.61 | 46.32 | 30.61 | 85.72 | 26.8 | 50.54 |
| Ccl12 | NM_011331.2 | 507.34 | 718.08 | 493.73 | 206.89 | 499.7 | 1193.66 | 624.17 | 1003.46 | 1257.45 | 620.34 |
| Pik3r5 | NM_177320.2 | 60.4 | 55.09 | 46.48 | 48.4 | 43.67 | 64.14 | 57.5 | 62.86 | 56.82 | 48.56 |
| Stat4 | NM_011487.5 | 15 | 15 | 15 | 15 | 15 | 15 | 15 | 15 | 16.08 | 15 |
| Sfrp1 | NM_013834.2 | 385.34 | 729.88 | 641.05 | 420.42 | 562.67 | 864.66 | 601.91 | 802.31 | 520.99 | 514.3 |
| Entpd1 | NM_009848.3 | 246.42 | 237.06 | 203.45 | 219.23 | 218.36 | 195.97 | 220.73 | 206.86 | 227.26 | 216.03 |
| Wnt5a | NM_009524.2 | 101.47 | 184.93 | 105.23 | 95.85 | 139.14 | 144.9 | 194.76 | 116.58 | 226.19 | 324.04 |
| Brd3 | NM_001113573.1 | 318.9 | 360.02 | 361.31 | 317.93 | 345.32 | 308.81 | 317.19 | 269.72 | 315.17 | 267.56 |
| Fcgr1 | NM_010186.5 | 379.3 | 585.28 | 474.43 | 415.68 | 450.94 | 630.68 | 554.61 | 610.3 | 612.11 | 574.75 |
| Cxcl5 | NM_009141.2 | 15 | 15 | 15 | 29.42 | 15 | 15 | 15 | 18.29 | 15 | 30.72 |
| Irf1 | NM_008390.2 | 1442.29 | 1353.52 | 1628.51 | 1276.45 | 1486.9 | 1687.75 | 1303.06 | 1619.48 | 1625.15 | 1600.39 |
| Fadd | NM_010175.5 | 77.31 | 82.63 | 99.97 | 109.14 | 92.42 | 87.89 | 101.09 | 83.43 | 81.47 | 88.19 |
| Ptprc | NM_001111316.2 | 1110.1 | 1528.62 | 1295.26 | 1009.77 | 1180.18 | 1323.12 | 1082.33 | 1490.33 | 944.43 | 1282.29 |
| Ezh2 | NM_007971.2 | 805.7 | 633.48 | 563.88 | 748.79 | 633.76 | 555.85 | 623.24 | 636.59 | 631.41 | 468.72 |
| Ddb2 | NM_028119.5 | 128.04 | 129.84 | 151.71 | 185.06 | 148.28 | 130.65 | 110.37 | 152 | 140.43 | 129.81 |
| Fcgr4 | NM_144559.1 | 488.01 | 593.15 | 531.44 | 633.95 | 537.27 | 696 | 654.78 | 741.74 | 630.33 | 778.89 |
| Eif2b4 | NM_010122.2 | 242.8 | 234.11 | 221.87 | 186.96 | 167.58 | 163.91 | 258.76 | 178.29 | 261.57 | 175.4 |
| Itgal | NM_008400.2 | 341.85 | 311.82 | 356.04 | 293.25 | 284.38 | 299.3 | 185.49 | 349.73 | 291.58 | 303.23 |
| Cd209e | NM_130905.2 | 15 | 15 | 15 | 15 | 15 | 20.19 | 15 | 15 | 15 | 15 |
| Fcrlb | NM_001029984.2 | 15 | 15 | 15 | 15 | 15 | 20.19 | 15 | 15 | 15 | 15 |
| Sox10 | XM_128139.6 | 15 | 15 | 15 | 15 | 15 | 15 | 15 | 15 | 20.37 | 15 |
| Tcl1 | NM_009337.3 | 15 | 15 | 15 | 15 | 15 | 15 | 15 | 15 | 15 | 15 |
| Ttc30a1 | NM_030188.3 | 15 | 15 | 15 | 15 | 15 | 15 | 15 | 15 | 16.08 | 15 |
| Sox11 | NM_009234.6 | 15 | 15 | 15 | 15 | 15 | 15 | 15 | 15 | 23.58 | 15 |
| Cd84 | NM_013489.2 | 372.05 | 474.13 | 457.77 | 432.76 | 468.21 | 614.05 | 485.98 | 561.16 | 522.06 | 451.87 |
| Icos | NM_017480.1 | 80.93 | 86.56 | 69.28 | 67.38 | 70.08 | 60.57 | 50.08 | 94.86 | 82.54 | 54.5 |
| Maml2 | NM_001013813.3 | 251.25 | 273.46 | 319.21 | 347.35 | 340.24 | 334.94 | 284.73 | 310.87 | 334.46 | 308.19 |
| Cd6 | NM_001037801.2 | 47.11 | 65.91 | 62.26 | 62.64 | 67.03 | 47.51 | 44.52 | 59.43 | 66.46 | 44.59 |
| H2-Aa | NM_010378.2 | 24703.76 | 20719.94 | 28102.94 | 25726.35 | 26290.9 | 24305.46 | 19082.22 | 23344.72 | 23631.1 | 25751.87 |
| Pdgfrb | NM_001146268.1 | 324.94 | 413.14 | 416.55 | 337.86 | 425.55 | 502.4 | 465.58 | 418.3 | 438.45 | 511.33 |
| Il34 | NM_001135100.2 | 388.96 | 363.96 | 386.74 | 328.37 | 408.29 | 363.44 | 322.75 | 305.15 | 374.13 | 350.8 |
| Hdac3 | NM_010411.2 | 213.81 | 226.24 | 244.67 | 246.75 | 255.94 | 225.67 | 232.79 | 216.01 | 255.14 | 234.86 |
| H2-Q2 | NM_010392.2 | 68.85 | 27.54 | 15 | 20.88 | 15.23 | 32.07 | 15.77 | 20.57 | 25.73 | 15 |
| Hras | NM_001130443.1 | 28.99 | 26.56 | 30.69 | 20.88 | 15.23 | 40.38 | 21.33 | 19.43 | 32.16 | 28.74 |
| Ccl25 | NM_009138.3 | 113.55 | 86.56 | 71.03 | 92.06 | 98.52 | 67.7 | 74.2 | 90.29 | 80.4 | 82.25 |
| Msh6 | NM_010830.1 | 889.05 | 865.63 | 957.64 | 1084.74 | 1030.88 | 913.35 | 1038.74 | 936.03 | 839.37 | 858.16 |
| Pf4 | NM_019932.4 | 103.88 | 91.48 | 114.88 | 130.97 | 48.75 | 136.59 | 109.44 | 140.58 | 86.83 | 118.91 |
| Il1r2 | NM_010555.4 | 42.28 | 49.18 | 34.2 | 73.08 | 60.94 | 51.07 | 37.1 | 41.14 | 45.02 | 73.33 |
| Itga2 | NM_008396.2 | 24.16 | 31.48 | 15.79 | 24.67 | 18.28 | 29.69 | 25.97 | 26.29 | 30.02 | 20.81 |
| Melk | NM_010790.2 | 152.2 | 155.42 | 159.61 | 184.11 | 123.91 | 136.59 | 164.16 | 173.72 | 125.42 | 105.04 |
| Rps6kb1 | NM_001114334.1 | 437.28 | 400.35 | 399.89 | 407.13 | 468.21 | 458.46 | 417.35 | 437.73 | 371.98 | 458.81 |
| Cdkn1a | NM_007669.4 | 388.96 | 571.51 | 386.74 | 670.02 | 523.06 | 661.56 | 637.16 | 488.01 | 558.51 | 660.96 |
| Ly9 | NM_008534.2 | 183.61 | 207.55 | 206.08 | 208.79 | 182.82 | 203.1 | 173.43 | 190.86 | 181.17 | 199.18 |
| Cpa3 | NM_007753.2 | 171.53 | 448.55 | 498.11 | 179.37 | 269.15 | 362.25 | 205.89 | 216.01 | 149.01 | 145.67 |
| Irf9 | NM_008394.2 | 857.64 | 926.61 | 945.36 | 828.5 | 931.34 | 965.61 | 915.39 | 997.74 | 995.89 | 958.25 |
| Twist1 | NM_011658.2 | 59.19 | 87.55 | 64.02 | 55.04 | 72.11 | 110.46 | 78.83 | 92.57 | 60.03 | 74.32 |
| Tpsab1 | NM_031187.4 | 18.12 | 39.35 | 28.94 | 18.98 | 20.31 | 22.57 | 22.26 | 15 | 19.3 | 30.72 |
| Icam2 | NM_010494.1 | 123.21 | 128.86 | 90.33 | 129.07 | 116.8 | 104.52 | 143.75 | 147.43 | 116.85 | 107.02 |
| Siglecf | NM_145581.1 | 15 | 27.54 | 15 | 26.57 | 15 | 28.51 | 20.4 | 35.43 | 24.66 | 23.78 |
| Ifi203 | NM_001045481.1 | 843.15 | 1053.51 | 952.37 | 771.56 | 866.34 | 857.53 | 882 | 928.03 | 965.87 | 818.53 |
| Slamf7 | NM_144539.5 | 241.59 | 240.01 | 261.33 | 218.28 | 252.89 | 292.18 | 217.02 | 253.72 | 303.38 | 216.03 |
| Rad50 | NM_009012.2 | 352.72 | 447.57 | 434.97 | 468.82 | 388.99 | 447.77 | 434.05 | 474.3 | 367.7 | 362.69 |
| Ms4a6b | NM_027209.3 | 378.09 | 573.48 | 471.8 | 595.04 | 576.88 | 460.83 | 518.44 | 572.59 | 526.35 | 503.4 |
| Ptcd2 | NM_026873.2 | 337.02 | 343.3 | 327.98 | 334.06 | 337.19 | 374.13 | 323.68 | 323.44 | 319.46 | 314.13 |
| Rad51c | NM_053269.3 | 66.44 | 67.87 | 36.83 | 61.69 | 48.75 | 41.57 | 50.08 | 52.57 | 39.66 | 63.42 |
| Ms4a2 | NM_001276330.1 | 24.16 | 20.66 | 22.8 | 15 | 24.38 | 34.44 | 21.33 | 19.43 | 21.44 | 17.84 |
| Il23r | NM_144548.1 | 18.12 | 24.59 | 18.42 | 17.08 | 21.33 | 20.19 | 15 | 21.71 | 19.3 | 18.83 |
| Tbc1d10b | NM_144522.5 | 235.55 | 219.36 | 240.29 | 225.87 | 236.64 | 251.8 | 240.21 | 224.01 | 214.4 | 248.73 |
| Samsn1 | NM_023380.2 | 138.91 | 157.39 | 123.65 | 141.41 | 170.63 | 137.78 | 136.33 | 179.43 | 153.3 | 121.89 |
| Ccl17 | NM_011332.2 | 502.51 | 449.54 | 652.46 | 774.41 | 315.86 | 340.88 | 247.63 | 288.01 | 513.49 | 322.06 |
| Mmp13 | NM_008607.1 | 101.47 | 95.42 | 96.47 | 83.51 | 70.08 | 174.59 | 99.24 | 134.86 | 160.8 | 173.42 |
| Rad51 | NM_011234.4 | 358.76 | 337.4 | 406.91 | 459.33 | 350.4 | 350.38 | 440.54 | 499.44 | 386.99 | 320.08 |
| Ccna1 | NM_007628.3 | 15 | 18.69 | 15 | 15 | 15 | 15 | 15 | 18.29 | 15 | 15 |
| Brd4 | NM_001286630.1 | 1715.29 | 1656.49 | 1622.37 | 1833.53 | 1711.36 | 1620.05 | 1657.35 | 1563.48 | 1893.15 | 1556.79 |
| Cd96 | NM_032465.2 | 24.16 | 37.38 | 26.31 | 26.57 | 19.3 | 26.13 | 21.33 | 38.86 | 31.09 | 18.83 |
| Vhl | NM_009507.3 | 146.16 | 147.55 | 185.04 | 146.15 | 148.28 | 178.16 | 127.06 | 170.29 | 152.22 | 171.43 |
| Akt1 | NM_009652.3 | 611.22 | 745.62 | 770.84 | 711.77 | 718.06 | 768.45 | 785.55 | 838.88 | 780.41 | 765.01 |
| Spib | NM_019866.1 | 158.24 | 146.57 | 195.56 | 168.93 | 161.49 | 148.46 | 139.12 | 116.58 | 144.72 | 128.82 |
| Gas1 | NM_008086.1 | 1727.37 | 1326.97 | 1424.18 | 1923.69 | 1824.09 | 1807.71 | 1384.68 | 1499.47 | 1316.41 | 1438.86 |
| Tyms | NM_021288.3 | 147.37 | 136.73 | 155.22 | 146.15 | 151.33 | 118.77 | 179 | 187.43 | 103.98 | 146.66 |
| Ror2 | NM_013846.3 | 47.11 | 44.26 | 23.68 | 37.96 | 33.52 | 61.76 | 31.53 | 15 | 27.87 | 23.78 |
| Tgfbr2 | NM_009371.3 | 869.72 | 1007.27 | 1111.1 | 931.95 | 1139.55 | 1158.02 | 1074.91 | 1033.17 | 1048.41 | 935.46 |
| Map3k5 | NM_008580.4 | 554.45 | 679.71 | 740.15 | 633.95 | 705.87 | 650.87 | 638.08 | 766.88 | 616.4 | 575.74 |
| Tbx21 | NM_019507.2 | 42.28 | 33.44 | 29.82 | 24.67 | 26.41 | 39.19 | 26.9 | 36.57 | 39.66 | 20.81 |
| Ikbkg | NM_001161423.1 | 438.49 | 531.18 | 541.08 | 453.64 | 564.7 | 602.17 | 586.15 | 531.44 | 549.93 | 534.12 |
| Ifih1 | NM_027835.3 | 504.92 | 673.81 | 570.9 | 521.97 | 570.79 | 614.05 | 725.26 | 705.16 | 642.13 | 604.48 |
| Itgae | NM_008399.1 | 171.53 | 203.62 | 290.27 | 243.9 | 277.27 | 186.47 | 134.48 | 249.15 | 273.36 | 159.54 |
| Ifi27 | NM_026790.2 | 2618.83 | 3085.76 | 2423.03 | 2481.72 | 2757.47 | 2949.1 | 2415.07 | 2806.94 | 3001.59 | 2833.13 |
| Casp8 | NM_009812.2 | 815.37 | 949.24 | 971.67 | 872.16 | 917.12 | 929.98 | 994.22 | 986.32 | 981.95 | 1022.66 |
| Ccl4 | NM_013652.2 | 35.03 | 67.87 | 54.37 | 51.25 | 52.81 | 73.64 | 40.81 | 51.43 | 54.67 | 42.61 |
| Sbno2 | NM_183426.1 | 186.02 | 251.82 | 192.93 | 193.6 | 217.35 | 256.55 | 220.73 | 189.72 | 271.22 | 202.15 |
| Dnajc14 | NM_028873.3 | 550.82 | 576.43 | 506.88 | 543.8 | 562.67 | 553.48 | 605.62 | 561.16 | 608.89 | 518.27 |
| Fzd8 | NM_008058.2 | 24.16 | 49.18 | 32.45 | 34.17 | 28.44 | 33.26 | 38.95 | 41.14 | 24.66 | 34.68 |
| Zap70 | NM_009539.3 | 64.02 | 95.42 | 71.91 | 63.59 | 69.06 | 60.57 | 51.01 | 86.86 | 83.62 | 54.5 |
| Myct1 | NM_026793.2 | 36.24 | 66.89 | 51.74 | 54.09 | 63.99 | 70.08 | 48.23 | 58.29 | 64.32 | 65.4 |
| Wnt2b | NM_009520.3 | 43.49 | 32.46 | 46.48 | 40.81 | 33.52 | 57.01 | 36.17 | 40 | 32.16 | 37.66 |
| Ctnnb1 | NM_007614.3 | 5488.92 | 5539.02 | 6484.21 | 5722.66 | 6280.73 | 5883.95 | 5865.18 | 6161.33 | 5818.8 | 5317.45 |
| Kif2c | NM_134471.4 | 85.76 | 89.51 | 75.42 | 113.88 | 82.27 | 72.45 | 99.24 | 86.86 | 86.83 | 72.34 |
| Tmem173 | NM_028261.1 | 236.76 | 377.73 | 381.48 | 277.12 | 337.19 | 435.89 | 333.88 | 406.87 | 403.07 | 417.19 |
| Cxcl11 | NM_019494.1 | 19.33 | 20.66 | 17.54 | 21.83 | 15 | 29.69 | 20.4 | 34.29 | 32.16 | 33.69 |
| Fcer1a | NM_010184.1 | 42.28 | 81.64 | 79.8 | 41.76 | 40.63 | 72.45 | 39.88 | 61.72 | 36.45 | 39.64 |
| Axl | NM_009465.3 | 1886.82 | 2027.34 | 2256.41 | 2181.82 | 2134.88 | 2270.92 | 2295.43 | 2192.07 | 2336.95 | 2594.31 |
| Gmip | NM_198101.1 | 227.09 | 253.79 | 221.87 | 193.6 | 182.82 | 226.85 | 218.88 | 254.86 | 212.26 | 188.28 |
| Eif2ak2 | NM_011163.4 | 601.56 | 710.21 | 730.5 | 620.67 | 688.61 | 717.38 | 669.62 | 737.17 | 719.31 | 703.58 |
| Cdh11 | NM_009866.5 | 223.47 | 258.7 | 342.01 | 184.11 | 296.57 | 301.68 | 268.03 | 290.29 | 283.01 | 233.86 |
| Adm | NM_009627.1 | 95.43 | 71.81 | 53.49 | 157.54 | 98.52 | 115.21 | 85.33 | 61.72 | 67.54 | 108.01 |
| Mrpl19 | NM_026490.2 | 49.53 | 52.13 | 45.6 | 39.86 | 51.8 | 45.13 | 38.03 | 46.86 | 51.46 | 51.53 |
| Cdkn2a | NM_001040654.1 | 31.41 | 31.48 | 50.86 | 31.32 | 34.53 | 38.01 | 28.75 | 37.72 | 38.59 | 40.63 |
| Cxcr6 | NM_030712.4 | 93.01 | 91.48 | 92.96 | 91.11 | 91.41 | 91.45 | 56.57 | 101.72 | 86.83 | 72.34 |
| Prr5 | NM_146061.4 | 118.38 | 120.01 | 115.76 | 108.19 | 144.22 | 115.21 | 92.74 | 117.72 | 101.84 | 157.56 |
| Gpr160 | NM_001134386.1 | 28.99 | 38.36 | 42.97 | 26.57 | 24.38 | 42.76 | 44.52 | 46.86 | 38.59 | 36.67 |
| NEG_C | ERCC_00019.1 | 11 | 16 | 18 | 16 | 10 | 10 | 12 | 8 | 17 | 14 |
| Vcan | NM_001081249.1 | 118.38 | 336.41 | 204.33 | 210.69 | 162.5 | 301.68 | 253.19 | 209.15 | 159.73 | 198.19 |
| Cd200r1 | NM_021325.3 | 74.89 | 74.76 | 63.14 | 68.33 | 68.05 | 71.26 | 59.36 | 60.57 | 101.84 | 67.38 |
| Bcl6b | NM_007528.2 | 60.4 | 53.12 | 39.46 | 44.6 | 56.88 | 79.58 | 48.23 | 72 | 70.75 | 63.42 |
| Cdc25c | NM_009860.2 | 43.49 | 42.3 | 38.59 | 69.28 | 35.55 | 21.38 | 37.1 | 43.43 | 36.45 | 37.66 |
| Fpr1 | NM_013521.2 | 31.41 | 26.56 | 19.29 | 18.03 | 17.27 | 39.19 | 21.33 | 17.14 | 31.09 | 16.85 |
| Fanca | NM_016925.3 | 84.56 | 100.33 | 101.73 | 127.17 | 59.92 | 85.52 | 80.69 | 136 | 67.54 | 94.14 |
| Atm | NM_007499.1 | 102.68 | 115.09 | 136.81 | 109.14 | 123.91 | 127.09 | 119.64 | 134.86 | 141.5 | 115.94 |
| Bnip3 | NM_009760.4 | 579.82 | 625.61 | 342.01 | 819.01 | 954.7 | 1022.62 | 790.18 | 680.02 | 492.05 | 989.96 |
| Nos2 | NM_010927.3 | 42.28 | 40.33 | 15 | 37.01 | 42.66 | 58.2 | 25.04 | 30.86 | 24.66 | 28.74 |
| NEG_E | ERCC_00098.1 | 11 | 9 | 13 | 11 | 5 | 7 | 13 | 8 | 9 | 6 |
| Klrk1 | NM_001083322.1 | 79.72 | 81.64 | 100.85 | 87.31 | 75.16 | 91.45 | 65.85 | 99.43 | 87.9 | 93.15 |
| Tnfrsf10b | NM_020275.3 | 53.15 | 55.09 | 56.13 | 69.28 | 58.91 | 58.2 | 73.27 | 44.57 | 62.18 | 65.4 |
| Slc11a1 | NM_013612.2 | 438.49 | 486.91 | 402.52 | 543.8 | 491.57 | 617.61 | 420.13 | 528.02 | 391.28 | 450.88 |
| Igf2r | NM_010515.2 | 568.94 | 781.03 | 676.13 | 510.58 | 608.37 | 680.56 | 726.19 | 608.02 | 589.6 | 676.82 |
| Hey1 | NM_010423.2 | 205.35 | 247.88 | 256.95 | 233.46 | 173.67 | 178.16 | 223.51 | 257.15 | 189.74 | 231.88 |
| Fgf18 | NM_008005.2 | 450.56 | 366.91 | 486.71 | 254.34 | 348.37 | 431.14 | 319.97 | 318.87 | 504.91 | 265.58 |
| Cmklr1 | NM_008153.3 | 143.75 | 223.29 | 173.64 | 171.77 | 193.99 | 231.6 | 214.24 | 169.15 | 201.54 | 196.21 |
| Tgfb1 | NM_011577.1 | 1275.59 | 1546.32 | 1440.84 | 1628.54 | 1588.46 | 1726.94 | 1650.86 | 1582.91 | 1577.98 | 1699.48 |
| Ifit3 | NM_010501.1 | 925.29 | 1582.72 | 1137.41 | 1064.81 | 1057.28 | 1128.33 | 1573.88 | 1373.76 | 1704.48 | 1352.65 |
| Jup | NM_010593.2 | 15 | 15 | 15 | 20.88 | 19.3 | 15 | 19.48 | 15 | 18.22 | 15 |
| Ccl27a | NM_001048179.1 | 113.55 | 84.6 | 104.36 | 81.62 | 105.63 | 115.21 | 114.08 | 115.43 | 101.84 | 94.14 |
| Kir3dl2 | NM_177748.2 | 15 | 19.67 | 15 | 15 | 15 | 21.38 | 15 | 17.14 | 15 | 20.81 |
| Egf | NM_010113.4 | 161.87 | 152.47 | 73.66 | 77.82 | 46.72 | 96.21 | 56.57 | 50.29 | 93.26 | 36.67 |
| Nid2 | NM_008695.2 | 93.01 | 128.86 | 106.99 | 129.07 | 147.27 | 128.27 | 122.42 | 145.15 | 134 | 100.09 |
| Tgfbr1 | NM_009370.2 | 1513.56 | 1831.59 | 1777.59 | 1633.28 | 1777.37 | 1855.22 | 1855.82 | 1769.2 | 1683.04 | 1898.66 |
| Sirpa | NM_007547.2 | 960.32 | 968.91 | 1090.06 | 835.15 | 989.24 | 1270.86 | 977.53 | 1187.47 | 1154.54 | 1005.82 |
| Il6 | NM_031168.1 | 15 | 25.58 | 17.54 | 15.18 | 15 | 23.75 | 24.11 | 15 | 15 | 15 |
| Notch1 | NM_008714.3 | 153.41 | 212.47 | 183.28 | 138.56 | 156.41 | 219.73 | 161.38 | 196.58 | 199.39 | 146.66 |
| Ccl7 | NM_013654.3 | 60.4 | 134.76 | 71.91 | 42.71 | 56.88 | 181.72 | 63.07 | 98.29 | 137.22 | 63.42 |
| Jak3 | NM_010589.6 | 332.19 | 416.09 | 387.61 | 391 | 407.27 | 413.33 | 380.25 | 385.15 | 452.38 | 448.9 |
| NEG_F | ERCC_00126.1 | 9 | 12 | 7 | 13 | 6 | 4 | 10 | 12 | 15 | 8 |
| Rorc | NM_011281.2 | 625.72 | 688.57 | 753.31 | 577.96 | 786.11 | 703.13 | 593.57 | 658.31 | 679.65 | 718.44 |
| Thy1 | NM_009382.3 | 388.96 | 697.42 | 677.01 | 456.48 | 618.53 | 682.94 | 647.36 | 633.16 | 687.15 | 596.55 |
| Lair1 | NM_001113474.1 | 347.89 | 338.38 | 387.61 | 297.05 | 332.11 | 418.08 | 342.23 | 446.87 | 332.32 | 320.08 |
| Tbxas1 | NM_011539.3 | 217.43 | 199.68 | 254.32 | 216.38 | 237.66 | 290.99 | 201.26 | 266.29 | 251.92 | 211.07 |
| Nkg7 | NM_024253.4 | 79.72 | 79.68 | 65.77 | 79.72 | 70.08 | 57.01 | 47.3 | 66.29 | 92.19 | 53.51 |
| Ikzf2 | NM_011770.4 | 200.52 | 198.7 | 181.53 | 231.56 | 197.03 | 201.91 | 191.05 | 184.01 | 196.18 | 173.42 |
| Pten | NM_008960.2 | 366.01 | 364.94 | 377.97 | 394.8 | 424.54 | 336.12 | 324.61 | 366.87 | 347.33 | 392.42 |
| Cd3g | NM_009850.2 | 169.11 | 212.47 | 174.51 | 140.46 | 175.71 | 154.4 | 115 | 196.58 | 198.32 | 137.74 |
| Itga6 | NM_008397.4 | 2775.87 | 3061.17 | 3712.16 | 3153.63 | 3078.41 | 3310.17 | 3244.21 | 3312.1 | 2982.3 | 3349.42 |
| Pum1 | NM_001159605.1 | 1245.4 | 1197.12 | 1197.05 | 1195.78 | 1334.55 | 1185.34 | 1223.3 | 1197.75 | 1255.31 | 1071.22 |
| Il6ra | NM_010559.2 | 339.43 | 456.42 | 465.66 | 404.29 | 408.29 | 472.71 | 442.39 | 414.87 | 436.3 | 378.54 |
| Cenpf | NM_001081363.2 | 428.82 | 346.25 | 273.61 | 490.65 | 320.94 | 301.68 | 355.21 | 390.87 | 291.58 | 341.88 |
| Epm2aip1 | NM_175266.4 | 460.23 | 441.67 | 492.85 | 451.74 | 507.82 | 435.89 | 436.83 | 465.16 | 474.89 | 425.12 |
| Vegfc | NM_009506.2 | 118.38 | 150.5 | 162.24 | 137.61 | 164.53 | 174.59 | 149.32 | 176.01 | 124.35 | 158.55 |
| Cd8b1 | NM_009858.2 | 18.12 | 37.38 | 17.54 | 19.93 | 22.34 | 21.38 | 15.77 | 27.43 | 24.66 | 17.84 |
| Nfkbie | NM_008690.3 | 366.01 | 383.63 | 377.09 | 331.21 | 413.37 | 403.82 | 378.4 | 370.3 | 444.88 | 424.13 |
| Trim21 | NM_001082552.1 | 345.47 | 402.32 | 345.52 | 297.05 | 310.79 | 359.88 | 363.56 | 373.73 | 443.81 | 319.09 |
| Ube2t | NM_001278115.1 | 217.43 | 177.06 | 151.71 | 223.02 | 149.3 | 161.53 | 222.59 | 211.44 | 157.58 | 170.44 |
| Mx1 | NM_010846.1 | 186.02 | 321.66 | 195.56 | 204.99 | 222.43 | 214.98 | 255.98 | 235.44 | 266.93 | 226.93 |
| NEG_G | ERCC_00144.1 | 32 | 26 | 28 | 29 | 30 | 36 | 26 | 26 | 33 | 22 |
| Socs1 | NM_009896.2 | 227.09 | 254.77 | 224.5 | 170.83 | 172.66 | 261.3 | 183.63 | 234.29 | 319.46 | 252.69 |
| Vsir | NM_001159572.1 | 438.49 | 514.46 | 492.85 | 522.92 | 552.51 | 576.04 | 547.19 | 576.02 | 473.82 | 510.34 |
| Hck | NM_010407.3 | 271.79 | 315.76 | 328.86 | 257.19 | 301.65 | 343.25 | 307.91 | 358.87 | 367.7 | 339.9 |
| Tlr7 | NM_133211.4 | 178.78 | 242.97 | 182.41 | 177.47 | 172.66 | 212.6 | 223.51 | 237.72 | 204.75 | 222.96 |
| Ghr | NM_001048147.1 | 194.48 | 312.81 | 143.82 | 223.97 | 305.71 | 381.26 | 319.04 | 336.01 | 244.42 | 292.33 |
| Myc | NM_001177354.1 | 1583.62 | 1886.67 | 1835.47 | 1290.68 | 1465.57 | 2009.62 | 1848.4 | 1722.34 | 1772.01 | 1583.54 |
| Pnoc | NM_001205075.1 | 20.54 | 15 | 15 | 15 | 16.25 | 15 | 15 | 15 | 15 | 15 |
| Bambi | NM_026505.2 | 15 | 16.72 | 15 | 20.88 | 15 | 15 | 15 | 15 | 15 | 15 |
| Rpl7a | NM_013721.3 | 16791.69 | 17097.09 | 15604.56 | 15927.6 | 18415.62 | 17049.69 | 17359.96 | 16202.78 | 16848.58 | 15035.71 |
| Ccnd3 | NM_007632.2 | 660.75 | 629.55 | 728.75 | 604.53 | 668.29 | 673.44 | 623.24 | 637.73 | 629.26 | 658.98 |
| Cd276 | NM_133983.4 | 115.96 | 137.71 | 125.4 | 198.35 | 199.07 | 155.59 | 178.07 | 156.58 | 152.22 | 183.33 |
| Il10ra | NM_008348.2 | 264.54 | 270.51 | 316.58 | 287.56 | 323.99 | 333.75 | 288.44 | 339.44 | 284.08 | 346.83 |
| Il1b | NM_008361.3 | 118.38 | 143.62 | 260.46 | 138.56 | 264.07 | 173.41 | 212.39 | 262.87 | 174.74 | 316.11 |
| Brip1 | NM_178309.2 | 140.12 | 157.39 | 155.22 | 166.08 | 157.42 | 136.59 | 140.04 | 177.15 | 124.35 | 129.81 |
| Dusp2 | NM_010090.2 | 89.39 | 76.73 | 62.26 | 84.46 | 86.33 | 72.45 | 41.74 | 72 | 54.67 | 51.53 |
| Tpi1 | NM_009415.2 | 89.39 | 56.07 | 73.66 | 88.26 | 79.22 | 85.52 | 63.99 | 57.14 | 69.68 | 62.43 |
| Oas2 | NM_145227.3 | 509.75 | 1031.87 | 694.55 | 440.35 | 657.12 | 821.9 | 779.98 | 886.88 | 821.15 | 717.45 |
| Cd40 | NM_011611.2 | 91.8 | 104.27 | 81.56 | 79.72 | 72.11 | 96.21 | 76.98 | 98.29 | 120.06 | 80.27 |
| Tlr4 | NM_021297.2 | 239.17 | 259.69 | 240.29 | 202.14 | 258.99 | 295.74 | 359.85 | 230.86 | 273.36 | 242.78 |
| NEG_B | ERCC_00041.1 | 10 | 16 | 4 | 9 | 11 | 11 | 13 | 7 | 10 | 10 |
| Slc16a1 | NM_009196.4 | 18.12 | 15 | 15 | 18.98 | 15 | 15 | 15 | 19.43 | 15 | 15 |
| Stat2 | NM_019963.1 | 620.89 | 634.46 | 493.73 | 538.1 | 509.85 | 574.86 | 537.92 | 557.73 | 734.32 | 573.76 |
| Il22ra1 | NM_178257.1 | 172.74 | 185.91 | 122.77 | 220.18 | 166.57 | 228.04 | 267.1 | 213.72 | 179.02 | 209.09 |
| Serpinh1 | NM_009825.2 | 7915.69 | 12378.45 | 11136.47 | 13020.72 | 9559.22 | 11438.91 | 10340.11 | 10503.18 | 9053.02 | 11051.09 |
| Ifna1 | NM_010502.2 | 15 | 22.62 | 20.17 | 15 | 15.23 | 24.94 | 15.77 | 22.86 | 18.22 | 21.8 |
| Cxcl14 | NM_019568.2 | 260.92 | 685.62 | 356.92 | 374.87 | 448.91 | 726.88 | 644.58 | 600.02 | 562.8 | 513.31 |
| Egr1 | NM_007913.5 | 707.86 | 722.01 | 847.14 | 1260.31 | 790.17 | 1080.82 | 911.68 | 851.45 | 944.43 | 1090.05 |
| Il1a | NM_010554.4 | 37.45 | 43.28 | 52.62 | 30.37 | 46.72 | 54.64 | 38.95 | 36.57 | 51.46 | 65.4 |
| Angpt1 | NM_009640.3 | 83.35 | 81.64 | 80.68 | 69.28 | 77.19 | 142.53 | 81.62 | 78.86 | 90.05 | 53.51 |
| H2-T23 | NM_010398.3 | 1430.21 | 1510.91 | 1680.25 | 1515.6 | 1337.6 | 1690.12 | 1217.74 | 1537.19 | 1555.47 | 1629.12 |
| Cx3cr1 | NM_009987.3 | 703.03 | 806.61 | 1085.67 | 662.42 | 840.95 | 1093.89 | 803.17 | 1168.04 | 1020.54 | 726.37 |
| Tlr9 | NM_031178.2 | 159.45 | 201.65 | 212.22 | 137.61 | 157.42 | 176.97 | 179 | 205.72 | 202.61 | 219.99 |
| Pik3ca | NM_008839.2 | 555.66 | 559.71 | 553.36 | 633.95 | 660.17 | 565.35 | 620.46 | 626.3 | 619.61 | 566.82 |
| Hdac11 | NM_144919.2 | 228.3 | 166.24 | 128.04 | 165.13 | 190.94 | 190.03 | 151.17 | 142.86 | 151.15 | 172.43 |
| Smap1 | NM_028534.3 | 1140.3 | 967.93 | 980.44 | 1047.73 | 967.91 | 1150.9 | 1075.84 | 1115.46 | 978.73 | 1058.34 |
| NEG_A | ERCC_00096.1 | 8 | 4 | 6 | 12 | 3 | 6 | 9 | 7 | 11 | 7 |
| Cdc20 | NM_023223.2 | 277.83 | 266.57 | 263.96 | 318.87 | 228.52 | 239.92 | 277.31 | 261.72 | 259.42 | 258.64 |
| Xcl1 | NM_008510.1 | 101.47 | 85.58 | 82.43 | 78.77 | 61.95 | 96.21 | 62.14 | 92.57 | 173.66 | 61.44 |
| Pkp3 | NM_019762.2 | 27.78 | 35.41 | 33.32 | 41.76 | 33.52 | 40.38 | 27.82 | 35.43 | 40.74 | 32.7 |
| Ms4a4a | XM_003086124.1 | 245.21 | 352.15 | 324.47 | 481.16 | 383.91 | 408.57 | 388.6 | 449.16 | 306.59 | 459.8 |
| Tnfrsf18 | NM_009400.2 | 20.54 | 24.59 | 32.45 | 27.52 | 27.42 | 22.57 | 17.62 | 32 | 34.3 | 15 |
| Tnfsf8 | NM_009403.3 | 120.79 | 204.6 | 183.28 | 89.21 | 115.78 | 209.04 | 149.32 | 187.43 | 184.38 | 177.38 |
| Bmp2 | NM_007553.2 | 37.45 | 47.22 | 35.08 | 36.06 | 51.8 | 57.01 | 46.37 | 68.57 | 63.25 | 46.57 |
| Il2rb | NM_008368.3 | 253.67 | 296.08 | 272.73 | 316.98 | 306.72 | 255.36 | 190.13 | 281.15 | 328.03 | 211.07 |
| Neil1 | NM_028347.2 | 45.9 | 48.2 | 35.08 | 55.99 | 47.74 | 38.01 | 56.57 | 45.72 | 48.24 | 35.67 |
| Mdm2 | NM_010786.3 | 273 | 285.26 | 242.92 | 254.34 | 278.29 | 280.3 | 236.5 | 243.44 | 277.65 | 302.24 |
| Cd3d | NM_013487.3 | 90.6 | 122.96 | 96.47 | 93.95 | 100.55 | 103.33 | 70.49 | 110.86 | 80.4 | 70.36 |
| Sh2d1a | NR_132588.1 | 22.95 | 22.62 | 15 | 16.13 | 16.25 | 15 | 15 | 25.14 | 24.66 | 15 |
| Oas3 | NM_145226.2 | 97.84 | 118.04 | 102.6 | 84.46 | 86.33 | 90.27 | 86.25 | 99.43 | 105.06 | 99.1 |
| Cxcl12 | NM_013655.4 | 492.84 | 455.44 | 246.42 | 1519.4 | 862.28 | 864.66 | 618.61 | 654.88 | 775.05 | 926.54 |
| Map3k7 | NM_172688.2 | 1030.38 | 991.54 | 1047.96 | 956.62 | 1067.44 | 984.62 | 1217.74 | 1083.46 | 884.4 | 927.53 |
| Adam12 | NM_007400.2 | 50.73 | 84.6 | 64.02 | 66.43 | 70.08 | 83.14 | 102.95 | 88 | 67.54 | 81.26 |
| Traf1 | NM_009421.4 | 200.52 | 290.18 | 295.53 | 247.7 | 273.21 | 236.36 | 240.21 | 259.44 | 234.77 | 230.89 |
| Rbl2 | NM_011250.4 | 705.44 | 706.27 | 729.63 | 672.86 | 756.65 | 693.63 | 659.41 | 683.45 | 693.58 | 637.18 |
| Lgals9 | NM_010708.1 | 1284.05 | 1557.14 | 1450.49 | 1391.28 | 1470.65 | 1561.85 | 1587.79 | 1307.47 | 1568.33 | 1574.62 |
| Col5a1 | NM_015734.2 | 259.71 | 401.34 | 349.91 | 361.58 | 344.3 | 440.64 | 469.29 | 371.44 | 397.71 | 384.49 |
| Cblc | NM_023224.5 | 15 | 23.61 | 15 | 16.13 | 15 | 22.57 | 15 | 15 | 17.15 | 15 |
| Cmtm4 | NM_153582.5 | 606.39 | 662.01 | 599.84 | 672.86 | 629.7 | 621.18 | 615.82 | 566.87 | 624.97 | 570.79 |
| Mlana | NM_029993.1 | 15 | 17.71 | 15 | 15 | 15 | 15 | 15 | 15 | 15 | 15 |
| Sox2 | NM_011443.3 | 20.54 | 18.69 | 19.29 | 22.78 | 20.31 | 15 | 15 | 18.29 | 31.09 | 18.83 |
| Bbc3 | NM_133234.2 | 22.95 | 24.59 | 22.8 | 31.32 | 21.33 | 22.57 | 20.4 | 17.14 | 25.73 | 19.82 |
| Fcgr2b | NM_001077189.1 | 1404.84 | 1532.55 | 1925.8 | 1894.27 | 1995.74 | 2057.13 | 1638.8 | 1857.2 | 1541.53 | 1973.98 |
| Ccl2 | NM_011333.3 | 159.45 | 376.74 | 212.22 | 207.84 | 239.69 | 494.09 | 292.15 | 266.29 | 345.18 | 250.71 |
| Cybb | NM_007807.5 | 901.13 | 1045.64 | 1080.41 | 947.13 | 1024.78 | 1058.26 | 971.96 | 1118.89 | 1113.8 | 1034.55 |
| Srp54a | NM_011899.4 | 275.41 | 212.47 | 192.93 | 209.74 | 239.69 | 212.6 | 208.68 | 200.01 | 180.1 | 224.95 |
| Gzme | NM_010373.3 | 30.2 | 26.56 | 27.19 | 23.73 | 26.41 | 29.69 | 18.55 | 26.29 | 24.66 | 31.71 |
| Ly6c1 | NM_010741.3 | 805.7 | 1211.88 | 921.68 | 878.8 | 775.95 | 1155.65 | 741.96 | 1026.32 | 1166.33 | 1075.18 |
| Fasl | NM_001205243.1 | 27.78 | 16.72 | 19.29 | 32.27 | 30.47 | 15 | 15 | 38.86 | 24.66 | 19.82 |
| Mmp9 | NM_013599.2 | 15 | 29.51 | 22.8 | 31.32 | 15 | 32.07 | 19.48 | 61.72 | 20.37 | 22.79 |
| Nectin2 | NM_008990.3 | 1117.35 | 1239.42 | 1318.94 | 1288.79 | 1209.63 | 1255.42 | 1302.14 | 1195.47 | 1301.4 | 1249.59 |
| Lilra5 | NM_001081239.2 | 239.17 | 259.69 | 303.43 | 180.32 | 240.71 | 260.11 | 275.45 | 283.44 | 219.76 | 318.1 |
| Glul | NM_008131.3 | 649.88 | 814.48 | 483.2 | 819.01 | 613.45 | 690.06 | 643.65 | 733.74 | 485.61 | 513.31 |
| Fstl3 | NM_031380.2 | 56.77 | 75.74 | 74.54 | 92.06 | 76.17 | 68.89 | 102.02 | 59.43 | 67.54 | 83.24 |
| Pck2 | NM_028994.2 | 1000.18 | 1009.24 | 930.45 | 1117.01 | 958.77 | 952.55 | 1118.5 | 1155.46 | 983.02 | 1027.62 |
| Casp3 | NM_009810.3 | 1043.67 | 1052.52 | 1051.47 | 1321.05 | 1185.25 | 1101.01 | 1092.53 | 1238.9 | 1025.9 | 1055.36 |
| Pias4 | NM_021501.4 | 126.83 | 122.96 | 113.13 | 123.37 | 111.72 | 147.28 | 115 | 109.72 | 102.91 | 125.85 |
| Il21r | NM_021887.1 | 134.08 | 179.03 | 156.98 | 138.56 | 136.1 | 180.53 | 126.13 | 169.15 | 179.02 | 132.79 |
| Il2rg | NM_013563.3 | 734.43 | 1097.77 | 907.65 | 963.27 | 838.92 | 1052.32 | 1015.55 | 1070.89 | 960.51 | 1211.93 |
| Isg15 | NM_015783.3 | 1729.78 | 2018.48 | 2102.06 | 1478.59 | 1445.26 | 1730.5 | 2063.57 | 1819.48 | 1810.6 | 2146.4 |
| Eif4ebp1 | NM_007918.3 | 1169.29 | 1482.38 | 1074.27 | 1654.16 | 1261.43 | 1560.66 | 1454.24 | 1172.61 | 1156.68 | 1335.8 |
| Magea4 | NM_020280.2 | 15 | 15 | 15 | 18.03 | 15 | 15 | 15.77 | 15 | 15 | 15 |
| Got2 | NM_010325.2 | 1566.71 | 1721.42 | 1836.35 | 2090.72 | 1669.71 | 1839.77 | 1808.52 | 1901.77 | 1589.77 | 1764.89 |
| POS_F | ERCC_00034.1 | 43 | 36 | 22 | 38 | 41 | 24 | 37 | 28 | 26 | 27 |
| Cxcl10 | NM_021274.1 | 428.82 | 587.25 | 313.95 | 321.72 | 327.04 | 520.22 | 423.84 | 442.3 | 677.5 | 493.49 |
| Prkacb | NM_011100.3 | 419.16 | 525.28 | 501.62 | 531.46 | 566.73 | 523.78 | 528.64 | 449.16 | 497.41 | 497.46 |
| Cd69 | NM_001033122.3 | 18.12 | 45.25 | 25.43 | 27.52 | 32.5 | 43.95 | 27.82 | 28.57 | 25.73 | 22.79 |
| Tnf | NM_013693.2 | 38.65 | 51.15 | 48.23 | 43.66 | 30.47 | 71.26 | 38.95 | 37.72 | 47.17 | 46.57 |
| Shc2 | NM_001024539.1 | 59.19 | 92.46 | 65.77 | 57.89 | 53.83 | 84.33 | 120.57 | 72 | 92.19 | 110.99 |
| Birc3 | NM_007464.3 | 269.37 | 351.17 | 391.12 | 364.43 | 416.41 | 439.46 | 314.4 | 421.73 | 301.23 | 408.27 |
| Cd8a | NM_001081110.2 | 48.32 | 70.82 | 30.69 | 58.84 | 34.53 | 34.44 | 19.48 | 62.86 | 69.68 | 49.55 |
| Cd3e | NM_007648.4 | 85.76 | 114.11 | 123.65 | 112.93 | 107.66 | 91.45 | 65.85 | 124.58 | 126.5 | 85.22 |
| Irf3 | NM_016849.4 | 674.04 | 659.06 | 655.09 | 641.55 | 615.48 | 772.02 | 660.34 | 673.16 | 652.85 | 644.12 |
| Cxcl3 | NM_203320.2 | 15 | 15 | 15 | 25.62 | 19.3 | 19 | 16.69 | 51.43 | 15 | 15.86 |
| Nt5e | NM_011851.4 | 38.65 | 68.86 | 33.32 | 62.64 | 63.99 | 47.51 | 53.79 | 43.43 | 38.59 | 47.57 |
| Pvrig | XM_011240963.2 | 66.44 | 82.63 | 85.06 | 73.08 | 56.88 | 95.02 | 76.05 | 91.43 | 62.18 | 76.3 |
| Irf7 | NM_016850.2 | 821.41 | 1335.82 | 1139.17 | 831.35 | 824.7 | 920.48 | 1249.27 | 1034.32 | 1193.13 | 1165.36 |
| Cxcr3 | NM_009910.2 | 113.55 | 103.28 | 161.36 | 108.19 | 124.92 | 121.15 | 77.91 | 142.86 | 125.42 | 117.92 |
| Brca1 | NM_009764.3 | 177.57 | 160.34 | 158.73 | 204.99 | 151.33 | 137.78 | 179 | 194.29 | 127.57 | 150.62 |
| Arg2 | NM_009705.3 | 49.53 | 21.64 | 21.92 | 23.73 | 15.23 | 23.75 | 20.4 | 30.86 | 28.94 | 26.76 |
| Eomes | NM_010136.2 | 15 | 29.51 | 15.79 | 23.73 | 23.36 | 19 | 25.04 | 15 | 15 | 15 |
| Taf3 | NM_027748.3 | 102.68 | 110.17 | 92.08 | 99.65 | 102.58 | 109.27 | 102.95 | 105.15 | 117.92 | 89.19 |
| Pirb | NM_011095.2 | 149.79 | 183.95 | 172.76 | 150.9 | 201.1 | 226.85 | 169.72 | 241.15 | 176.88 | 183.33 |
| Mb21d1 | NM_173386.4 | 47.11 | 64.92 | 64.89 | 76.87 | 60.94 | 70.08 | 68.63 | 74.29 | 70.75 | 61.44 |
| Pms2 | NM_008886.2 | 131.67 | 132.79 | 106.99 | 110.09 | 115.78 | 118.77 | 134.48 | 125.72 | 126.5 | 115.94 |
| Mageb4 | NM_001033492.2 | 26.57 | 17.71 | 18.42 | 20.88 | 16.25 | 22.57 | 27.82 | 29.72 | 15 | 29.73 |
| Exo1 | NM_012012.4 | 66.44 | 64.92 | 56.13 | 85.41 | 55.86 | 47.51 | 66.78 | 76.57 | 40.74 | 48.56 |
| Kras | NM_021284.5 | 310.44 | 333.46 | 309.57 | 353.99 | 346.33 | 314.75 | 320.9 | 323.44 | 307.66 | 353.77 |
| Apol6 | NM_001163621.1 | 15 | 15 | 15 | 15 | 15 | 15 | 15 | 15 | 15 | 15 |
| Ccl20 | NM_016960.1 | 15 | 15 | 15 | 15 | 15 | 15 | 15 | 15 | 15 | 15 |
| Ccno | NM_001081062.1 | 15 | 15 | 15 | 15 | 15 | 15 | 15 | 15 | 15 | 15 |
| Ceacam3 | NM_054059.1 | 15 | 15 | 15 | 15 | 15 | 15 | 15 | 15 | 15 | 15 |
| Cntfr | NM_001146080.1 | 15 | 15 | 15 | 15 | 15 | 15 | 15 | 15 | 15 | 15 |
| Csf2 | NM_009969.4 | 15 | 15 | 15 | 15 | 15 | 15 | 15 | 15 | 15 | 15 |
| Csf3 | NM_009971.1 | 15 | 15 | 15 | 15 | 15 | 15 | 15 | 15 | 15 | 15 |
| Cxcl15 | NM_011339.2 | 15 | 15 | 15 | 15 | 15 | 15 | 15 | 15 | 15 | 15 |
| Fcnb | NM_010190.1 | 15 | 15 | 15 | 15 | 15 | 15 | 15 | 15 | 15 | 15 |
| Fpr3 | NM_008042.2 | 15 | 15 | 15 | 15 | 15 | 15 | 15 | 15 | 15 | 15 |
| H2-Pa | ENSMUST00000182803.1 | 15 | 15 | 15 | 15 | 15 | 15 | 15 | 15 | 15 | 15 |
| Ido1 | NM_008324.2 | 15 | 15 | 15 | 15 | 15 | 15 | 15 | 15 | 15 | 15 |
| Ifng | NM_008337.1 | 15 | 15 | 15 | 15 | 15 | 15 | 15 | 15 | 15 | 15 |
| Il17a | NM_010552.3 | 15 | 15 | 15 | 15 | 15 | 15 | 15 | 15 | 15 | 15 |
| Klra1 | NM_016659.3 | 15 | 15 | 15 | 15 | 15 | 15 | 15 | 15 | 15 | 15 |
| Klrb1 | NM_001099918.1 | 15 | 15 | 15 | 15 | 15 | 15 | 15 | 15 | 15 | 15 |
| Magea1 | NM_020015.2 | 15 | 15 | 15 | 15 | 15 | 15 | 15 | 15 | 15 | 15 |
| Mapk10 | NM_009158.2 | 15 | 15 | 15 | 15 | 15 | 15 | 15 | 15 | 15 | 15 |
| Mmp1a | NM_032006.3 | 15 | 15 | 15 | 15 | 15 | 15 | 15 | 15 | 15 | 15 |
| Otoa | NM_139310.1 | 15 | 15 | 15 | 15 | 15 | 15 | 15 | 15 | 15 | 15 |
| Pla2g2a | NM_001082531.1 | 15 | 15 | 15 | 15 | 15 | 15 | 15 | 15 | 15 | 15 |
| Rasgrf1 | NM_011245.2 | 15 | 15 | 15 | 15 | 15 | 15 | 15 | 15 | 15 | 15 |
| Sele | NM_011345.2 | 15 | 15 | 15 | 15 | 15 | 15 | 15 | 15 | 15 | 15 |
| Serpinb5 | NM_009257.3 | 15 | 15 | 15 | 15 | 15 | 15 | 15 | 15 | 15 | 15 |
| Wnt2 | NM_023653.4 | 15 | 15 | 15 | 15 | 15 | 15 | 15 | 15 | 15 | 15 |
| Wnt3a | NM_009522.2 | 15 | 15 | 15 | 15 | 15 | 15 | 15 | 15 | 15 | 15 |
