# Supplementary material for: Tumor-Intrinsic Activity of Chromobox 2 Remodels the Tumor Microenvironment in High-grade Serous Carcinoma
Source: Cancer Res Commun. 2024 Aug 5;4(8):1919–32. doi: 10.1158/2767-9764.CRC-24-0027 (PMC11298703; doi:10.1158/2767-9764.CRC-24-0027)
Supplement: Table S4 — TIMERv2 [file crc-24-0027_table_s4_suppst4.docx]

| Iwanaga, Yamamoto, et al; Table S4 | |  |  |  |
| --- | --- | --- | --- | --- |
| **Cell Type Infiltrates** | **Imputation Method** | **Pearson rho** | **p-value** | **adj.p-value** |
| B cell | TIMER | -0.309 | 6.35E-07 | 1.87E-05 |
| T cell CD4+ effector memory | XCELL | -0.294 | 2.37E-06 | 3.61E-05 |
| Macrophage M1 | XCELL | -0.283 | 5.58E-06 | 5.58E-05 |
| B cell | MCPCOUNTER | 0.213 | 6.97E-04 | 5.86E-03 |
| B cell | XCELL | -0.205 | 1.15E-03 | 9.17E-03 |
| T cell CD8+ central memory | XCELL | -0.204 | 1.19E-03 | 9.54E-03 |
| T cell CD4+ | TIMER | 0.189 | 2.78E-03 | 1.99E-02 |
| T cell CD8+ | TIMER | -0.186 | 3.29E-03 | 2.23E-02 |
| NK cell | QUANTISEQ | 0.167 | 8.45E-03 | 4.08E-02 |
| NK cell | EPIC | -0.166 | 8.59E-03 | 4.11E-02 |
| T cell CD8+ naive | XCELL | 0.157 | 1.28E-02 | 6.34E-02 |
| T cell CD4+ Th2 | XCELL | 0.148 | 1.98E-02 | 9.31E-02 |
| T cell follicular helper | CIBERSORT | 0.136 | 3.13E-02 | 9.74E-02 |
| MDSC | TIDE | 0.433 | 8.92E-13 | 2.38E-11 |
| Myeloid dendritic cell | QUANTISEQ | 0.344 | 2.47E-08 | 9.85E-07 |
| Macrophage | XCELL | -0.327 | 1.28E-07 | 2.08E-06 |
| Plasmacytoid dendritic cell | XCELL | -0.309 | 6.53E-07 | 1.51E-05 |
| Monocyte | QUANTISEQ | -0.298 | 1.72E-06 | 2.55E-05 |
| Macrophage M2 | XCELL | -0.292 | 2.69E-06 | 2.99E-05 |
| Macrophage M2 | TIDE | 0.235 | 1.81E-04 | 1.09E-03 |
| Myeloid dendritic cell | TIMER | -0.239 | 1.42E-04 | 1.57E-03 |
| Myeloid dendritic cell activated | XCELL | -0.224 | 3.66E-04 | 3.32E-03 |
| Macrophage M0 | CIBERSORT | 0.201 | 1.45E-03 | 6.86E-03 |
| Macrophage M0 | CIBERSORT-ABS | 0.191 | 2.53E-03 | 1.14E-02 |
| Mast cell | XCELL | -0.193 | 2.22E-03 | 1.20E-02 |
| Neutrophil | QUANTISEQ | 0.195 | 1.96E-03 | 1.32E-02 |
| Macrophage | EPIC | -0.182 | 4.05E-03 | 1.72E-02 |
| Monocyte | XCELL | -0.166 | 8.58E-03 | 4.36E-02 |
| Macrophage M2 | CIBERSORT | -0.137 | 3.08E-02 | 8.98E-02 |
| Neutrophil | CIBERSORT | -0.146 | 2.09E-02 | 9.04E-02 |
| Endothelial cell | MCPCOUNTER | 0.263 | 2.65E-05 | 3.06E-04 |
| Endothelial cell | EPIC | 0.210 | 8.37E-04 | 6.53E-03 |
| Cancer associated fibroblast | TIDE | 0.163 | 9.82E-03 | 3.71E-02 |
| Class-switched memory B cell | XCELL | -0.137 | 3.11E-02 | 1.08E-01 |
| Neutrophil | MCPCOUNTER | 0.139 | 2.86E-02 | 1.12E-01 |
| Macrophage | TIMER | 0.126 | 4.64E-02 | 1.22E-01 |
| Macrophage M1 | QUANTISEQ | -0.124 | 5.02E-02 | 1.30E-01 |
| Neutrophil | CIBERSORT-ABS | -0.134 | 3.45E-02 | 1.30E-01 |
| Myeloid dendritic cell | XCELL | -0.132 | 3.68E-02 | 1.31E-01 |
| NK cell activated | CIBERSORT | -0.130 | 4.08E-02 | 1.39E-01 |
| Macrophage M1 | CIBERSORT | -0.117 | 6.42E-02 | 1.56E-01 |
| T cell CD4+ naive | CIBERSORT | 0.128 | 4.36E-02 | 1.59E-01 |
| T cell CD4+ naive | CIBERSORT-ABS | 0.128 | 4.36E-02 | 1.59E-01 |
| Common lymphoid progenitor | XCELL | -0.117 | 6.42E-02 | 1.61E-01 |
| T cell follicular helper | CIBERSORT-ABS | 0.114 | 7.33E-02 | 1.85E-01 |
| Cancer associated fibroblast | MCPCOUNTER | 0.105 | 9.77E-02 | 2.17E-01 |
| Cancer associated fibroblast | XCELL | 0.103 | 1.03E-01 | 2.26E-01 |
| Cancer associated fibroblast | EPIC | 0.098 | 1.21E-01 | 2.54E-01 |
| B cell memory | XCELL | -0.100 | 1.16E-01 | 2.78E-01 |
| B cell | EPIC | 0.097 | 1.25E-01 | 2.90E-01 |
| B cell plasma | XCELL | -0.097 | 1.28E-01 | 2.91E-01 |
| T cell CD4+ naive | XCELL | -0.100 | 1.14E-01 | 2.97E-01 |
| T cell CD8+ | QUANTISEQ | -0.103 | 1.04E-01 | 3.00E-01 |
| Endothelial cell | XCELL | 0.097 | 1.26E-01 | 3.28E-01 |
| NK cell activated | CIBERSORT-ABS | -0.095 | 1.35E-01 | 3.38E-01 |
| Mast cell resting | CIBERSORT | -0.079 | 2.13E-01 | 3.66E-01 |
| T cell CD8+ | XCELL | -0.090 | 1.56E-01 | 3.82E-01 |
| Myeloid dendritic cell activated | CIBERSORT | -0.086 | 1.76E-01 | 3.91E-01 |
| T cell gamma delta | CIBERSORT | -0.076 | 2.35E-01 | 4.11E-01 |
| T cell gamma delta | CIBERSORT-ABS | -0.076 | 2.35E-01 | 4.11E-01 |
| Mast cell resting | CIBERSORT-ABS | -0.070 | 2.72E-01 | 4.35E-01 |
| Myeloid dendritic cell activated | CIBERSORT-ABS | -0.078 | 2.22E-01 | 4.47E-01 |
| Macrophage M1 | CIBERSORT-ABS | -0.065 | 3.08E-01 | 4.67E-01 |
| Macrophage/Monocyte | MCPCOUNTER | -0.064 | 3.15E-01 | 4.73E-01 |
| T cell CD4+ central memory | XCELL | -0.073 | 2.54E-01 | 4.76E-01 |
| T cell CD4+ memory | XCELL | -0.065 | 3.06E-01 | 5.34E-01 |
| T cell CD4+ | EPIC | 0.065 | 3.10E-01 | 5.38E-01 |
| B cell naive | CIBERSORT-ABS | 0.065 | 3.06E-01 | 5.43E-01 |
| B cell naive | CIBERSORT | 0.063 | 3.21E-01 | 5.64E-01 |
| Macrophage/Monocyte | MCPCOUNTER | -0.064 | 3.15E-01 | 5.68E-01 |
| Monocyte | MCPCOUNTER | -0.064 | 3.15E-01 | 5.68E-01 |
| T cell CD4+ Th1 | XCELL | 0.060 | 3.46E-01 | 5.80E-01 |
| Mast cell activated | CIBERSORT-ABS | 0.049 | 4.45E-01 | 5.85E-01 |
| T cell regulatory (Tregs) | QUANTISEQ | 0.058 | 3.61E-01 | 5.97E-01 |
| Common myeloid progenitor | XCELL | -0.053 | 4.09E-01 | 6.07E-01 |
| Mast cell activated | CIBERSORT | 0.046 | 4.71E-01 | 6.14E-01 |
| Myeloid dendritic cell | MCPCOUNTER | -0.052 | 4.11E-01 | 6.21E-01 |
| Macrophage M2 | CIBERSORT-ABS | -0.037 | 5.65E-01 | 7.06E-01 |
| NK cell | MCPCOUNTER | -0.040 | 5.31E-01 | 7.13E-01 |
| T cell regulatory (Tregs) | XCELL | 0.043 | 4.96E-01 | 7.17E-01 |
| Granulocyte-monocyte progenitor | XCELL | 0.033 | 6.05E-01 | 7.65E-01 |
| Hematopoietic stem cell | XCELL | 0.032 | 6.19E-01 | 7.73E-01 |
| T cell CD8+ | CIBERSORT | -0.034 | 5.88E-01 | 7.80E-01 |
| T cell CD4+ memory activated | CIBERSORT | -0.032 | 6.15E-01 | 7.95E-01 |
| T cell CD4+ memory activated | CIBERSORT-ABS | -0.032 | 6.17E-01 | 7.95E-01 |
| B cell memory | CIBERSORT-ABS | 0.031 | 6.25E-01 | 8.07E-01 |
| T cell gamma delta | XCELL | -0.025 | 6.93E-01 | 8.21E-01 |
| B cell memory | CIBERSORT | 0.029 | 6.52E-01 | 8.27E-01 |
| T cell CD8+ | MCPCOUNTER | 0.025 | 6.97E-01 | 8.42E-01 |
| Neutrophil | XCELL | -0.023 | 7.15E-01 | 8.62E-01 |
| Myeloid dendritic cell resting | CIBERSORT-ABS | -0.018 | 7.81E-01 | 8.86E-01 |
| Macrophage M2 | QUANTISEQ | -0.013 | 8.35E-01 | 8.91E-01 |
| Myeloid dendritic cell resting | CIBERSORT | -0.016 | 7.97E-01 | 8.94E-01 |
| NK cell | XCELL | -0.016 | 7.97E-01 | 8.94E-01 |
| B cell plasma | CIBERSORT-ABS | 0.017 | 7.85E-01 | 8.97E-01 |
| B cell | QUANTISEQ | -0.017 | 7.84E-01 | 8.97E-01 |
| Neutrophil | TIMER | 0.016 | 7.96E-01 | 9.02E-01 |
| T cell regulatory (Tregs) | CIBERSORT | -0.015 | 8.18E-01 | 9.18E-01 |
| T cell CD8+ effector memory | XCELL | -0.013 | 8.33E-01 | 9.21E-01 |
| T cell CD4+ (non-regulatory) | QUANTISEQ | -0.012 | 8.54E-01 | 9.39E-01 |
| Eosinophil | CIBERSORT-ABS | -0.010 | 8.80E-01 | 9.40E-01 |
| T cell CD4+ (non-regulatory) | XCELL | 0.011 | 8.62E-01 | 9.44E-01 |
| T cell CD8+ | EPIC | -0.010 | 8.71E-01 | 9.46E-01 |
| B cell naive | XCELL | 0.007 | 9.08E-01 | 9.48E-01 |
| Eosinophil | CIBERSORT | -0.007 | 9.09E-01 | 9.49E-01 |
| NK cell resting | CIBERSORT | 0.006 | 9.28E-01 | 9.66E-01 |
| T cell CD4+ memory resting | CIBERSORT | -0.007 | 9.17E-01 | 9.66E-01 |
| T cell CD4+ memory resting | CIBERSORT-ABS | 0.007 | 9.16E-01 | 9.66E-01 |
| NK cell resting | CIBERSORT-ABS | 0.005 | 9.34E-01 | 9.68E-01 |
| Monocyte | CIBERSORT-ABS | -0.009 | 8.88E-01 | 9.69E-01 |
| Monocyte | CIBERSORT | 0.008 | 9.01E-01 | 9.73E-01 |
| Eosinophil | XCELL | -0.004 | 9.55E-01 | 9.75E-01 |
| B cell plasma | CIBERSORT | -0.003 | 9.67E-01 | 9.77E-01 |
| T cell NK | XCELL | -0.003 | 9.63E-01 | 9.78E-01 |
| T cell CD8+ | CIBERSORT-ABS | 0.005 | 9.40E-01 | 9.85E-01 |
| T cell regulatory (Tregs) | CIBERSORT-ABS | -0.003 | 9.66E-01 | 9.91E-01 |

**Table S4. TIMERv2 analysis using different cell imputation models against 303 HGSC tumors.**
